# Atmospheric Correction of Satellite Ocean-Color Imagery During the PACE Era

**DOI:** 10.3389/feart.2019.00145

**Published:** 2019-07-26

**Authors:** Robert J. Frouin, Bryan A. Franz, Amir Ibrahim, Kirk Knobelspiesse, Ziauddin Ahmad, Brian Cairns, Jacek Chowdhary, Heidi M. Dierssen, Jing Tan, Oleg Dubovik, Xin Huang, Anthony B. Davis, Olga Kalashnikova, David R. Thompson, Lorraine A. Remer, Emmanuel Boss, Odele Coddington, Pierre-Yves Deschamps, Bo-Cai Gao, Lydwine Gross, Otto Hasekamp, Ali Omar, Bruno Pelletier, Didier Ramon, François Steinmetz, Peng-Wang Zhai

**Affiliations:** 1 Scripps Institution of Oceanography, University of California, San Diego, La Jolla, CA, United States; 2 Ocean Ecology Laboratory, NASA Goddard Space Flight Center, Greenbelt, MD, United States; 3 Science Systems and Applications Inc., Lanham, MD, United States; 4 Science Application International Corporation, McLean, VA, United States; 5 NASA Goddard Institute for Space Studies, New York, NY, United States; 6 Department of Applied Physics and Applied Mathematics, Columbia University, New York, NY, United States; 7 Department of Marine Sciences, University of Connecticut, Groton, CT, United States; 8 Laboratoire d’Optique Atmosphérique, Université de Lille, Villeneuve d’Ascq, France; 9 Jet Propulsion Laboratory, California Institute of Technology Pasadena, CA, United States; 10 Joint Center for Earth System Technology, University of Maryland Baltimore County, Baltimore, MD, United States; 11 School of Marine Sciences, University of Maine, Orono, ME, United States; 12 Laboratory for Atmospheric and Space Physics, University of Colorado, Boulder, CO, United States; 13 Naval Research Laboratory, Washington, DC, United States; 14 Pixstart, Toulouse, France; 15 Earth Science Group, Netherlands Institute for Space Research, Utrecht, Netherlands; 16 Atmospheric Composition Branch, NASA Langley Research Center, Hampton, VA, United States; 17 Institut de Recherche Mathématique, Université de Rennes, Rennes, Franc; 18 HYGEOS, Euratechnologies, Lille, France; 19 Department of Physics, University of Maryland Baltimore County, Baltimore, MD, United States

**Keywords:** ocean color, aerosols, atmospheric correction, hyper-spectral remote sensing, multi-angle polarimetry, PACE mission

## Abstract

The Plankton, Aerosol, Cloud, ocean Ecosystem (PACE) mission will carry into space the Ocean Color Instrument (OCI), a spectrometer measuring at 5nm spectral resolution in the ultraviolet (UV) to near infrared (NIR) with additional spectral bands in the shortwave infrared (SWIR), and two multi-angle polarimeters that will overlap the OCI spectral range and spatial coverage, i. e., the Spectrometer for Planetary Exploration (SPEXone) and the Hyper-Angular Rainbow Polarimeter (HARP2). These instruments, especially when used in synergy, have great potential for improving estimates of water reflectance in the post Earth Observing System (EOS) era. Extending the top-of-atmosphere (TOA) observations to the UV, where aerosol absorption is effective, adding spectral bands in the SWIR, where even the most turbid waters are black and sensitivity to the aerosol coarse mode is higher than at shorter wavelengths, and measuring in the oxygen A-band to estimate aerosol altitude will enable greater accuracy in atmospheric correction for ocean color science. The multi-angular and polarized measurements, sensitive to aerosol properties (e.g., size distribution, index of refraction), can further help to identify or constrain the aerosol model, or to retrieve directly water reflectance. Algorithms that exploit the new capabilities are presented, and their ability to improve accuracy is discussed. They embrace a modern, adapted heritage two-step algorithm and alternative schemes (deterministic, statistical) that aim at inverting the TOA signal in a single step. These schemes, by the nature of their construction, their robustness, their generalization properties, and their ability to associate uncertainties, are expected to become the new standard in the future. A strategy for atmospheric correction is presented that ensures continuity and consistency with past and present ocean-color missions while enabling full exploitation of the new dimensions and possibilities. Despite the major improvements anticipated with the PACE instruments, gaps/issues remain to be filled/tackled. They include dealing properly with whitecaps, taking into account Earth-curvature effects, correcting for adjacency effects, accounting for the coupling between scattering and absorption, modeling accurately water reflectance, and acquiring a sufficiently representative dataset of water reflectance in the UV to SWIR. Dedicated efforts, experimental and theoretical, are in order to gather the necessary information and rectify inadequacies. Ideas and solutions are put forward to address the unresolved issues. Thanks to its design and characteristics, the PACE mission will mark the beginning of a new era of unprecedented accuracy in ocean-color radiometry from space.

## INTRODUCTION

### Importance of Water Reflectance

The electromagnetic radiation (radiance) emanating from a water body at solar wavelengths, or water-leaving radiance, normalized by the incident solar irradiance at the surface defines “remote sensing” reflectance and, to an angular factor, water reflectance. Water body refers to oceans, seas, lakes, ponds, wetlands, rivers, and smaller pools of water, and the terminology marine reflectance is often used for oceanic and coastal waters. Water reflectance depends on light-matter interactions, therefore on the concentration and type of optically active constituents present in the water column and on the state of the air-water interface. The optically active constituents include water molecules, bubbles, and a variety of hydrosols and dissolved organic and inorganic materials (bacteria, viruses, phytoplankton, organic detritus, minerals, nitrates, bromides, and humic and fulvic acids). The radiative processes involved are elastic and inelastic scattering by water molecules (Rayleigh and Raman, respectively), elastic scattering by bubbles and hydrosols, absorption by water molecules and hydrosols, absorption by dissolved substances, fluorescence by phytoplankton and dissolved compounds, photoluminescence and bioluminescence by a variety of marine organisms, and Fresnel reflection/refraction at the wavy surface. These processes are generally fast, i.e., quasi instantaneous, but some are slow (phosphorescence). They interact in emitting, transmitting, absorbing, and reflecting light, contributing to the final reflectance. Water reflectance is an apparent, not intrinsic, optical property (depends on geometry), but its value is largely determined by the inherent properties of the water body, i.e., absorption and scattering coefficients, not by variations in directional illumination (angular distribution of downward radiance) or the viewing direction, providing a good—certainly convenient—way to characterize a water body.

Owing to this link to optical and biogeochemical variables, and despite the relatively small penetration depth of solar radiation in the water column, observations of spectral water reflectance, commonly referred to as “water color” (or “ocean color” in the case of marine waters), constitute a major tool to gather information about water constituents and associated processes (e.g., primary production), complementing traditional physical, biological, and chemical measurements of the euphotic zone. Spectral water reflectance gives access to chlorophyll-a, -b, and –c and other phytoplankton pigment concentrations, diffuse attenuation coefficient, euphotic depth, inherent optical properties (absorption and scattering coefficients), dissolved organic matter, total suspended matter, particulate organic and inorganic carbon, phytoplankton physiological properties (carbon-to-chlorophyll ratio, fluorescence quantum yield), phytoplankton taxonomic groups (based on size and/or pigments), and primary production (see, IOCCG, 2012). Knowing these parameters is essential to advance our understanding of the biological pump and carbon/nutrient cycling, i.e., processes that affect the ocean uptake of atmospheric carbon dioxide and the functioning of aquatic ecosystems. The information allows one to confront biogeochemical models with actual data, assimilate optical properties in ecosystem models, quantify complex evolutionary rates, determine biological-physical interactions, and clarify the photochemistry of dissolved organic matter, and it provides key water quality and ecological indicators for managing aquatic environments. Applications and societal benefits of ocean color radiometry are discussed with illustrative examples in IOCCG (2008). The retrieval methodologies exploit differences or specific features in the spectral absorption and scattering properties of water constituents that imprint on the observed reflectance, for example larger chlorophyll-a absorption in the blue than in the green to infer chlorophyll-a concentration, or spectral slope of the backscattering coefficient to estimate particle size. The inversion schemes and empirical algorithms, including many variants, are described extensively in the literature; see the reviews of IOCCG (2006) and Werdell et al. (2018) for inherent optical properties and IOCCG (2014) for phytoplankton types.

### Need to Observe Water Reflectance From Space

Due to the vastness and remoteness of the oceans, traditional observing platforms such as ships, moorings, free-drifting floats, and even aircraft, cannot collect marine reflectance data at the required spatial and temporal scales (local to global, hour to decade) to resolve the highly dynamic nature of biogeochemical phenomena. Turbulent diffusion, vertical mixing, advection, upwelling, and the life characteristics of marine organisms control these scales, resulting in complex patterns of ocean color variability (e.g., eddies, plumes, and fronts). A ship observing at a point in space and time, for example, cannot detect changes that may occur a short distance away from the station and before/after the measurements, and interpolating information from discrete stations may not capture actual variability. Only satellite instruments can provide, via water reflectance observations, a synoptic view of algal blooms, phytoplankton community structure, primary production, sediment plumes, oil spills, benthic habitats, and linkages between biology and the physical/chemical environment (e.g., Platt et al., 2008), although water reflectance is only sensitive to the surface layer (about one attenuation depth). Thanks to their global, repetitive, and long-term capability, satellite instruments constitute the only way to quantify and monitor the role of the ocean as a sink for carbon, to describe coherently inter-annual variability associated to large-scale phenomena (e.g., El Niño), to detect trends in carbon fixation, and to address how biological carbon uptake and aquatic ecosystems respond to climate change (National Research Council, 2008). Note, in this context, that marine reflectance may have a clearer signature of climate than derived quantities like chlorophyll concentration, because it integrates all the optically important water constituent alterations (Dutkiewicz et al., 2016). Depending on the applications, the satellite ocean color data requirements, in terms of spatial resolution, spectral resolution, frequency, and coverage, vary widely. For example, studies of phytoplankton phenology, carbon inventory, heat budget and ocean dynamics, and ecosystem management need global multi-spectral images at 1 km resolution every 2–3 days, but monitoring of coral reefs and sea grass beds requires multi-to hyper-spectral observations twice a year at a fine 10 m resolution over coastal and estuarine regions, and tracking harmful algal blooms necessitates frequent (hourly) hyper-spectral observations over specific areas (National Research Council, 2011). The applications are so diverse that the various, sometimes-conflicting data requirements, cannot be met with a single observing satellite (Muller-Karger et al., 2018).

### Algorithms to Retrieve Water Reflectance From Space

Water reflectance observations from space are affected by a variety of interfering processes associated with the propagation of electromagnetic radiation in the atmosphere-surface system. In clear sky conditions, these processes are gaseous absorption, molecular scattering, aerosol scattering and absorption, and water surface (Fresnel) reflection. In cloudy conditions, scattering by cloud droplets makes it very difficult to sense the surface (the cloud signal largely dominates), except when clouds are optically thin or occupy a small fraction of the pixel, i.e., their effect on pixel reflectance is less than 0.2. The influence of the atmosphere and surface must be removed in the satellite imagery to give access to water reflectance, the signal of interest (containing information about water constituents). This is commonly referred to as atmospheric correction, even though the process includes removing surface effects. Gaseous absorption is easy to handle when the satellite sensors observe in atmospheric windows (the usual case), but complicated to correct when measurements are made in absorbing bands (e.g., hyper-spectral sensor observing in the entire visible spectrum), and molecular scattering can be computed accurately. The influence of scattering by aerosols, highly variable in space and time, and of reflection by a wind-ruffled surface, which may exhibit whitecaps, is especially difficult to correct. In coastal regions, absorbing aerosols, complex water optical properties, and bottom influence (non-null water reflectance in the near infrared), and the proximity of land (adjacency effects) introduce further difficulty. Fundamentally, accurate atmospheric correction is not easy to achieve since the contribution of the water body may only represent a small fraction of the measured signal, typically 10% in the blue over clear waters and a few percent over waters rich in phytoplankton and/or yellow substances. The accuracy requirements depend on the application, but based on the experience with the Coastal Zone Color Scanner (CZCS) proof-of-concept the announced goal is that the uncertainty in the water reflectance of clear (oligotrophic) waters at 443 nm should not exceed ±5% or ±0.002 (e.g., IOCCG, 2013). Atmospheric correction algorithms, therefore, aim at achieving this goal.

The standard approach for atmospheric correction, first suggested by Gordon (1978), consists of (1) estimating the aerosol/surface reflectance in the red and near infrared where the water body can be considered as totally absorbing (i.e., black), and (2) extrapolating the aerosol/surface reflectance to the shorter wavelengths. Algorithms based on this approach have been developed successfully and employed for the operational processing of data from most satellite ocean-color sensors. In the coastal zone where waters often contain inorganic material, the assumption of null water reflectance in the red and near infrared is not valid, and improvements to the standard algorithm have been proposed. The improvements in these regions consider spatial homogeneity for the spectral ratio of the aerosol and water reflectance in the red and near infrared (Ruddick et al., 2000) or for the aerosol type, defined in a nearby non-turbid area (Hu et al., 2000). They also use a bio-optical model (Moore et al., 1999; Siegel et al., 2000; Stumpf et al., 2003; Bailey et al., 2010), exploit differences in the spectral shape of the aerosol and marine reflectance (Lavender et al., 2005), or make use of observations in the short-wave infrared, where the ocean is black even in the most turbid situations (Gao et al., 2000; Wang and Shi, 2007; Oo et al., 2008; Wang et al., 2009).

Another approach to atmospheric correction is to determine simultaneously the key properties of aerosols and water constituents of the coupled system by minimizing an error criterion between the measured top-of-atmosphere (TOA) reflectance and the output of a radiation transfer (RT) model (e.g., Land and Haigh, 1997; Chomko and Gordon, 1998; Stamnes et al., 2003; Kuchinke et al., 2009; Shi et al., 2016). This belongs to the family of deterministic solutions to inverse problems. Through systematic variation of candidate aerosol models, aerosol optical thickness, hydrosol backscattering coefficient, yellow substance absorption, and chlorophyll-a concentration, or a subset of those parameters, a best fit to the spectral top-of-atmosphere reflectance (visible and near infrared) is obtained in an iterative manner. The advantage of this single-step approach, compared with the standard, two-step approach, resides in its ability to manage situations of both Case 1 and Case 2 waters. It also works in the presence of weakly and strongly absorbing aerosols, even if the vertical distribution of aerosols, an important variable (which governs the coupling between aerosol absorption and molecular scattering), is usually not varied in the optimization procedure. A main drawback is that the inversion relies on a water reflectance model that may not represent well bio-optical variability across diverse aquatic ecosystems. Another drawback is that convergence of the minimizing sequence may be slow in some algorithms, making it difficult to process large amounts of satellite data. To cope with this issue, a variant proposed in Brajard et al. (2006) and Jamet et al. (2005) consists of approximating the operator associated to the RT model by a function which is faster in execution than the RT code, e.g., by neural networks. It may also not be easy to differentiate absorption by aerosols and water constituents like yellow substances, processes that are indistinguishable in the observed TOA signal. As a result, the retrievals may not be robust to small perturbations on the TOA reflectance. This reflects the fact that atmospheric correction is an ill-posed inverse problem; in particular, different values of the atmospheric and oceanic parameter can correspond to close values of the TOA reflectance (Frouin and Pelletier, 2015). In the context of deterministic inverse problem, stability of the solution can be obtained by regularization, but regularization strategies are not implemented in the approaches described above.

Another route is to cast atmospheric correction as a statistical inverse problem and to define a solution in a Bayesian context. In this setting, some algorithms aim at estimating, based on simulations, a function performing a mapping from the TOA reflectance to the water reflectance. In Schroeder et al. (2007), a neural network model is fitted to simulated data. A similar approach is studied in Gross-Colzy et al. (2007a,b), where the (finite-dimensional) TOA signal, corrected for gaseous absorption and molecular scattering, is first represented in a basis such that the correlation between the ocean contribution and atmosphere contribution is, to some extent, minimized. This representation of the TOA reflectance makes the function approximation problem potentially easier to solve. In these studies, data are simulated for all the observation geometries. In Pelletier and Frouin (2004, 2006) and Frouin and Pelletier (2007), the angular information is decoupled from the spectral reflectance, and atmospheric correction is considered as a collection of similar inverse problems indexed by the observation geometry. In Frouin and Pelletier (2015), the solution of the inverse problem is expressed as a probability distribution that measures the likelihood of encountering values of water reflectance given the TOA reflectance (i.e., after it has been observed). This posterior distribution is a very rich object. Its complete reconstruction is computationally prohibitive, but one may extract (approximate) expectation and covariance, which gives an estimate of the water reflectance and a quantification of uncertainty in the water reflectance estimate. This is accomplished using partition-based models. In Saulquin et al. (2016), Gaussian mixture models represent the prior distributions of reference water and aerosol reflectance spectra, and maximum a posteriori estimation (based on optimization from random initializations) is used in the numerical inversion. Performance of the statistical algorithms depends critically on prior knowledge of aerosol properties and water reflectance and noise in the measurements.

Comparing the aforementioned schemes, the chief advantage of the standard scheme is that no assumption is made about water reflectance, the signal to retrieve, except to account for the non-black reflectance of Case 2 waters in the near infrared in some variants. However, such methods make significant assumptions about the atmospheric state, and often cannot actively account for multiple scattering interactions between the atmosphere and ocean. In all the other schemes, the solution is constrained by a reflectance model with (often empirical) bio-optical relations or picked in an (relatively small) ensemble of actual spectra. The reflectance model or the ensemble of possible solutions may not be representative of actual variability, especially if the objective is general applicability, i.e., a scheme that functions adequately for all types of waters. In fact, the number of parameters to vary in optimization schemes is usually limited, otherwise convergence may be too difficult to achieve, which restricts the use of the reconstructed water reflectance.

### Atmospheric Correction Issues

The atmospheric correction algorithms so far proposed, whether empirical (standard approach, 2 steps), deterministic (spectral optimization, 1 step), or statistical (Bayesian inference, 1 step), rely on RT codes to generate look-up tables (e.g., Rayleigh scattering, aerosol optical properties) and/or simulate the TOA signal and its components. The codes have intrinsic errors depending on the way the RT equation is solved (scalar versus vector, matrix operator, doubling-adding, successive-orders-of-scattering, spherical harmonics, Monte Carlo, etc.) and how the atmosphere-surface-water system is modeled (e.g., number of layers). They employ parameterizations based on current knowledge to specify processes such as surface reflection by the agitated surface and diffuse reflection by whitecaps, but these parameterizations have uncertainties. The calculations are generally performed assuming a plane-parallel atmosphere, which yields significant errors with respect to calculations in a spherical-shell atmosphere, even at low Sun and view zenith angles. No horizontal changes in atmospheric properties are considered for slanted geometries. The “large target” formalism, in which surface reflectance is assumed homogeneous spatially, is used to express the RT equation, but this treatment is not appropriate near clouds, land, and sea ice, and in regions with high water reflectance contrast (adjacency or environment effects).

As already mentioned above, atmospheric correction is an ill-posed inverse problem, i.e., even without noise the different states of the atmosphere, surface, and water body may match well of the satellite signal. Determining the aerosol model from a somewhat arbitrary set of candidates according to the spectral dependence of the aerosol reflectance in the red, near infrared, and shortwave infrared, a key feature of the standard algorithm, does not guarantee that the selected model is the actual one. First, an aerosol model cannot be unambiguously identified from observations in the red and infrared alone, especially without polarized and multi-angular information. In this spectral range, sensitivity to small particles is limited (these particles scatter more light backward to space than large particles, i.e., they have a strong influence on the TOA signal), and it is not possible to distinguish absorbing aerosols from non-absorbing ones since the coupling between molecular scattering and aerosol absorption is ineffective. This inability to deal properly with absorbing aerosols constitutes a major drawback of the standard scheme. Second, the aerosol model selection strongly depends on the set of models to choose from. Defining the models conveniently so that they uniquely describe the range of observed spectral dependence of aerosol reflectance is not satisfactory, even though the models may be obtained from measurements (e.g., AERONET). One may argue that selecting the proper aerosol model is a secondary issue since the objective of the atmospheric correction is to retrieve water reflectance and not aerosol properties, but extrapolating aerosol reflectance to UV and visible wavelengths with an incorrect model may yield inaccurate estimates of the aerosol signal at those wavelengths (coupling terms maybe quite different). Kahn et al. (2016) examined the sensitivity of ocean color retrievals to aerosol amount and type. They showed, from comparisons with AERONET observations, that aerosol optical thickness in the blue derived from the Sea-viewing Wide Field-of-view Sensor (SeaWiFS) was overestimated, leading to systematic differences in derived water reflectance.

Another issue with aerosols is their vertical distribution, which is fixed in the standard scheme and whose variability is often not accounted for in simulations of the TOA signal used in other schemes. This parameter, however, affects significantly the coupling between scattering by molecules and scattering and absorption by aerosols, especially in the UV and blue, where molecular scattering is effective, all the more as aerosols are absorbing (effect is still non-negligible when aerosols are non-absorbing). Absorbing aerosols tend to decrease the TOA reflectance, and the decrease is more pronounced when they are located higher in the atmosphere, exhibit higher loadings, are more absorbing, or as the surface is brighter. In the presence of such aerosols, using a fixed distribution may result in large, unacceptable errors on water reflectance retrievals. Depending on vertical structure, the estimates by the standard scheme may be lower (underestimation) by as much as 10 times the inaccuracy requirements for biological applications, or even be negative as revealed in many studies, theoretical and experimental. Neglecting the effect of aerosol absorption on atmospheric transmittance, which lowers its value, further contributes to underestimating water reflectance (when the retrieved water signal is positive). Most satellite ocean-color sensors, however, do not have the capability to provide information about aerosol altitude.

It is often assumed that the color of a water body can be observed from space only over areas not contaminated by Sun glint and free of clouds. The thinking, based on “common sense” visual observations, has been that the presence of even a small amount of glint or a thin cloud prevents utilization of the data. Standard atmospheric correction algorithms, therefore, apply strict glint and cloud filters, usually a radiance or reflectance threshold applied to measurements in the near infrared (typically 0.03 on the Rayleigh-corrected reflectance). Such threshold may also exclude regions with highly scattering hydrosols under clear conditions. In general, about 10–15% of the observed pixels pass through the glint and cloud filters. As a result, Level-2 daily products are very patchy, and weekly Level-3 products show many areas with no information. This limits considerably the utility of satellite water reflectance observations for operational oceanography. In the open ocean, global coverage every three to five days is necessary to resolve seasonal biological phenomena such as phytoplankton blooms. In coastal waters, wind forcing creates “events” (e.g., upwelling) that occur every two to ten days, and one-day coverage is the requirement for resolving the event time scale. These requirements are not achieved with the present satellite systems and state-of-the-art standard algorithms. Some techniques are promising, however, such as those proposed by Steinmetz et al. (2011) and Gross-Colzy et al. (2007a), which either exploit the fact that Sun glint and cloud signals are smooth spectrally and can be well represented by a simple polynomial or select the principal components of the TOA signal that are less influenced by atmospheric and surface effects. Accurate retrievals may be obtained by relaxing the reflectance threshold in the near infrared to 0.2, with the potential of increasing daily spatial coverage by over 50% in many areas.

The performance of one-step algorithms depends on the accuracy of the water reflectance model, i.e., its ability to describe expected conditions, and/or prior knowledge of the variables to retrieve. Many of these models are developed with simulated data sets that may not represent the diversity and complexity of the real ocean. The available field data sets to specify modes of variability and prior distributions, however, are too few. They do not sample adequately many regions (e.g., the Southern Oceans) and biogeochemical regimes, they are less frequent in winter, and they are often incomplete (e.g., limited spectral range, inherent optical properties not measured concomitantly). Hyper-spectral data that incorporates the large bio-optical variability in absorption and backscattering of different assemblages of phytoplankton, particularly in complex coastal and inland waters, is lacking. Most of the data are acquired in cloudy conditions, but satellite retrievals are generally obtained under clear skies. Depending on cloudiness, bio-optical relations may be different, as well as the quality (spectral, angular) of available sunlight. In other words, the domain of acceptable solutions may not be represented properly by existing data sets. Future measurement programs will contribute to a more complete and broader knowledge, but this will probably remain insufficient. Additional information about space and time variability of atmospheric and oceanic variables, possibly from coupled biogeochemical-physical dynamic models (regional, global), will improve retrieval accuracy.

### Opportunities With PACE

The current generation of ocean color sensors, e.g., MODerate resolution Imaging Spectrometer (MODIS), Visible Infrared Imaging Radiometer Suite (VIIRS), and Ocean and Land Color Instrument (OLCI), Geostationary Ocean Color Imager (GOCI), and Second generation GLobal Imager (SGLI), provides limited spectral information on water reflectance, i.e., observations in a few spectral bands in the visible and near infrared. This makes it difficult to distinguish water constituents (e.g., phytoplankton species or groups), to quantify their relative abundance, and to infer ecosystem properties/processes. The retrieval of water reflectance, based on spectral information alone (visible to near infrared, sometimes including shortwave infrared), is good, but not sufficiently accurate in the coastal zone and over inland waters, where water and air properties are highly variable and intricate, aerosols are absorbing, and adjacency effects are substantial (see section Atmospheric Correction Issues). The PACE mission will carry a primary sensor, the Ocean Color Imager (OCI), and two multi-angle polarimeters, the Spectrometer for Planetary Exploration (SPEXone) and the Hyper-Angular Rainbow Polarimeter (HARP2). OCI is a hyper-spectral radiometer measuring at 5nm resolution in the UV to NIR (350 to 885 nm) and possibly higher spectral sampling in selected spectral intervals, with additional lower resolution bands at 940, 1,038, 1,250, 1,378, 1,615, 2,130, and 2,260nm. SPEXone will measure at 5 viewing angles from 385 to 770 nm in 2–5 nm steps, with reduced spectral resolution (10–40 nm) for polarization. HARP2 will measure polarization at 10 viewing angles in spectral bands centered on 441, 549, and 873 nm, and 60 angles for a band centered at 669 nm. Polarimetric accuracy, in terms of Degree of Linear Polarization (DoLP), is expected to be 0.003 for SPEXone and <0.01 for HARP2. Swath width (spatial coverage) is 1,500 km for both OCI and HARP2 and 100 km for SPEXone. This instrument package promises to advance our knowledge of water ecosystems and biogeochemistry to an unprecedented level, not only because the high spectral resolution allows one to retrieve more accurately multiple water-column and benthic constituents and separate a larger number of end members, or because polarization measurements can aid in the characterization of hydrosols, but also because of the great potential of combining spectral, directional, and polarized information to improve atmospheric correction, i.e., water reflectance estimates.

Extending the TOA observations to the UV, where aerosol absorption is effective, using the SWIR, where even the most turbid waters are black and sensitivity to the aerosol coarse mode is higher than at shorter wavelengths, and measuring at hyper-spectral resolution in the oxygen A-band to estimate aerosol altitude would allow, at least in principle, a more accurate atmospheric correction. Measuring in spectral intervals where solar irradiance exhibits sufficiently large variations and Raman scattering can be assumed constant, the Raman signal can be separated from the elastic signal. This would improve bio-optical algorithms in which Raman scattering effect is not taken into account and allow retrieval of information about absorbing material in clear waters. Multi-angle and polarized measurements, sensitive to aerosol properties (e.g., size distribution, index of refraction), would further help to specify the aerosol model. This may be accomplished by constraining the domain of possible aerosol types in a classic atmospheric correction scheme or, if the information is sufficiently accurate, by directly computing the aerosol scattering effect. The sensitivity of polarized reflectance to aerosol type has also the potential to improve inversion schemes that aim at retrieving simultaneously air and water properties. Using multi-angular information alone (i.e., without polarization), already possible with the Multi-angle Imaging SpectroRadiometer (MISR) and the Sea and Land Surface Temperature Radiometer (SLSTR), may also improve atmospheric correction in the presence of absorbing aerosols, and using the non-polarized or plane-parallel components of the TOA signal instead of the total signal may reduce the effect of Sun glint and boost the contribution of the water signal. Many of the ideas and approaches briefly exposed above have yet to be fully developed, tested, and evaluated, but they strongly suggest that most of the atmospheric correction issues associated with current ocean-color sensors (see section Atmospheric Correction Issues) will be solved during the PACE era. One important task, apart from technical studies, is to devise a strategy for atmospheric correction that ensures continuity and consistency with past and present missions while enabling full exploitation of the new PACE dimensions and capabilities.

### Contents of the Study

In the following, the history and progress of atmospheric correction during the last two decades (i.e., since the beginning of the EOS era) is recounted, state-of-the-art approaches and algorithms are described, and possibilities/improvements in view of new knowledge and future missions, in particular PACE, are examined. In section Heritage Atmospheric Correction Algorithm, the standard 2-step heritage algorithm, from which operational ocean color products are currently generated, is described. Adjustments envisioned to deal with hyper-spectral measurements that extend to the UV (case of OCI) are proposed and challenges are discussed. In section Alternative Algorithms, alternative algorithms, deterministic or statistical, that utilize simultaneously all available information, are presented, and their ability to deal with situations that cannot be handled by the heritage algorithm (e.g., absorbing aerosols) is emphasized and illustrated. In section Enhancements Using Multi-angular and/or Polarimetric Information, the benefits of multi-angular and polarimetric observations to determine aerosol properties or enhance the contribution of the water body to the TOA signal, and of using these observations synergistically with multi- or hyper-spectral information, are identified. In section Improvements Using “Super-Sampling” in Selected Spectral Intervals, the super-sampling capability of the OCI in some spectral intervals is investigated to estimate ocean Raman scattering and improve retrievals of chlorophyll fluorescence and aerosol altitude. In section Significant Issues, issues that remained to be addressed satisfactorily, such as adjacency in a “large target” RT formalism, whitecaps and underwater bubbles, Earth’s curvature, atmospheric horizontal/vertical heterogeneity, and observing in the UV where the atmosphere is optically thick and multiple scattering and coupling processes makes atmospheric correction especially difficult, are discussed and possible avenues considered. In section Strategy for Atmospheric Correction, a strategy for atmospheric correction is devised, that accounts for the accomplishments of the previous decades using the heritage algorithm and at the same time fully integrates the qualities of alternative schemes and the new PACE capabilities. In section Conclusions, finally, the recent developments in atmospheric correction are summarized, the expected improvements with the PACE instrumentation are highlighted, concerns and remaining gaps are reiterated, and research directions are suggested to address challenges and achieve a new level of accuracy in future satellite water reflectance products. All the figures of the study are provided separately online at: https://www.frontiersin.org/articles/10.3389/feart.2019.00145/full#supplementary-material.

## HERITAGE ATMOSPHERIC CORRECTION ALGORITHM

### Approach

The heritage approach to the retrieval of ocean bio-optical properties from satellite radiometric observations, as historically employed by all international space agencies and operational agencies, is a two-step process wherein the atmosphere (and surface contributions) are first removed using minimal assumptions about the water optical properties (Fukushima et al., 1998; Antoine and Morel, 1999; Ahn et al., 2012; Mobley et al., 2016), and the resulting spectral water-leaving radiances are then used to infer information on water column optical properties and constituent concentrations (e.g., O’Reilly et al., 1998; Morel and Maritorena, 2001; Hu et al., 2012; Werdell et al., 2013). Step one, the atmospheric correction process, is based on finding the solution to a set of deterministic models that enable the removal of atmospheric path and surface effects from the top of atmosphere (TOA) signal. The primary challenge is to determine the contribution of aerosols to the atmospheric path radiance, which is highly variable and thus must be inferred from the observations. The approach takes advantage of the very strong absorption property of water in the NIR/SWIR spectral range (longward of 750 nm) to separate the atmospheric and oceanic signal from the satellite observations (Gordon and Wang, 1994; Antoine and Morel, 1999).

This two-step approach assumes the additive property of light, where the spectral signal or radiance measured at the top of atmosphere (TOA) by a satellite sensor, *L*_*TOA*_, is the summation of the radiance contribution from each component of the atmosphere-ocean (AO) system. Specifically,

(1) $$L_{TOA}(\lambda )=L_{atm}(\lambda )+L_{surf}^{TOA}(\lambda )+L_{w}^{TOA}(\lambda )$$

where *L*_*atm*_ (λ) is the radiance contribution due to scattering and absorption of air molecules and aerosols, $L_{\text{surf}}^{TOA}(\lambda )$ is the contribution of light reflected from the ocean surface and propagated to the TOA, and $L_{w}^{TOA}(\lambda )$ is the subsurface ocean radiance that is transmitted through the ocean surface and propagated to the TOA. Note that the dependence on radiant path geometry from sun to surface and back to sensor (i.e., Sun and view zenith angles and relative azimuth angles (*θ*_*S*_, *θ*, *ϕ*), is not shown here for brevity. Equation (1) can be further partitioned into multiple components of the atmosphere and ocean surface. The atmospheric path radiance, *L*_*atm*_ (λ), for example, can be treated as the summation of the scattering by non-absorbing air molecules, *L*_*r*_(λ), which is well characterized as pure Rayleigh scattering, and the scattering by the aerosol particles and aerosol-molecule coupling processes, *L*_*a*_ (λ), which depends on aerosol morphology, size distribution, concentration, and chemical composition. These atmospheric path radiance contributions are then modulated by losses due to transmittance through the absorbing atmospheric gases, *T*_*g*_(λ), such as ozone, oxygen, and water vapor to compute the TOA contribution. Similarly, the surface radiance term at TOA, $L_{\text{surf}}^{TOA}(\lambda ),$ can be treated as the summation of the light diffused by whitecaps and foam on the ocean surface, *L*_*wc*_(λ), that propagates through the atmosphere and is therefore modulated by the atmospheric total (i.e., direct plus diffuse) transmittance along the surface-to-sensor path, *t*_*u*_ (λ), and the ocean surface glint (specular reflection of the Sun), *L*_*g*_(λ), that is also propagated through the atmosphere and modulated by the direct atmospheric transmittance, *T*_*u*_ (λ), along the specular direction, with both surface contributions also reduced by *T*_*g*_ (λ) before reaching the TOA. The primary quantity of interest for ocean color is the water-leaving radiance, *L*_*w*_(λ), which is the subsurface radiance after transmission through the air-sea interface. *L*_*w*_ (λ) is also modulated by *t*_*u*_ (λ) and *T*_*g*_ (λ) when measured at TOA. The total radiometric contribution of the AO system can thus be described as:

(2) $$L_{TOA}(\lambda )=[L_{r}(\lambda )+L_{a}(\lambda )+t_{u}(\lambda )L_{wc}(\lambda )\;\;+T_{u}(\lambda )L_{g}(\lambda )+t_{u}(\lambda )L_{w}(\lambda )]T_{g}(\lambda )$$

This formulation presumes that (1) the fraction of the sea covered by whitecaps is small and can be neglected, (2) the contribution of diffuse reflected skylight off the sea surface is accounted for in the *L*_*r*_(λ) and *L*_*a*_(λ) terms, (3) the surface is homogenous spatially, and (4) gaseous absorption and scattering processes are decoupled. The goal of the heritage atmospheric correction (AC) approach is to accurately estimate each radiance and transmittance term in Equation (2) so that the surface and atmospheric path radiance contribution can be subtracted from the observed TOA radiance to retrieve *L*_*w*_ (λ). To remove the time variation of incident direct solar irradiance, Equation (2) can be written in terms of reflectance as:

(3) $$\rho_{TOA}(\lambda )=[\rho_{r}(\lambda )+\rho_{a}(\lambda )+t(\lambda )\rho_{wc}(\lambda )\;\;+T(\lambda )\rho_{g}(\lambda )+t(\lambda )\rho_{w}(\lambda )]T_{g}(\lambda )$$

In this expression, *t* (λ) = *t*_*u*_ (λ) *t*_*d*_ (λ) and *T* (λ) = *T*_*u*_ (λ) *T*_*d*_ (λ) where *t*_*d*_ (λ) and *T*_*d*_ (λ) are total and direct downward atmospheric transmittance along the Sun-to-surface path, respectively, *ρ*_*TOA*_ (λ), *ρ*_*r*_ (λ), and *ρ*_*a*_ (λ) are *L*_*TOA*_ (λ), *L*_*r*_ (λ), and *L*_*a*_ (λ) normalized by *E*_*s*_(λ)*cos*(*θ*_*s*_)/*π*, *ρ*_*f*_ (λ) and *ρ*_*w*_ (λ) are *L*_*wc*_ (λ) and *L_w_* (λ) normalized by *E*_*s*_*(λ)cos(θ_s_)t_d_(λ)/π*, and *ρ*_*g*_ (λ) is *L*_*g*_ (λ) normalized by *E*_*s*_*(λ)cos(θ_s_)T_d_(λ)/π*, where *E*_*s*_(λ) is extraterrestrial spectral solar irradiance corrected for Earth-Sun distance. Thus in Equation (3) the signal to retrieve is *ρ*_*w*_(λ), the water reflectance just above the surface, hereafter referred to as water reflectance for short. Remote sensing reflectance, *R*_*rs*_(λ), often used instead of *ρ*_*w*_(λ), is defined as *R*_*rs*_(λ) = *ρ*_*w*_*(λ)*/*π*.

### Procedures

#### Absorbing Gas Correction

Several gases in the atmosphere, including O_2_ (Oxygen), O_3_ (Ozone), NO_2_ (Nitrogen Dioxide), H_2_O (Water Vapor), CO (Carbon Monoxide), N_2_O (Nitrous Oxide), CH_4_ (Methane), and CO_2_ (Carbon Dioxide), absorb the light along the path radiance. In heritage multispectral sensors such as MODIS or MERIS, the spectral bands used for ocean color are generally situated to avoid the strongest absorption features of O_2_ and H_2_O (i.e., the spectral bands are located in atmospheric transparency windows), however O_3_ and NO_2_ absorb light over a broad spectral range. For a given column concentration of gas measured in number density per unit area, equivalent to Dobson units (DU), and its vertical profile (pressure, temperature, and volume mixing ratio (VMR), the transmittance of gas can be calculated. Because ozone is concentrated very high in the atmosphere, the O_3_ transmittance can be accurately estimated using the Beer-Lambert Law and the geometric air mass. The coupling between the absorption of gases and the scattering by non-absorbing gases and aerosols is not significant in this case. NO_2_ absorption, however, occurs in the tropospheric layer of the atmosphere, and near a source region that is spatially limited, thus the coupling between the absorption and scattering can be significant and the Beer-Lambert Law treatment is not appropriate (Ahmad et al., 2007). Although multi-spectral sensor bands are typically selected to avoid the strong absorption features, the nominal spectral bands for sensors such as MODIS or SeaWiFS can still suffer from significant out-of-band (OOB) sensitivity to these gases, thus requiring sensor-specific corrections (Ding and Gordon, 1995).

#### Molecular Scattering Correction

Light scattering by air molecules in the atmosphere, mainly N_2_ and O_2_, can be accurately modeled following the principles of Rayleigh scattering. Typically, a vector radiative transfer (VRT) code is used to generate a set of Look-up-tables (LUTs) of spectral Rayleigh scattering as a function of radiant path geometry and wind-driven ocean surface roughness (e.g., Ahmad and Fraser, 1982 is used for all operational processing by NASA). The LUTs include the contribution of photons that are reflected by the wavy interface after being either scattered in the atmosphere and then transmitted (directly and diffusely) to the TOA or directly transmitted through the atmosphere and then diffusely transmitted to the TOA. Because this contribution, commonly referred to as skylight reflected radiance, is enhanced in violet and UV wavelengths (due to more effective Rayleigh scattering), its correction is particularly critical in retrieving accurate water reflectance moving from the visible into UV wavelengths. The VRT model generally assumes an atmosphere-ocean system with discrete wind-driven ocean surface roughness states described by the analytical, azimuthally symmetric Cox-Munk slope distribution model (Cox and Munk, 1954). A required input to the VRT code is the Rayleigh optical thickness of the atmosphere at each sensor spectral wavelength, which can be computed from existing models (e.g., Bodhaine et al., 1999) and convolved with the relative spectral response of each spectral band pass.

#### Sun Glint and Whitecap Correction

Sun glint is a strong signal that reflects off the flat ocean surface in the specular direction. With increasing wind speed, the surface roughness also increases, thus a glitter pattern appears. The glitter pattern is the reflected Sun glint in the off-principal plane of the Sun when a wave facet is tilted. The glitter pattern spreads off the principal plane proportionally to the increasing wind speed. Some ocean color sensors, such as SeaWiFS, have tilting capability that avoids the detection of light in the principal plane of the Sun, thus avoiding the specular reflection of the ocean surface. Even so, a significant residual Sun glint can reach the sensor, especially near the edges of the glint region in and near the principal plane. To estimate this glint reflectance contribution, *ρ*_*g*_ (λ), the heritage AC algorithms typically rely on the Cox-Munk model (Cox and Munk, 1954).

The surface contribution from whitecaps and foam to the TOA radiance is typically estimated from whitecap reflectance and a wind speed dependent estimate of the fractional coverage of whitecaps on the ocean surface. The whitecap reflectance is assumed Lambertian and wavelength dependent (Koepke, 1984; Frouin et al., 1996; Stramska and Petelski, 2003). Opposite to the specular glint, the whitecap reflectance contribution at the TOA is diffuse in nature, and thus modulated by the total (direct + diffuse) transmittance of the atmosphere, *t*(λ).

#### Aerosol Scattering Correction

Sources of the aerosol particles suspended in the atmosphere are natural, such as dust, sea-salt, mineral dust, or volcanic emissions, or anthropogenic in origin, such as sulfates, organics and smoke from biomass burning or urban/industrial output. Aerosols vary in morphology, size distribution, scattering properties, and concentration. Analogous to the Rayleigh correction, an aerosol LUT is typically used to remove the aerosol contribution to the TOA radiance. The LUT defines a set of aerosol models, where the aerosol correction algorithm determines the most appropriate aerosol type (aerosol model) for a given pixel, to perform the correction. Different aerosol types are defined based on a bimodal size distribution of fine and coarse particles of specified refractive index, each with a lognormal distribution. NASA’s operational aerosol LUTs, for example, are based on aerosol modeling by Ahmad et al. (2010). In this case, the aerosol models were derived to match the observations from the Aerosol Robotic Network (AERONET), with aerosol radii, refractive index, and fine-mode fraction parameterized as an explicit function of the relative humidity (30, 50, 70, 75, 80, 85, 90, and 95%). In constructing the LUT, the microphysical properties of the aerosols are assumed to be homogenous spheres, and therefore follow the scattering by Mie theory. VRT simulations are performed to characterize the aerosol radiance contribution to the TOA signal for each aerosol type, assuming a standard aerosol vertical profile for marine aerosols [e.g., from Shettle and Fenn (1979)].

A primary assumption for the determination of aerosol type and concentration within the heritage atmospheric correction (AC) approaches is that water-leaving radiance contributions within the NIR/SWIR spectral regime can be considered negligible due to the strong water absorption (Gordon and Wang, 1994; Antoine and Morel, 1999). This is referred to as the dark pixel assumption and is valid for most of the global ocean, where the hydrosol scattering is insignificant in the NIR/SWIR part of the spectrum. For highly productive or turbid conditions, most common in coastal and inland waters, methods have been developed to estimate the water-leaving radiance in the NIR regime through iterative solutions and model extrapolation (e.g., Moore et al., 1999; Bailey et al., 2010), or switching algorithms are employed to restrict to the more strongly absorbing SWIR spectral range in the presence of turbid waters (Wang et al., 2009).

#### Advantages and Limitations

The primary advantage of the two-step heritage AC approaches is that they do not require strong assumptions about the magnitude or spectral distribution of the ocean radiance contribution. The approaches are limited, however, by strict reliance on the NIR-SWIR spectral regime to determine the aerosol contribution, which is then extrapolated to the visible spectral bands based on a pre-determined set of aerosol models. The heritage approaches fail in the presence of strongly absorbing aerosols, and they are challenged in complex or highly productive waters where the water-leaving signal in the NIR-SWIR spectral regime can be significant. A small uncertainty in the determination of the aerosol type in the NIR-SWIR spectral regime can be magnified when extrapolating that information to the blue spectral range.

##### Absorbing aerosols

The ability to account for the presence and impact of absorbing aerosols, such as terrigenous dust and black or biogenic carbon, is a long-standing challenge in ocean color AC (Li et al., 2003; Schollaert et al., 2003; Nobileau and Antoine, 2005; Antoine and Nobileau, 2006; Ransibrahmanakul and Stumpf, 2006). Absorbing aerosols are common in coastal waters, due in particular to air pollution, but they can be encountered over vast areas of the open oceans (Kaufman et al., 1997; Remer et al., 2008; Colarco et al., 2014). Large plumes of Saharan dust, for example, are routinely transported across the Atlantic and deposited in the Caribbean, and smoke plumes from biomass burning off the west of African can be found well into the equatorial Atlantic. Since the heritage AC algorithms rely exclusively on the NIR/SWIR spectral regime and thus cannot identify and account for the presence of strongly absorbing aerosol types, the water reflectance in such cases will typically be underestimated, which leads to an overestimation in the chlorophyll concentration (Gordon, 1997; Gordon et al., 1997; Chomko and Gordon, 1998; Schollaert et al., 2003; Nobileau and Antoine, 2005; Antoine and Nobileau, 2006; Shi and Wang, 2007).

Heritage OC sensors observe the ocean at a single viewing angle and at a limited set of spectral bands, and thus the AC algorithm is tasked with solving an underdetermined, ill-posed AO system. Additional constraints on the aerosol microphysical properties, such as the hygroscopic growth determined by relative humidity, helps in determining the type of aerosol for the AC, but absorbing aerosols have spectral absorbing signatures that are similar to other optically active components of the ocean such as colored dissolved organic matter (CDOM) and minerals. Additionally, most absorbing aerosol species absorb in the shorter (UV-visible) wavelengths, thus the NIR/SWIR heritage approach to aerosol determination is not sensitive to their optical variations (Kahn et al., 2016). Furthermore, there is no consensus on the range of microphysical properties that must be considered for absorbing aerosols, due to the variation in their morphology, chemical composition, etc., and knowledge of the aerosol vertical distribution, which is critical to properly quantify the impact of aerosol absorption, cannot be determined from heritage sensor systems.

Figure 1 shows the sensitivity of TOA radiance to changes in aerosol layer height (i.e., location in the vertical column) relative to a 3 km layer height. The analysis was performed using the Ahmad and Fraser (1982) VRT code, for a specific solar and viewing geometry at 412 nm and for varying single scattering albedo of an assumed dust aerosol model detailed in Ahmad and Franz (2014). Chlorophyll-a concentration, *Chl-a,* is 0.3 mgm^−3^. From Figure 1, it can be seen that the TOA reflectance is overestimated for absorbing aerosol layers lower than 3 km, while it is underestimated when the aerosol is higher in the atmosphere. The relative magnitude of change depends on the amount of aerosol absorption and the optical thickness.

At higher optical thickness, the TOA reflectance shows more sensitivity to absorbing aerosol layer height on the order of ~1 to 4%, while at small optical thickness, the sensitivity is <1%. It is important to note that unknown layer height of the absorbing aerosol can lead to a large error in the derived water reflectance from the AC process. Additionally, when absorbing aerosols are not detectable from satellite observations, their treatment as non-absorbing in the AC can lead to overestimation of the aerosol radiance, which is demonstrated in Figure 2. Based on VRT simulations, the heritage AC algorithm retrieves an overestimated aerosol reflectance (blue line) in the presence of absorbing aerosols, while the true reflectance is lower (red line), and thus an overestimated aerosol contribution leads to an underestimated remote sensing reflectance. These over/under estimation errors increase with decreasing wavelengths, thus compromising efforts to retrieve water reflectance into the UV spectral regime.

### Plane-Parallel RT Limitation and Pseudo-Spherical RT Correction

Presently all atmospheric correction LUTs used for ocean color retrievals assume that the atmosphere consists of plane parallel layers, which are inhomogeneous vertically but homogeneous and infinite horizontally. This generally limits applicability to solar angles less than 70° and sensor view angles less than 50°. To extend the solar and view angle range, the sphericity of the Earth atmosphere must be accounted for in the RT simulations. Herman et al. (1995) have carried out a detailed comparison of TOA radiance for spherical and plane-parallel atmospheres and concluded that there is a strong agreement between the two methods except at highly oblique angles. For a spherical atmosphere, the RT calculations account for sphericity in both the incoming solar beam and the outgoing scattered beam. Vector RT calculations (with multiple scattering and polarization) are computationally expensive. If retrievals at very large solar zenith angles (>80°) and large view angles (>50°) are not required, then one can carry out the vector RT calculations for a spherical atmosphere in single scattering approximation, and use full multiple scattering for a plane parallel atmosphere (Caudill et al., 1997). Another simplification is to account for sphericity in the incident solar beam only, which is done by computing the air mass factor in a spherical model atmosphere, and perform the rest of the calculations in a plane parallel atmosphere. This is generally known as the pseudo-spherical correction (Deluisi andMateer, 1971).

Figure 3 shows how the Sun zenith angle varies as the solar beam traverses through a spherical model atmosphere. It is *θ*″ at the top of layer 1 (TOA), *θ*′ at the top of layer of layer 3 and *θ* at the Earth surface. For a plane-parallel model atmosphere, the Sun zenith angle for all the layers would be equal to *θ*. Figure 4 shows the difference in TOA reflectance as simulated with both plane-parallel and pseudo-spherical assumptions, for a Rayleigh model atmosphere at 380 nm in the direction *θ* =30°, *ϕ* =144°. The figure shows that, for Sun zenith angle <50°, the difference is very small (<0.05%), but increases rapidly at higher Sun zenith angles. In the nadir direction, it is −0.12, −0.53, and −1.53% for Sun zenith angles of 60, 72, and 78°, respectively. For accurate water reflectance retrievals at solar zenith angles above 50, these results suggest that future AC algorithms should utilize spherical or, at a minimum, pseudo-spherical VRT simulations in the retrieval algorithm or in generating any required atmospheric LUTs.

### From Multi-Spectral to Hyper-Spectral Remote Sensing

#### Challenges

The AC process must estimate and remove the atmospheric path radiance contribution due to the Rayleigh scattering by air molecules and scattering by aerosols from the measured TOA radiance, account for surface contributions, and correct for reflection and refraction of the air-sea interface. For a hyper-spectral sensor, the heritage AC approach can largely be employed as-is, by simply extending the Rayleigh and aerosol scattering tables and surface reflectance models to the hyper-spectral domain. A primary issue, however, is the influence of absorbing gases.

Heritage multispectral ocean color sensors such as SeaWiFS, MODIS, MEdium Resolution Imaging Spectrometer (MERIS), VIIRS, OLCI, and SGLI, detect the light at specific wavelengths or bands. These bands are strategically located to provide sufficient spectral information to enable estimation of the inherent optical properties and optically active constituent concentrations in the water column, while also being located in atmospheric window regions where the atmospheric transmittance is maximized. The window regions are selected primarily to avoid the absorption of water vapor in the atmosphere, which is highly variable and thus difficult to correct, and the narrow but strong spectral absorption features of oxygen. To fully utilize the greater spectral information available from a hyper-spectral ocean color sensor, as needed for emerging science such as the detection of phytoplankton types and phytoplankton community structure (a primary goal of the PACE mission), the observed signal must be corrected for these absorbing gases.

Figure 5 shows the atmospheric gas transmittance calculated at 5-nm spectral resolution from UV to SWIR. The transmittance includes ozone, oxygen, and water vapor (blue solid line), while the red circles are located at MODIS bands. As shown, the MODIS bands are located in the spectral window regions, where the impact of absorption by oxygen and water vapor is minimized. For a continuous 5-nm sampling, however, as expected from the PACE OCI sensor, several bands in the visible wavelengths will suffer absorption by strong spectral features of water vapor and oxygen. This is especially true in the red (600–720 nm), which is critical for distinguishing phytoplankton types and quantifying natural fluorescence of phytoplankton chlorophyll. Production of a continuous 5-nm sampling of visible remote sensing reflectance over the ocean is a primary goal to the PACE mission, thus proper compensation of absorbing gas features is needed.

To make full use of a hyper-spectral instrument for ocean color, the effect of water vapor must be estimated and corrected. This is challenging due to the complexity of the atmospheric water vapor profile, the spectrally variable nature of the absorption features, and the spatial heterogeneity of the water vapor concentration (Kaufman and Gao, 1992; Gao et al., 2000, 2009). Challenges also arise in the UV spectral range, where the AC is difficult due to the significant contribution of the atmospheric scattering, and the strong impact from absorbing aerosols.

#### Hyper-Spectral Atmospheric Correction

Similar to the heritage multispectral atmospheric correction, pre-computed hyper-spectral look-up tables (LUT) can be generated using VRT simulations to model the Rayleigh and aerosol contributions. The hyper-spectral optical properties used in the VRT simulations, such as the extraterrestrial solar irradiance, Rayleigh optical thickness, and the depolarization factors, would be optically weighted for each hyper-spectral band based on the measured spectral response functions. Hyper-spectral glint and whitecap radiance contributions can be modeled based on ancillary wind speed and observing geometry, as they are for the heritage multispectral AC approaches. The hyper-spectral aerosol type and concentration can also be estimated using heritage methods (Fukushima et al., 1998; Antoine and Morel, 1999; Ahn et al., 2012; Mobley et al., 2016), and similarly coupled with an iterative bio-optical model to separate the scattering contributions from aerosols and water-column constituents in turbid (high-scattering) waters (Moore et al., 1999; Bailey et al., 2010).

Since the heritage AC does not perform water vapor correction, except for out-of-band effects using the Gordon (1995) method, the algorithm must be enhanced to include such a correction, if spectral regions with modest to large water vapor features are to be used for ocean color science (i.e., 600–710 nm). Recently, the heritage AC algorithm maintained by NASA (Mobley et al., 2016) was extended to include the hyper-spectral algorithm of ATmospheric REMoval (ATREM) for the estimation and correction of water-vapor transmittance (Gao and Davis, 1997), as detailed in Ibrahim et al. (2018). ATREM calculates the transmittance using the HITRAN 2012 (Rothman et al., 2013) database at 0.05 cm^−1^ wavenumber spectral resolution, which is down-sampled to the sensor spectral resolution. ATREM has the capability to estimate the column water vapor amount (CWV) [given a water vapor volume mixing ratio (VMR) profile], and to correct for the water vapor absorption along the radiant path. It does so by using a 3-band ratio technique utilizing two atmospheric window channels around one strongly absorbing water vapor band as shown in Figure 6.

The trough in the TOA reflectance at the water vapor band (e.g., near 940 and 1,130 nm in Figure 6) relative to the two window bands is a direct measure of the water-vapor transmittance loss along the path that the light traveled, which can be correlated to the water-vapor amount. Strong water-vapor absorption features such as these are used to estimate the water-vapor transmittance, which is then extrapolated to the visible regime to correct for the weaker water-vapor absorption features in the 600–710 nm region. Note that the 3-band ratio technique assumes the surface reflectance is spectrally monotonic. Any surface spectral features within the spectral windows can lead to erroneous correction for water vapor transmittance losses, but ocean reflectance is generally monotonic in the red/NIR part of the spectrum, except in bloom conditions (Gower et al., 2008; Doron et al., 2011), very turbid (i.e., sediment-laden) waters (Doron et al., 2011; Knaeps et al., 2015), optically shallow waters (Fogarty et al., 2018), or when the sea is covered by floating vegetation (Dierssen et al., 2015a; Kudela et al., 2015) or whitecaps (Frouin et al., 1996).

For water vapor correction over oceans, the very strong water-vapor absorption features at 940-nm and at longer wavelengths can actually be too strong, as the small surface signal (typically 0.02 in reflectance) is completely absorbed before reaching the sensor. To demonstrate this, and identify alternative band selection for water vapor retrieval over oceans, a radiative transfer study was performed using the Monte Carlo-based VRT code MYSTIC (Mayer, 2009; Emde et al., 2016). The code was used to simulate the polarized TOA radiance for a simple Rayleigh atmosphere and absorbing flat ocean, at a very high wavenumber spectral resolution of 1 cm^−1^ (~0.01nm in VIS). Water vapor was assumed to be the only absorber in the atmosphere, and was coupled with the scattering in the VRT simulations. The simulation runs were calculated for 0.3, 0.5, 0.7, 1, 1.3, 1.5, 1.7, 2, 2.3, 2.5, 2.7, and 3 cm CWV at geometries permuted from 10° to 50° solar and viewing zenith angle with 10° step, while the relative azimuth was fixed to be 90° composing a total of 300 cases. The water vapor profile was assumed to be the US standard 1976 and the water vapor absorption coefficients were obtained from the HITRAN 2012 database (Anderson et al., 1986; Rothman et al., 2013).

Figure 7 shows the scatter plot between the assumed CWV in the VRT simulations and the retrieval of CWV by the AC algorithm. In this analysis, the water body was assumed black (not reflecting). We used three modes of retrieval. The first is using the average CWV retrieval using the strongly absorbing 940 nm band and the less sensitive 820 nm band shown in blue circles, with the error bar of 1 standard deviation due to changes in the simulated geometries. With this combination, the CWV retrieval shows a strong underestimation at higher water vapor concentrations. This is due to the combination of a weak ocean signal (in that case ocean surface reflectance) and a strongly absorbing 940-nm water vapor feature that leads to loss of sensitivity to further increases in CWV. The retrieval for CWV less than 1 cm, however, shows good performance suggesting that the 940-nm channel can be utilized to detect and retrieve small amounts of water vapor in the atmosphere, while its sensitivity saturates for larger than 1 cm CWV. The second mode of CWV retrieval shown in green circles, using an average of CWV retrievals at both 720 and 820 nm, shows very good performance along the whole dynamic range of CWV. Although the retrieval shows a slight bias at small CWV values, the impact of erroneous (biased) CWV at low values is less significant on the R_rs_ of the ocean, especially at weakly absorbing bands in the visible range of the spectrum. The third mode of CWV retrievals using 720 nm only, shows also a good retrieval along the whole dynamic range. Retrievals at low CWV values show less bias compared to the combination of 720 and 820 nm, while there is a stronger bias at high CWV values. The absolute average percent error is 19, 8.5, and 9% for retrievals using 820 and 940 nm, 720 and 820 nm, and 720 nm only, respectively. In Gao and Kaufman (2003), their CWV retrieval using MODIS showed a systematic bias relative to both a ground-based sunphotometers (AERONET) observations and a smaller bias to a ground-based, upward looking microwave radiometer (Gao and Kaufman, 2003). In the latter case, the error in CWV retrieval was less than 10%, which corroborates with the analysis shown here, except in the 820 and 940 nm combination, which is not ideal for a wide dynamic range of retrievals. Based on the analysis presented here, it is therefore recommended to use either 720 or 820 nm or both for the atmospheric correction of water vapor over oceans.

#### Application to HICO Imagery

To demonstrate the heritage AC extended to hyper-spectral, we present an application to Hyperspectral Imager for the Coastal Ocean (HICO) imagery, as detailed in Ibrahim et al. (2018). HICO (Table 1) is a hyper-spectral imaging radiometer that operated onboard the international space station (ISS) from 2009 to 2014, capturing over 10,000 scenes over the globe (Corson et al., 2008, 2010; Korwan et al., 2009; Lucke et al., 2011). HICO measured light with a spectral coverage from 353 nm to 1,080 nm with a 5.7 nm spectral resolution. It has a pointing capability in the cross-track direction. At the nadir looking direction, the spatial resolution is 90 m. HICO collected one scene per orbit of size 50 × 200 km that was scheduled weekly by the science team, with scenes mostly collected over coastal regions to derive products such as water clarity, benthic types, and bathymetry. HICO provided adequate radiometric performance to support ocean color applications in these coastal regions, where high concentrations of phytoplankton and suspended sediments result in high water reflectance in the visible regime (e.g., Dierssen et al., 2015b), but scenes collected over darker, open ocean regions suffer from the relatively low signal to noise ratio (SNR), especially in the green to NIR regime (Korwan et al., 2009; Lucke etal., 2011).

For HICO, an operationally viable algorithm for hyper-spectral ocean color retrieval has been implemented and assessed (Ibrahim et al., 2018), which follows the heritage approach used by NASA for all global ocean color sensors (Mobley et al., 2016). Water vapor correction, using the 720-nm spectral window, was a significant addition to that heritage process. The AC for HICO is completely automated, requiring no scene-specific operator intervention. As such, this work demonstrates the first operationally viable algorithm for hyper-spectral water reflectance retrieval, and can serve as a baseline AC for OCI on PACE. To minimize biases in the water reflectance retrievals due to uncertainty in HICO’s radiometric calibration or systematic algorithm errors, a system-level vicarious calibration was also developed and based on hyper-spectral *in-situ* measurements from MOBY (Franz et al., 2007).

The atmospheric correction algorithm and the gains derived from the vicarious calibration process were applied to the HICO observations to retrieve hyper-spectral remote sensing reflectance (*R*_*rs*_). As a verification of system performance, the approach was applied to all HICO scenes available over the MOBY site. In Figure 8, *R*_*rs*_ derived from HICO after the atmospheric correction process with and without applying the vicarious gain factors are compared to MOBY’s *in-situ R*_*rs*_ optically integrated to HICO’s spectral response function. Also shown is co-incident *R*_*rs*_ from MODIS onboard Aqua (MODIS-A), when available. It is clear that the *R*_*rs*_ match-ups from HICO are improved after applying the vicarious calibrations, showing a good agreement with both *in-situ* MOBY and MODIS-A retrievals. HICO’s *R*_*rs*_ also does not contain any features from the absorbing gases (i.e., negative reflectance at the 720-nm and 820-nm water vapor bands), including at the water vapor bands, emphasizing that the gaseous compensation process is performing well.

The hyper-spectral comparison of HICO and MOBY *R*_*rs*_ is very good. The improved NIR-band vicarious calibration, which determines the aerosol contribution for the atmospheric correction, reduces the bias in the visible spectrum. Overall, MODIS-A shows very good agreement in *R*_*rs*_ with MOBY, as expected, since the vicarious calibration was performed at the same site.

Figure 9 shows a true color image acquired by HICO in the Chesapeake Bay region at the east coast of the US, a highly complex and productive estuary with large anthropogenic influences on both the ocean and the atmosphere, and Figure 10 shows the retrieved *R*_*rs*_ at selected bands in the visible spectrum.

The retrieval of *R*_*rs*_ captures the large dynamic range due to the changes in the bio-optical properties of the water body. The *R*_*rs*_ images of HICO in the blue part of the spectrum exhibit image artifacts, such as stripping and reduced sensitivity. This is resultant of degraded sensor performance due to electronic smear and strong polarization sensitivity detailed in (Lucke et al., 2011). A detailed comparison between MODIS and HICO *R*_*rs*_ estimates for that scene is described in Ibrahim et al. (2018).

Figure 11 shows the hyper-spectral *R*_*rs*_ retrieved from HICO and the multi-spectral MODIS-A retrievals at three stations (STs) in the image from Chesapeake Bay. As in Figures 9, 10, Station 1 (ST1) is located in the York River, a highly turbid, highly productive region, Station 2 (ST2) is located at the mouth of the Chesapeake Bay, and Station 3 (ST3) is located just outside the bay in the Atlantic Ocean. The agreement between MODIS-A and HICO *R*_*rs*_ retrieval is very good for the three locations, indicating good consistency in algorithm performance regardless of the water type. Figure 11 also demonstrates the spectral features that HICO can resolve as compared to the multi-spectral MODIS-A.

### Recapitulation

The two-step heritage atmospheric correction algorithms have served the ocean community well, providing a reliable and efficient mechanism for the retrieval of water reflectance and derived marine bio-optical properties from multispectral satellite sensors. Within the framework of this heritage algorithm, and without employing deterministic or statistical methods, a prototype algorithm has been demonstrated for hyper-spectral atmospheric correction. This algorithm improves upon heritage by using measurements within and adjacent to water vapor absorption bands to derive column water vapor internally, without the need for ancillary inputs from reanalysis data. Exploration of this method points to the use of the 720 and 820 nm water vapor absorption bands to derive water vapor transmittance for use in the atmospheric correction algorithm, with the capability to produce column water-vapor concentration as a valuable additional product. Improvements will be required to accurately treat air-sea processes, such as wind-roughened and whitecap-prone seas, and conditions when the near infrared reflectance is enhanced due to surface blooms, vegetation or high turbidity.

## ALTERNATIVE ALGORITHMS

In order to discuss different optimization methods, including Bayesian optimization schemes (Gelman et al., 2013), that may be of relevance to the analysis of PACE hyper-spectral data we will introduce the relationship between the Likelihood function *P* (***y***|***x***_***w***_,***x***_***atm***_), the prior probabilities for the oceanic, *P*(***x***_***w***_), and atmospheric constituents, *P*(***x***_***atm***_), of interest and the posterior probability distribution, *P* (***x***_***w***_,***x***_***atm***_|***y***), viz.,

(4) $$P\left(x_{w},x_{atm}|y\right)\sim P\left(y|x_{w},x_{atm}\right)P\left(x_{w}\right)P\left(x_{atm}\right),$$

where we are assuming that the prior probabilities for the atmospheric state and the oceanic state are independent. In this formalism ***y*** is the vector of reflectances observed at the top of the atmosphere, ***x***_***w***_ is the vector of water reflectances and ***x***_***atm***_ is the vector of atmospheric properties (if any) that are estimated as part of the atmospheric correction process. Rodgers (2000) provided a comprehensive description of this approach for the atmospheric sciences. While not all optimization schemes explicitly make the link to the posterior probability distribution and the benefits of its use in obtaining an optimal estimate, most in fact use cost functions that are closely related to this form. The prior distribution is in some cases used purely to stabilize the search for ***x***_***w***_ and ***x***_***atm***_ (section Statistical Algorithms), but in more sophisticated approaches it is derived from existing data bases or is related to known functions of ***x***_***w***_ and ***x***_***atm***_ (section Multi-term Statistical Algorithm, GRASP Retrieval).

### Deterministic Algorithms

As noted above in section Algorithms to Retrieve Water Reflectance from Space, atmospheric correction is in principle an ill-posed problem since the range of possible atmospheric, surface and ocean states is not uniquely constrained even by hyper-spectral measurements. Nonetheless, by suitably constraining the problem and fitting all the observed TOA reflectances simultaneously, stable solutions that are valid under a variety of conditions relevant to atmospheric correction for PACE can be obtained. Examples of this type of one step approach are provided by Stamnes et al. (2003) in which a simple iterative scheme is used to provide a least squares estimate of chlorophyll concentration and aerosol optical thickness and Li et al. (2008) where a maximum a posteriori estimate of chlorophyll concentration, absorption by colored dissolved organic material, backscattering by suspended particulate matter, aerosol optical thickness and aerosol fine mode fraction is obtained. Similar optimization schemes are described in Land and Haigh (1997), Chomko and Gordon (1998), Kuchinke et al. (2009), Steinmetz et al. (2011), Shi et al. (2016). By representing the ocean and atmosphere with simplified parametric forms the estimation problem becomes stable, since its dimensionality has been reduced. As the approach presented by Li et al. (2008) is an optimal estimate in the sense described by Rodgers (2000) its extension to include the types of measurement in the UV and O_2_ A-band from a PACE OCI, that can be used to constrain aerosol absorption and vertical distribution, is natural. For example, in turbid coastal waters where absorbing aerosols are most likely to be an issue for atmospheric correction the ocean body reflectance in the deep blue and UV tends to be stable and low and can therefore be used primarily as a constraint on the atmosphere (Oo et al., 2008; He et al., 2012). The sensitivities of these radiances to aerosol absorption and vertical distribution, versus ocean body properties is incorporated into the retrieval scheme through its use of functional derivatives of the radiation field with respect to the parameters being retrieved (Jacobian matrices, see section Information Content Assessment) such that reflectances that are sensitive to a parameter play a greater role in its determination than those that are not sensitive to it.

More recently a particularly simple approach to representing the Rayleigh corrected atmosphere as a polynomial function of wavelength, referred to as POLYMER, was introduced (Steinmetz et al., 2011). Together with an ocean body reflectance parameterized with chlorophyll concentration and a non-covarying ocean body scattering term this representation was used to perform atmospheric correction in the presence of Sun glint. The application of this approach to PACE OCI data may be beneficial in terms of increased coverage by providing valid retrievals in the presence of Sun glint with a reflectance as bright as 20% and in the presence of semi-transparent clouds (Frouin et al., 2014). This is illustrated in Figure 12, which displays POLYMER-processed MERIS imagery acquired over the Northwest Atlantic (June 21, 2005). The satellite observation is contaminated by Sun glint and a variety of cloud systems, as evidenced in the RGB composite (Figure 12, left). Chlorophyll-a concentration is retrieved in the presence of the glint and thin clouds, and there is spatial continuity between cloud- or glint-contaminated areas and adjacent clear areas (Figure 12, right). A band of relatively high chlorophyll-a concentration, not detected in the RGB image, is revealed at the shelf break in the Bay of Biscay, a known phenomenon due to internal waves generated by the interaction of the surface tide with the steep topography (Pingree et al., 1986; Robinson, 2010).

However, it is important to recognize that all of the one-step methods described above have the same potential issue with regards to the atmospheric correction for PACE. While it is possible within this framework to provide water reflectance estimates that are relaxed from the assumed ocean body reflectance spectrum, e.g., Equation (10) of Steinmetz et al. (2011), it remains an open question as to how much and in what spectral domains the ocean body spectra assumed in the retrieval process will distort the estimated water reflectance spectra. Given the expected application of PACE OCI water reflectance to the identification of subtle absorption features (e.g., Gitelson et al., 2011) this clearly needs to be quantified as part of the use of any such algorithm. Tan et al. (2018) provide information about the representativeness of the 2-parameter water reflectance model now used in the POLYMER algorithm. The model, based on Park and Ruddick (2005), depends on chlorophyll concentration and a factor specifying the contribution of algal and non-algal particles to the backscattering coefficient. It was applied to 500 Case 1 and Case 2 water situations used in IOCCG (2006), and the parameter values giving the best fit against accurate Hydrolight simulations were determined following procedures described in Steinmetz et al. (2011). Agreement is generally good (about 10% RMS difference in the blue) between the two-parameter model results and Hydrolight values, i.e., much better than typical atmospheric correction errors), even in optically complex waters; many spectral details are correctly modeled in the 10-nm resolution reflectance spectrum. Significant differences exist in some cases, but having a more intricate model (i.e., using more parameters) might not guarantee convergence. The trade-off is between efficiency/robustness and accuracy.

### Statistical Algorithms

Neural networks (NNs) and other machine-learning techniques have seen considerable and diverse use in addressing the atmospheric correction problem (Schiller and Doerffer, 1999; Jamet et al., 2004, 2005; Brajard et al., 2006, 2008, 2012; Gross-Colzy et al., 2007a; Schroeder et al., 2007; Fan et al., 2017). This is because NNs and in particular multilayer feedforward networks with non-linear transfer functions provide a universal method to approximate arbitrary non-linear functions (Hornik, 1989). They can therefore be used either to solve the atmospheric correction problem directly by having observed reflectance and viewing geometry as input and water reflectance as output (Schroeder et al., 2007), or to model the radiative transfer equation (RTE) itself, where they replace the time-consuming solution of the RTE in an optimal estimation scheme (e.g., Brajard and Jamet references). In Gross-Colzy et al. (2007a) the TOA reflectance, after correction for gaseous absorption and molecular scattering, is decomposed into principal components (PCs), and the PCs sensitive to the water reflectance are combined to retrieve the PCs of the water reflectance. This allows a reconstruction, therefore estimation, of the water reflectance. Neural network methodology is used to approximate the non-linear functions that relate the useful PCs of the satellite reflectance to those of the water reflectance. Keeping only the water-sensitive PCs of the measured signal reduces the influence of the atmosphere and surface, making the non-linear mapping easier and accurate. The speed inherent in neural networks, once trained, means that they are a valuable tool for global processing of ocean color imagery and can be readily extended to the atmospheric correction of the hyper-spectral ocean color observations that will be provided by PACE OCI.

Figure 13 displays an example of water reflectance retrieval obtained with the PC-based algorithm (Gross-Colzy et al., 2007a) applied to Sentinel-2 MultiSpectral Instrument (MSI) imagery. The MSI scene was acquired over the Gironde estuary on October 21, 2016. The PC-based algorithm uses TOA reflectance in 11 spectral bands to retrieve water reflectance at 15 wavelengths including 412, 510, and 620 nm (not observed with MSI). This is possible since the TOA PCs can be mapped to the water PCs defined on a different base. The consequence (advantage) for PACE, is that it might not be necessary to observe in spectral regions strongly affected by gaseous absorption to retrieve water reflectance in those regions. As expected, the offshore waters are characterized by relatively high reflectance at 443 nm and low reflectance at 620 nm, and almost null reflectance at 865 nm, in contrast with the more productive and turbid waters of the estuary, which exhibit high reflectance at 620 nm (>0.25) and 865 nm (>0.10). In the estuary, the water reflectance spectra are similar in shape and magnitude to those reported by Doxaran et al. (2002), even the feature around 750 nm. The high-resolution (30 m) images also reveal a sharp contrast between the turbid estuarine waters and the clearer offshore waters, i.e., relatively little mixing, which would not be observed in coarse resolution (e.g., 1 km) imagery.

In the case where the NNs are used to provide a direct solution of the atmospheric correction problem it is not clear that this is applicable to the PACE OCI hyper-spectral data. As there is no relaxation to the observations based on the estimate of the atmospheric state, in this approach the NN estimate of water reflectance can only reproduce something close to what is in its training data set. A procedure should be applied to check whether the observations are compatible with the training data set. One of the main reasons for obtaining the PACE OCI hyper-spectral observations is that there is currently no such data set available to train a NN. In the case where NNs are used to model the RTE something similar to the optimal estimation approach described by Rodgers (2000) can be implemented since the gradients of the RTE that are needed can be obtained in a straightforward way directly from the NN that provides the forward simulation of the RTE (Bishop, 1995). While the forward model in such an optimal estimation scheme may contain assumed water reflectance spectrum, that assumption can be relaxed in the last step of the correction process (e.g., Equation 8 of Brajard et al., 2012). We note that if the Likelihood (errors in the observations) and prior probabilities (uncertainties in the atmosphere and ocean state vectors) are both Gaussian then the cost function that is being minimized in the NN approach is remarkably similar to −log[*P* (***x***_***w***_,***x***_***atm***_|***y***)], e.g., Equation (5) of (Brajard et al., 2012), and so the atmospheric correction will be similar to a Maximum A Posteriori estimate. However, the treatment of the priors is not based on data as one would expect in a completely Bayesian formulation, but is rather, a means to regularize the solution of the atmospheric correction problem. Nonetheless, based on their speed and applicability to cases where there are absorbing aerosols (Brajard et al., 2008) and to bright coastal waters (Brajard et al., 2012) NN atmospheric correction algorithms may well be appropriate for PACE OCI.

The identification of the cost function as akin to a Maximum A Posteriori estimate brings us to the subject of explicitly Bayesian AC approaches. While the use of a MAP estimate does not necessarily require detailed evaluation of the prior distributions, there are considerable benefits to doing so. As was noted above the priors can also be regarded as a way of regularizing the solution of the ill-posed atmospheric correction problem and ideally any such regularization should be based on independent observational, or theoretical evidence, rather than *ad hoc* estimates. Two recent papers introduce Bayesian methods for AC of ocean color (Frouin and Pelletier, 2015; Saulquin et al., 2016), although the solution approaches are completely different. Frouin and Pelletier (2015) construct a numerically efficient partitioning of the Rayleigh corrected, or “observed” spectral reflectance using a perfect binary tree. This partition is used in conjunction with inverse models estimated from simulated atmospheric and *in situ* ocean data to estimate the means and covariances of the water reflectance and the atmospheric state parameters. In addition a model probability is calculated, i.e., a quantitative measure of how well the TOA observations fit the forward model, allowing for the rejection of scenes for which the model is inappropriate. The method is numerically efficient and provides its own estimate of the mean and covariance of the water reflectance as an output. Since the atmospheric state is also estimated, a relaxation step similar to those identified above could be applied to the observed reflectance if that was required. However, the authors do point out that “using data instead of simulations for the water reflectance was deliberate, dictated by the fact that models do not take fully into account the natural correlations between the intervening optical parameters” and so the spectral variability of their water reflectance is not tied to a specific chlorophyll absorption spectrum. The numerical efficiency and fact that the spectral behavior is not tied to a specific absorption spectrum mean that this AC scheme should be applicable to PACE OCI. Saulquin et al. (2016) generate prior distributions that are Gaussian Mixture Models for the water reflectance and the atmospheric reflectance, using the MERMAID *in-situ* matchup database. They then use a gradient descent search to try and find a minimum of the Maximum A Posteriori cost function. Since the Maximum A Posteriori criterion may not be concave they use 25 random initializations to try and eliminate solutions that are captured in local minima. While this method has been successfully applied to AC in complex coastal waters, it is not clear that the existing method for finding the Maximum A Priori solution is sufficiently efficient for global applications.

Figure 14 presents an example of results obtained with the Bayesian methodology described in Frouin and Pelletier (2015). The scheme was applied to SeaWiFS imagery acquired over the Sea of Japan and northwest Pacific on April 7, 2001. In the Sea of Japan, the TOA reflectance at 865 nm (Figure 14, top left) is relatively high, due to dust from Northern China and pollution from the Korean Peninsula and Japan. This was confirmed by *in situ* aerosol measurements collected onboard R/V Brown during the ACE-Asia experiment and back trajectories (Kahn et al., 2004). In fact, most of the Sea of Japan was clear, but so hazy that, in the SeaWiFS Data Analysis System (SeaDAS) screening scheme applied here, many pixels were flagged as cloudy. The retrieved water reflectance at 555 and 412 nm, as well as associated uncertainties, and a quality index (*p*-value) are displayed in Figure 14 (top left and right and bottom). This ability to provide uncertainties on a pixel-by-pixel basis, i.e., to quantify the quality of the retrievals, is characteristic of the statistical technique. The water reflectance at 555 and 412 nm corresponds to chlorophyll concentrations typical of those observed in the region (Yamada et al., 2004). The uncertainties are higher in the vicinity of clouds and the coast, reaching 0.01 at 412 nm and 0.002 at 555 nm. In general, however, the values are much lower, e.g., below 0.004 at 412 nm. The quality index indicates good retrievals everywhere (*p*-value > 0.05), but values are lower where aerosol loading is higher (e.g., Sea of Japan). The marine reflectance retrieved by the SeaDAS AC algorithm (Mobley et al., 2016; see also section Heritage Atmospheric Correction Algorithm), reprocessing version R2010.0, exhibits more spatial noise (Figure 15, top left and bottom left); values at 412 nm are unrealistic (e.g., sometimes negative) in the Sea of Japan. Histograms of valid data (Figure 15, top center and bottom center) show for the statistical technique a frequency maximum shifted toward higher values by about 0.006 at 412 nm, with a narrower spread. Such shift does not exist at 555 nm and 670 nm. Variograms of valid data in a selected sub-area (150 × 150 pixels) located in the Japan Sea confirm less noisy marine reflectance imagery derived by the statistical technique (Figure 15, top right and bottom right), i.e., values are closer to zero as distance becomes small, and they show a reduced spatial variability over a 60-pixel distance. The results suggest that absorbing aerosols can be handled adequately using spectral information at wavelengths influenced by aerosol absorption. Accuracy is expected to improve by using observations at wavelengths in the UV (case of PACE OCI, where molecular scattering and, therefore, the coupling between aerosol absorption and molecular scattering are effective. Independent information about aerosol altitude and aerosol type, for example from the PACE polarimeter or a transport model, may help to constrain the inversion by restricting the domain of possible solutions.

### Multi-Term Statistical Algorithm, GRASP Retrieval

For more than a decade a multi-term statistical retrieval approach has been developed in Dubovik et al. (1995,^2006^, ^2011^), Dubovik and King (2000), and Dubovik (2004). The approach is based on the maximum likelihood methodology and therefore it is fundamentally close to the Bayesian optimization schemes given by Equation (4). However, the Multi-Term emphasizes the use of multiple a priori constraints and the Likelihood function *P* (***y***|***x***) is defined as follows:

(5) $$P\left(x|y,f_{1}(x),\ldots ,f_{N}(x)\right)=P(y|x)\prod_{i=1,\ldots ,N}P\left(f_{i}(x)|x\right)$$

Here, ***x*** denotes (***x***_***w***_, ***x***_***atm***_), ***y*** denotes measured multi-spectral, -angular, and -polarized reflectances, and ***f***_***i***_(***x***) denotes functions of ***x*** that are known a priori. If we have

(6) $$\prod_{i=1,\ldots ,N}P\left(f_{i}(x)|x\right)=P\left(f_{1}(x)|x\right)P\left(f_{2}(x)|x\right)=P\left(x_{w}|x\right)P\left(x_{atm}|x\right)$$

then Equation (5) becomes equivalent to Equation (4). At the same time, Equation (5) is evidently more general than Equation (4) since in many situations Equation (5) cannot be reduced to Equation (4). This aspect is rather important in many practical situations. Indeed, using direct assumptions about ***x***_***w***_ and ***x***_***atm***_ may lead to significant biases since it is very difficult and nearly impossible to find correctly a direct estimate of unknown parameters. For example, aerosol optical thickness in some areas may change by an order of magnitude and a single a priori climatological value cannot be used. In these regards, using some other constraints may be more adequate for PACE retrieval. For example, in remote sensing smoothness constraints are known to be efficient (e.g., Twomey, 1977). Namely, if one retrieves aerosol size distribution *n* (*r*), the limitation on the *k-th* derivative can be used as a priori constraint as $\partial^{k}(n(r))/\partial r^{k}$ ≈ *0*. In principle such smoothness constraint is less restrictive, since it does not apply any direct limitation on the magnitude of the retrieved parameters, but it only eliminates the solution when the retrieved function has unrealistically strong oscillations (see illustration by Twomey, 1977; Dubovik and King, 2000). Once a multi-term approach is employed, multiple a priori constraints can be used. For example, the Dubovik and King (2000) AERONET retrieval uses a priori constraints on aerosol size distribution, and spectral dependencies of real and imaginary parts of complex refractive index simultaneously. Dubovik et al. (2011) in retrievals from Polarization and Directionality of the Earth’s Reflectance (POLDER) onboard PARASOL derives simultaneously both aerosol and surface properties and for all spectrally dependent parameters of surface reflectance the smoothness constraints are also used. Moreover, Dubovik et al. (2011) proposed to define statistically optimized retrieval for a large group of POLDER/PARASOL observation pixels. For example, instead of Equation (5) for M pixels the Likelihood function can be defined as follows:

(7) $$P\left(x_{1},\ldots ,x_{M}|y_{1},\ldots ,y_{M},f_{1}\left(x_{1},\ldots ,x_{M}\right),\ldots ,f_{N}\left(x_{1},\ldots ,x_{M}\right)\right)=\;P\left(y_{1},\ldots ,y_{M}|x_{\text{1}},\ldots ,x_{M}\right)\prod_{i=1,\ldots ,N}P\left(f_{i}\left(x_{\text{1}},\ldots ,x_{M}\right)|x_{\text{1}},\ldots ,x_{M}\right)$$

where a priori constraints can be applied not only on the magnitude of parameter variability within each pixel, but also on their variability between the pixels. This allows the use of priori inter-pixel constraints to further improve the overall accuracy of the retrieval. For example, it is well known that land surface reflectance changes slowly over time, while spatial variability of aerosol parameters is also limited. Dubovik et al. (2011) showed that using both these constraints helps to achieve stable retrieval of aerosol properties even in such difficult situations as over bright land surfaces. For ocean surface application of time constraint is less critical, though applying some very mild constraints on time and horizontal variability of ocean surface parameters is also useful. Overall advantage of multi-term statistical retrieval is the possibility of retrieving extended set of unknowns. For example, Dubovik et al. (2011) retrieves more than 40 parameters for each POLDER/PARASOL pixel that includes aerosol size distribution, complex refractive index, fraction of spherical particles, aerosol height and spectrally dependent parameters of surface bidirectional reflectance and polarization distribution functions. It is also important to note that all a priori assumptions are general, i.e., no location-specific assumptions with exception of water fraction (i.e., water or land) that is assumed a priori using the pixel geographic coordinates. Moreover, a single initial guess for aerosol and surface parameters is used. Therefore, the results retrieved from POLDER/PARASOL observations are fully driven by the measured total and polarized reflectances (6 wavelengths, 16 viewing directions, and 3 polarization states, i.e., 192 data points for each inversion). In the future, some solid climatological information (e.g., about land surface) can be included too. All radiative transfer calculations including calculation of Jacobians are performed during the retrieval and the solution is sought in continuous solution space. Therefore, the errors of the retrievals also can be estimated using statistical approach. Since full radiative transfer calculations are used, the retrieval is significantly slower compare to conventional look-up-table algorithms. Nonetheless, during the last few years the computing routine was significantly optimized and the retrieval speed is acceptable now for processing large volumes of data and even for near real time retrieval. For example, the entire POLDER/PARASOL archive of 9 years was processed by GRASP (Generalized Retrieval of Aerosol and Surface Properties, see Dubovik et al., 2014) code. Figure 16 illustrates the global retrieval of aerosol optical thickness, angstrom exponent and single scattering albedo that allow identification of different aerosol types.

According to the Dubovik et al. (2011) concept, the surface reflectance for both land and ocean is retrieved together with the aerosol properties. Though, the retrieval of surface reflectance was not the focus of initial efforts, the robust results for aerosol retrieval may also generate accurate surface property retrievals. Figures 17–19 illustrate the first results for ocean surface retrieval. The reflective properties of the ocean surface are modeled analogously to earlier POLDER algorithm developments (Deuzé et al., 2001; Herman et al., 2005; Tanré et al., 2011). The Fresnel’s reflection on the agitated sea surface is taken into account using the Cox and Munk model (Cox and Munk, 1954). The above surface water reflectance is presumed to be nearly isotropic (Voss et al., 2007) and modeling shows that its polarization can be neglected (e.g., Chami et al., 2001; Chowdhary et al., 2006; Ota et al., 2010). This term and the whitecap reflection are taken into account by Lambertian unpolarized reflectances. The whitecap effective reflectance is driven by the wind speed at sea surface according to the Koepke (1984) model. The seawater reflectance at short wavelengths is not negligible and depends on the properties of oceanic waters. Thus, in the present model, the wind speed and the magnitude of seawater reflectance at each wavelength are retrieved together with aerosol properties. Figures 17–20 illustrate the retrieval of chlorophyll concentration, remote sensing reflectance, and wind speed and their comparison with MODIS results and ECMWF reanalysis.

The ocean surface reflectance model used here did not explicitly separate the contribution from whitecap, foam, and possible cloud contamination. Therefore, as initial approach for deriving water reflectance *ρ*_*w*_(λ) and estimating chlorophyll concentrations, the PARASOL/GRASP retrieval was tuned to the best agreement of MODIS chlorophyll retrieval for year 2008. Specifically, the chlorophyll fraction was fitted by the power law between logarithm of chlorophyll concentration and logarithms of ratio of water reflectance (440/565 and 490/565). The coefficients were estimated from best fit of MODIS chlorophyll concentrations using 1-year (2008) comparisons. The contribution of whitecap, foam, and possible cloud contamination was subtracted from retrieved values of surface albedo at shorter wavelengths using values at 870 and 1,020 nm. The coefficients for this subtraction were also estimated using the same comparisons with MODIS. Using the obtained empirical relationships, the water reflectances and chlorophyll concentrations were obtained from PARASOL/GRASP retrievals. As one can see from Figure 19, the obtained values are in rather good agreement MODIS results for both the global distribution and for the magnitudes of obtained values. Finally, Figure 20 displays the comparison of PARASOL/GRASP retrieved values of wind speed with the values provided by ECMWF ERA-Interim reanalysis data. Agreement is good between two data sets both for contour and location of specific geographical features and the correlation of the values. The agreement is especially encouraging taking into account that PARASOL/GRASP retrieval did not use any climatological or ancillary data. Thus the polarimetric multi-angular observations allow for robust retrieval of the wind speed. It should be noted that PARASOL/GRASP retrieval was performed for all non-cloudy ocean pixel including those in glint areas.

It should be noted that the above results shown for ocean surface properties are obtained from first version of PARASOL/GRASP processing without using quality control and any filtering. At present, dedicated efforts are planned for improving PARASOL/GRASP ocean surface retrieval. Specifically, it is planned detailed evaluation and refinement of the ocean surface model and other aspects of reflectance modeling and inversion. Also the core scientific code was realized in open source GRASP software (Dubovik et al., 2014, https://www.grasp-open.com/). This software benefits from the general flexibility of code concept and includes a number of convenient features for users. For example, different assumptions in forward model and inversion can be changed and tested. Also, the GRASP code is highly versatile. It can be applied to different types of satellite and ground-based observations. Therefore, there is high potential for different synergy retrievals using GRASP software or scientific approach.

### Other Optimal Estimation Approaches

Other recent work in atmospheric correction (Thompson et al., 2018) employed the Rodgers (2000) inversion framework more directly, adapting the Optimal Estimation (OE) methodology used by prior remote sounding missions like OCO-2, AIRS, and TES (Cressie, 2018). OE is based on a maximum likelihood method similar to the Dubovik et al. (2011) GRASP algorithm (see above), while it uses direct a priori estimates of the state vector. OE begins with an initial heuristic guess of the surface and atmosphere state, and then performs an iterative gradient ascent of the probability density until converging to a local maximum. At each iteration it calculates the probability gradient based on a local linearization under which the density can be characterized exclusively using multivariate Gaussians.

One emphasis of OE literature that does not appear in GRASP is the explicit treatment of unknown parameters in the models, i.e. parameters which affect the measurement but which are not directly retrieved. Specifically, the observation likelihood *P*(***y***|***x***) incorporates an instrument measurement noise model with a signal-dependent covariance, ***S***_*y*_, as well as any unknowns in the surface/atmosphere model which are not estimated directly. Such unknowns, represented by a covariance ***S***_*b*_, are treated as random variables. Jacobian matrices ***K***_*b*_ contain the partial derivatives of the measurement with respect to these unknowns, evaluated at the current estimated state. The total covariance of the observation system, ***S***_***ϵ***_, is therefore:

(8) $$S_{\epsilon }=S_{y}+K_{b}^{T}S_{b}K_{b}$$

Given a predictive forward model of the measurement ***f*** (***x***), and prior with mean ***x***_***a***_ and covariance ***S***_***a***_ the log probability density function leads naturally to a local cost function *χ*^***2***^(***x***) defined as:

(9) $$\chi^{2}(x)={\left(y-f(x)\right)}^{T}S_{\epsilon }^{-1}(y-f(x))+{\left(x-x_{a}\right)}^{T}S_{a}^{-1}\left(x-x_{a}\right)$$

At convergence, a similar linearization provides the posterior predictive covariance $\hat{S}$ of the estimated surface and atmosphere state.

(10) $$\hat{S}={\left(K^{T}S_{\epsilon }^{-1}K+S_{a}^{-1}\right)}^{-1}$$

The Jacobian matrices ***K*** hold partial derivatives of the observation with respect to the state vector. These have conventionally been calculated at the solution state, though Cressie (2018) points out that this is inconsistent with the delta rule derivation of $\hat{S}$. Consequently, it may be preferable to calculate ***K*** from a data-independent state such as the prior mean.

Posterior uncertainty distributions permit meaningful scientific hypothesis testing about surface properties by subsequent analyses. They can enable principled fusion of multiple measurements across space and time, under widely variable atmospheric conditions. This can help prevent regional bias—due, for example, to different water and atmospheric properties across latitudinal zones—from influencing global maps. More fundamentally, a full uncertainty accounting promotes a richer understanding of the observation system and its components. $\hat{S}$ is a purely local estimate, but it has been used successfully to create closed uncertainty accounting for validation experiments that fully explains the discrepancies between remote and field data. Thompson et al. (2018) demonstrated a closed OE uncertainty budget for AVIRIS-NG, a grating spectrometer with hundreds of bands across the Visible/Shortwave Interval. That demonstration used terrestrial spectra, with *in-situ* measurements of surface reflectance on six diverse validation targets. The approach generalizes naturally to the aquatic domain.

The most significant difference between terrestrial and aquatic environments is of course the surface. Ideally, a parameterization would be general and flexible enough to capture both environments—this would serve for coastal investigations, which monitor challenging Case 2 waters, as well as wetlands and vegetation with partially-inundated pixels. The Thompson et al. (2018) study complemented the multi-band GRASP research with flexible surface reflectance priors modeling capable of representing hundreds of channels. It used a collection of multivariate Gaussians, fit using a “universal” library of diverse spectra and designed to envelope of possible spectra that might be encountered. At runtime, Euclidean or Mahalanobis distance identified the component that was closest to the current state estimate, and the result is used as the surface prior for that iteration. Considerable shrinkage regularization (Theiler, 2012) broadened these priors still further, ensuring that the system could retrieve spectral shapes that were outside the span of library spectra. A designer would be free to craft the appropriate spectral intervals and magnitudes of this regularization depending on the application, which is useful when generalizing GRASP examples to the spectroscopic case. In extreme cases, the designer could leave priors totally unconstrained outside key spectral windows containing atmospheric information, such as NIR bands for water vapor and aerosols, in order to preserve precise channel wise relationships for retrieval of very subtle absorption shapes with high-resolution spectra (Thompson et al., 2018). In general, the use of structured surface priors can capture many of the same information used heuristically by typical aerosol retrieval methods, such as a flat infrared profile, while providing additional statistical rigor.

Multi-component surface models are easy to augment with phenomena related to Fresnel surface effects to permit water retrievals. Figure 21 shows the result of a transect across a scene imaged by NASA’s PRISM airborne spectrometer. This shows Santa Monica bay during an algal bloom event (Trinh et al., 2017), imaged from an altitude of 20 km that is subject to 95% of the atmosphere column (making it a good analog to orbital measurements). Here an additional free parameter of the surface model represents the magnitude of surface glint, which is assumed to be completely specular to produce the spectral profile of the direct beam. Surface models are produced using a large library of synthetic water reflectance spectra generated using a range of semi-analytical parameterizations (IOCCG, 2006), with additional regularization applied. Panels A and B show two examples of dark water spectra, with error bars indicating 95% posterior predictive uncertainties. Panels C and D show two examples of more productive and turbid water. Remote sensing reflectance *R*_*rs*_ was retrieved using the optimal estimation approach of Thompson et al. (2018). The model incorporates uncertainties due to measurement noise as well as the retrieval process itself, though they are of course local linearized estimates and may not capture all probability density maxima. The spectra show characteristic phytoplankton absorption features as well as solar-induced fluorescence at 685 nm, even though such features did not appear in the initial reflectance library. The experiment demonstrates the ability of this methodology to retrieve novel reflectance profiles having a wide range of different water reflectance shapes.

## ENHANCEMENTS USING MULTI-ANGULAR AND/OR POLARIMETRIC INFORMATION

For current instruments, the atmospheric correction, and subsequent retrieval of ocean properties, is a fundamentally underdetermined remote sensing problem. That is, the information contained in multi spectral observations cannot uniquely express the geophysical state. This leads to an inability, for example, to distinguish atmospheric from oceanic scattering, or to identify and account for aerosol absorption (IOCCG, 2010). While they can be mostly avoided in the open ocean, these issues are relevant in optically complex waters or in areas with non-maritime aerosols in the atmosphere.

One way to address underdetermined observations is to simply gather more information. The hyper-spectral capabilities of the OCI instrument on PACE are intended to better resolve in-water scattering and absorption. Aerosols, however, do not have strong spectral features, and their observation will remain underdetermined. The PACE science team has therefore investigated the capabilities provided by a Multi-Angle Polarimeter (MAP). MAP, as its name implies, is devoted to improving retrievals by also observing linear polarization at multiple viewing angles, in addition to multi-spectral or hyper-spectral radiometry.

The application of multi-angle polarimetry to satellite ocean color remote sensing is an emerging field of active research. In the previous section, the GRASP algorithm showed potential with POLDER data. In this section, we will describe theoretical studies exploring the information content of multi-angle polarimetry (section Information Content Assessment), followed by specific benefits of multi-angle observations (section Benefit of Multi-Angular Observations) and polarimetry (section Benefit of Polarimetric Observations) alone. Finally, we will discuss the benefits of combined multi-angle and polarimetric observations and provide examples of successful application of such instruments for ocean color remote sensing (section Benefit of Combining Multi-Angular and Polarimetric Observations). Background for the information content assessment described in section Information Content Assessment can also be found in Appendix A.

### Information Content Assessment

Information content assessment tools are used to explore the capability of a measurement system, real or theoretical, in order to find the optimal algorithm or instrument design. Such tools rely on simulated observations, and are thus subject to the realism of such simulations. When designed properly, these information content assessments can provide valuable input to measurement system design. We have used information content assessment tools to make the case that a MAP is indeed valuable for ocean color remote sensing, specifically in the ability to accurately perform atmospheric correction of a scene and successfully determine the water reflectance vector [***ρ***_***w***_], from the reflectance observed at the sensor.

Our methodology is based upon the Bayesian approach using Gaussian distributions as described in Rodgers (2000), and implemented for aerosol remote sensing by Knobelspiesse et al. (2012) and references therein. Theoretical details can be found in Appendix A, while the specific implementation of our study is described here.

For this study, we used a Doubling and Adding radiative transfer model developed at the NASA Goddard Institute for Space Studies [see Knobelspiesse et al. (2012) for more details]. This model uses a single parameter (Chlorophyll-a concentration) to define the optical properties of the ocean body (Chowdhary et al., 2012). While this is overly simplistic for hyper-spectral systems such as OCI, it is appropriate for the multispectral MAP, which is intended for atmospheric correction. We simulated a maritime aerosol defined by (Smirnov et al., 2002) at five optical thicknesses over an ocean with three values of chlorophyll-a concentration, *Chl-a*, for total of fifteen simulated scenes. Simulation details are described in Figure 22.

At the time this study was conducted, measurement characteristics for the MAP had yet to be finalized. We therefore chose to investigate a variety of prototypical MAP instrument described in Table 2. In each observed pixel, these instruments had either five or nine viewing angles, four visible channels or visible plus NIR spectral sensitivity, and were either radiometers or were sensitive to both the total and polarized radiometric state. All instruments had a combined (systematic and random) radiometric uncertainty of 3%, and polarimetric uncertainty of 0.005 (specified for the unitless DoLP). All instruments were compared to OCI as defined in the PACE Science Definition Team (SDT) report, containing 28 channels at UV, visible, NIR, and SWIR wavelengths (channels sensitive to gaseous absorption were omitted). Radiometric uncertainty was defined solely by threshold SNR requirements, implying perfect vicarious calibration and removal of systematic uncertainties. In our analysis, the MAP instrument is not vicariously calibrated, but OCI data are available to help the atmospheric correction (in other words, the theoretical measurement vector contains both MAP and OCI observations).

Figure 23 shows the Degrees of Freedom for Signal (DFS, see Appendix A for definition) for the cases with *Chl-a* specified at 0.3 mgm^−3^. All MAP instruments have significantly higher DFS than OCI alone. As expected, MAP “h” (blue dashed line), which has the most channels and viewing angles, has the largest DFS, while even non-polarimetrically sensitive multi-angle radiometers (a, b, e, f, red and magenta lines) have 1–3 more DFS than OCI. Some instruments with different characteristics produce similar results, notably instrument “d” (blue solid line, a five angle polarimeter with visible and NIR channels) and instrument “g” (green dashed line, a nine angle polarimeter with visible channels only). Based on DFS alone, the capability of those designs appears equivalent, so other factors (such as cost or engineering difficulty) could drive the selection of the appropriate design. However, *DFS* does not necessarily indicate atmospheric correction capability, rather, the ability to determine all parameters in state space. This is also demonstrated by the increase in DFS with simulation aerosol optical thickness. Obviously, increasing the aerosol load above the ocean does not improve the ability to determine ocean properties, but it does improve the ability to determine aerosol optical properties, and this is shown in the increase in DFS. Finally, it should be noted that the corresponding results for *Chl-a* = 0.03 and 2.0 mgm^−3^ are nearly identical, so the DFS is largely insensitive to ocean color, at least as it is resolved in our simple ocean model.

The goal of atmospheric correction is not the retrieval of all parameters in the state space, but instead determination of water reflectance, [***ρ***_***w***_]. For this reason, SDT report requirements are defined in terms of [***ρ***_***w***_] at the ocean surface. This is also why we have derived the byproduct error covariance matrix, ${\hat{S}}_{b},$ for [***ρ***_***w***_]. Diagonal elements of that matrix are the square of the expected retrieval uncertainty for each wavelength in [***ρ***_***w***_]. Figure 24 displays the ratio of this uncertainty to the predicted uncertainty of the OCI instrument alone. Thus a value of 1 indicates no improvement over OCI, while 0 is an infinite improvement. We chose this way of representing results in order to minimize the impact of imperfections in the radiative transfer model simulations, and because information content assessment is better at showing relative differences between measurement designs than absolute uncertainty values themselves.

Like in Figure 23, we find that MAP “h” (blue dashed line) offers the most improvement over OCI alone. Unlike in Figure 23, however, we find that “d” (blue solid line) and “g” (green dashled line) have different results. The nine angle visible wavelength MAP “g” is better than the five angle visible and NIR wavelength “d” at wavelengths less than 650 nm or so, and the reverse above 650 nm. However, [***ρ***_***w***_] wavelengths in the blue and green are most important for in water retrieval algorithms, so the “g” MAP is the preferable choice given this information.

Also unlike in Figure 23, we find that the ratio of MAP [***ρ***_***w***_] uncertainty to that of OCI varies with *Chl-a* and aerosol optical depth. The relative improvement from one MAP prototype to another is, however, largely maintained.

To summarize, we have found that the addition of a MAP to PACE offers distinct atmospheric correction capability compared to OCI alone. This is even the case for a multi-angle radiometer without polarization sensitivity. As in all sensitivity studies, these results are subject to the realism of the study design and require careful interpretation. However, we now have the means to assess and guide the development of a MAP, in terms of SDT required values, should it be funded as part of PACE.

### Benefit of Multi-Angular Observations

Multi-angle viewing provides information about aerosol properties in several ways, as discussed by Deschamps et al. (1994) in the context of POLDER and Diner et al. (1999, 2005) in the context of MISR, a multi-angle, non-polarimetrically sensitive radiometer that was launched into polar orbit in 1999 onboard NASA’s Terra spacecraft. MISR observes the Earth at nine viewing angles in the along-track direction, and four spectral channels centered at 447, 558, 672, and 886 nm. Thus, each pixel has 36 different angle or spectral channel combinations, providing significant information about the aerosol state. First, the aerosol signal increases with increasing viewing zenith angle as the optical path through the atmosphere becomes longer. Second, several scattering angles are sampled in the observations, and the dependence with scattering angle is sensitive to aerosol type and size. This information may help to determine the proper aerosol type in atmospheric correction schemes, or at least to restrict the selection to a reduced set of possible (theoretical or statistical) models.

Diner et al. (1999) showed that sulfate (accumulation mode) and urban soot aerosols cannot be distinguished by observing at nadir in the red and infrared, while the multi-angle MISR data (9 geometries) in this spectral region are able to identify that an incorrect aerosol model has been assumed in the retrieval of optical depth. Gordon (1997), following Wang and Gordon (1994), had earlier indicated that it is possible to atmospherically correct MISR imagery over water bodies using a single spectral band (near infrared), by comparing the angular distribution of TOA radiance with predictions using aerosol models and selecting the model that best matches the measurements.

Kaufman et al. (1997) hypothesized that aerosol single scattering albedo could be estimated by observing in and out of the Sun glint. In their technique, the measurements not contaminated by Sun glint are used to estimate aerosol scattering properties, whereas the direct transmittance measurements at the center of the glint benefit the estimate of aerosol extinction. Ottaviani et al. (2013) confirmed that additional aerosol information exists in measurements containing Sun glint, not only for single scattering albedo retrieval, but also for optical thickness and refractive index retrieval.

Recently, Limbacher and Kahn (2017) used multi angle MISR data in a matching algorithm to retrieve simultaneously chlorophyll concentration and aerosol properties (model and optical thickness). The difference between observed TOA reflectance (not only multi angle, but also spectral) and simulated values stored in a look-up table is minimized. By accounting explicitly for the water body contribution to the TOA signal, aerosol type retrievals are expected to be more accurate. Chlorophyll concentration estimates were in agreement with *in situ* measurements, although the number of such measurements was limited. Because of this, results were also compared against MODIS observations, and a similar agreement was found. Chlorophyll concentration estimates were in agreement with *in situ* measurements and MODIS estimates over a wide range of geophysical conditions. This algorithm constitutes an extension, using directional information, of a spectral optimization scheme proposed by Gordon et al. (1997) and Chomko and Gordon (1998) to improve water reflectance retrievals from SeaWiFS and MODIS imagery in the presence of absorbing aerosols.

An alternative approach is to use multi-angle measurements to determine water reflectance using an approach analogous to the Langley extrapolation technique used in the sun photometer community (Shaw, 1983). The method, proposed by Thieuleux (2002), constrains the spectral extrapolation of scattering properties observed in the near infrared by a value of the aerosol absorption effect obtained in the short-wavelength bands using the multi-angular acquisitions. A separate estimation of the aerosol absorption optical thickness and vertical distribution, i.e., the variables that govern the aerosol absorption effect, is not necessary. This constitutes a great advantage, since these variables are difficult to retrieve. First, the TOA reflectance is corrected for molecular and aerosol scattering using spectral bands in the near infrared and/or shortwave infrared, as in the classic (heritage) atmospheric correction scheme. Second, the residual signal in all viewing directions, *ρ*_*abs*_, composed of the aerosol absorption effect and the water signal (see section Improvements Using “Super-Sampling” in Selected Spectral Intervals, Equation 14) is related to an absorption predictor, i.e., a function representing the directional effect of an absorbing aerosol, namely the product of molecular reflectance, *ρ*_*r*_ and air mass, m*. By regressing *ρ*_*abs*_ vs. m*ρ_*r*_ (depends on geometry) one may get an estimate of the water reflectance *ρ*_*w*_ (the value at null m* gives the water reflectance). Since the relation is non-linear, in practice one may normalize *ρ*_*abs*_ by molecular transmittance *t*_*r*_. One may also use different absorption predictors, such as the absorption effect for a typical aerosol. Figure 25 illustrates the method for fine and coarse aerosols. The water reflectance (fixed at 0.02 in this case) is obtained by “extrapolating” the relation between *ρ*_*abs*_/*t*_*r*_ and *ρ*_*r*_m* to zero air mass.

An example of application to POLDER imagery is given in Figure 26. The determination of the aerosol model, therefore the correction of aerosol scattering effects, was accomplished according to IOCCG (2010), using measurements at 670 and 865 nm in all the viewing directions not contaminated by Sun glint. The multi-angular information was only used to compute an average spectral dependence of the aerosol scattering. After removing the scattering effects, the residual signal at 443 and 565 nm normalized by molecular transmittance was regressed against the product of molecular scattering and air mass, yielding an estimate of the water reflectance. In the regression, water reflectance was assumed to be isotropic. The aerosol optical thickness imagery at 865 nm is displayed in Figure 26 (top left). Relatively high values reaching 0.35 are obtained in the Eastern part of the Mediterranean basin. In this region, the standard atmospheric correction algorithm gives anomalously low marine reflectance at 443 nm (Figure 26, top right). The figure displays the average water reflectance over the viewing directions. After correction of the aerosol absorption effects, the water reflectance is higher in the dust-contaminated region (Figure 26, bottom left), which is consistent with the values in adjacent regions not affected by dust and with our knowledge of the bio-optical conditions in the Mediterranean Sea.

Some issues need to be examined to evaluate the feasibility of the multi-angular method and quantify its accuracy for the PACE mission in view of the capability of OCI and the multi-angle polarimeter. They include specifying the optimum set of viewing angles, investigating the influence of radiometric noise, defining requirements for relative multi-angle calibration, transferring the aerosol absorption information obtained by the polarimeter to OCI, and analyzing the influence of directionality in the water signal.

### Benefit of Polarimetric Observations

Ocean Ocean color remote sensing is based on TOA measurements of total radiance or reflectance, i.e., the first component (I) of the Stokes vector. A major issue with this approach is that atmospheric and surface effects dominate the signal measured at the UV to visible wavelengths of interest (Gordon, 1997; Zhai et al., 2017). To reduce these effects, one may consider exploiting the polarization properties of reflected sunlight. Because molecules, hydrosols, aerosols, and the air-sea interface polarize incident sunlight differentially, there may exist viewing geometries (scattering angles) for which the contribution of the water body to the polarized or unpolarized component of the TOA reflectance may be enhanced. For those geometries, correction of the perturbing effects becomes easier, leading to a more accurate retrieval of the water signal. Additionally, in some systems the method of measuring polarization can inherently be more accurate than measuring direct reflectance (Tyo et al., 2006; Dubovik et al., 2019).

He et al. (2014) and Liu et al. (2017) provide evidence of the advantages of including polarimetry for atmospheric correction over water bodies. They describe a method for retrieving normalized water-leaving radiance using parallel polarization radiance (*PPR* = *I* + *Q*), where *I* and *Q* are the first two components of the Stokes vector. Their results, from both simulations and application to POLDER data, demonstrate that using *PPR* enhances the ocean color retrieval in two important ways. First, it reduces the Sun glint at moderate to large solar zenith angles. Second, it boosts the water signal relative to the total radiance received by satellite at large view angles. These advantages are explained by the compensating effect between the total radiance and the polarization. For example, as view zenith angles increase, because of the increasing long path length through the atmosphere, the total radiance received by the satellite increases, causing the relative ocean color signal reaching the satellite to decrease. Meanwhile, the magnitude of *Q* increases with path length, but in the negative sense, which offsets the increase in *I*, and damps the increase in *PPR* with path length through the atmosphere.

Instead of using total reflectance or *PPR*, one may consider working with the unpolarized component of the TOA reflectance, as early suggested by Frouin et al. (1994). The rationale behind this approach is that scattering by atmospheric constituents and reflection by the surface, i.e., the processes causing the perturbing effects, polarizes incident sunlight. One expects, therefore, that they will affect less the unpolarized TOA signal than the total TOA signal. Of course, scattering by water molecules and hydrosols also polarizes incident sunlight, but due to refraction at the air-water interface the scattering angle in water is generally large for typical viewing geometries, i.e., the polarization rate is usually small. In other words, the signal from the water body is mainly unpolarized (see Fougnie et al., 1999) and, in view of the above, the contribution of the water signal to the TOA signal may be enhanced. For the approach to be suitable, the unpolarized signal from the water body must not only be strong and contribute more to the TOA signal, but also sufficiently sensitive to water constituents (phytoplankton, dissolved matter, sediments).

Figure 27 displays the ratio of water bidirectional reflectance just below the surface to TOA reflectance at 443 nm for typical geophysical conditions, i.e., maritime aerosols of optical thickness 0.1 at 550 nm, chlorophyll-a concentration of 0.1 mgm^−3^, and wind speed of 5 m s^−1^, and solar zenith angles of 30° and 60°. The simulations were performed with the OSOAA radiation transfer code (Chami et al., 2001). For some combinations of viewing and solar angles, especially when viewing zenith angle is less than 60°, the unpolarized reflectance ratio is larger compared with the total reflectance ratio, and the enhancement is by a factor of 2–3 in some situations. The enhancement is relatively small in the center of the Sun glint region when solar zenith angle is 30°, because scattering angles are not favorable to reducing sufficiently the molecular contribution to the unpolarized atmospheric reflectance, and the most favorable viewing angles are around those corresponding to specular reflection. The maximum enhancement is confined to more vertical geometries (i.e., viewing zenith angles less than 45°) when solar zenith angle is 60°.

Since aerosols tend to polarize incident sunlight less than molecules, the enhancement obtained using unpolarized reflectance is reduced when aerosol optical thickness is increased, all the more as multiple scattering decreases polarization. When the aerosol optical thickness is 1 (Figure 28, top), there is only a marginal gain in the relative contribution of the water body for some viewing directions. Depending on the surface conditions (e.g., wind speed), the Sun glint pattern may include a wider range of viewing angles, displacing and extending the favorable geometries (not shown here). When the chlorophyll-a concentration is increased from 0.1 to 10 mgm^−3^ (Figure 28, bottom), aerosol optical thickness remaining 0.1 at 550 nm, the best viewing directions correspond to in air scattering angles of about 90° in the forward direction. The enhancement is also large in the Sun glint region. As scattering angle increases, and especially in the backscattering directions, the water signal becomes more unpolarized, resulting in a small or no enhancement with unpolarized observations.

Figure 29 displays the TOA unpolarized reflectance ratio versus the TOA total reflectance ratio at 443 nm for the situations of Figures 27, 28. Reflectance ratio refers to the ratio of water reflectance just below the surface to TOA reflectance, expressed in percent. Only results for viewing zenith angles less than 60° (typical of satellite observations) are reported. As already mentioned, the unpolarized reflectance ratio is generally larger than the total reflectance ratio, for most viewing angles, except when aerosol optical thickness is large (i.e., 1 at 550 nm). In this case, the total reflectance ratio may be smaller by a few percent, which is practically not penalizing. Unpolarized observations in forward scattering directions, where polarization is effective, are most favorable to boost the contribution of the water signal. When chlorophyll concentration is high (i.e., 10 mgm^−3^), the unpolarized reflectance ratio is up to three times larger than the total reflectance ratio. In such productive waters, for which the water signal is usually small, using unpolarized observations would greatly facilitate atmospheric correction, which may yield to more accurate estimates of water constituents/properties.

Now when working with unpolarized reflectance, the sensitivity of the unpolarized signal backscattered by the water body (obtained after atmospheric correction) to water composition may differ from that of the total reflectance, due to changes in the polarization rate (Chami et al., 2001; Chowdhary et al., 2006; Zhai et al., 2017). This sensitivity should remain sufficient for the methodology to be effective, since the ultimate objective is not an accurate retrieval of the water signal, but an accurate retrieval of the properties that affect the water signal. Figure 30 displays, for typical solar and viewing angles, the sensitivity of the spectral ratio of water reflectance just below the surface at 443 and 550 nm, total or unpolarized, to chlorophyll-a concentration. This ratio is typically used in OC4x and OC3M algorithms. The atmospheric and surface conditions are those from Figure 27. Solar and viewing zenith angles are fixed at 30° and relative azimuth angle is 0°, 90°, or 180°. In the backscattering direction, as expected, the sensitivity to chlorophyll-a concentration remains unchanged whether using unpolarized or total water reflectance. In the forward scattering direction, the sensitivity is slightly higher at low chlorophyll-a concentration when using the unpolarized component (due to the decrease in polarization rate as chlorophyll concentration increases). This illustrates the potential of working with unpolarized reflectance, at least for chlorophyll-a concentration retrieval.

In summary, one may envision using the parallel polarization component (*PPR*) or the unpolarized component of TOA reflectance instead of the total reflectance (the usual way) to improve atmospheric correction of satellite optical imagery and retrieve properties (optical, biogeochemical) of the water body. The methodology only requires observations in a single direction, like with current ocean-color sensors. The advantages are that the water contribution to the measured signal may be substantially enhanced for a wide range of viewing angles and that Sun glint effects may be reduced. Also, in a classic atmospheric correction scheme, the polarization information in the near infrared (even in a single direction) would help to determine a proper aerosol model. A number of issues remain to be addressed; including radiometric calibration (polarization accuracy may degrade data quality) and bio-optical relations based on unpolarized component of water reflectance (need to be established, and experimental data are lacking). Furthermore, since molecular scattering strongly polarizes incident sunlight, one wonders whether the enhancement of the water signal would remain significant after removing the molecular signal (can be computed accurately, except in the presence of Sun glint) from the polarimetric measurements.

### Benefit of Combining Multi-Angular and Polarimetric Observations

The shortcoming of conventional atmospheric correction methods in regions with complex marine and atmospheric compositions suggests an opportunity for a multi-angle polarimeter to supplement OCI remote sensing reflectance (*R*_*rs*_) retrievals, particularly at short visible-UV wavelengths and in cases where the water surface in the NIR cannot be considered black, and will therefore provide significant risk reduction for meeting many PACE mission objectives. Indeed, measurements of the wavelength and angular dependence and polarization of the scattered radiance provide a determination of the aerosol physical properties, i.e., size distribution and refractive index (e.g., Hasekamp and Landgraf, 2005; Herman et al., 2005; Hasekamp et al., 2011). This information may be used to constrain the domain of possible aerosol types in a classic atmospheric correction scheme or, if sufficiently accurate, to directly compute the aerosol scattering effect. The sensitivity of polarized radiance to aerosol type has also the potential to improve inversion schemes that aim at retrieving simultaneously atmosphere and ocean properties.

Observational evidence is presented in Figure 31 that shows retrievals of *ρ*_*w*_*E*_*s*_/*π* (otherwise known as normalized water-leaving radiance, *L*_*wN*_***)*** obtained from AirMSPI measurements collected on February 6, 2013 over a SeaPRISM site off the coast of California. AirMSPI is a multi-angle polarimeter flying on the high altitude NASA ER-2 with 20 km of atmosphere between it and the ocean below. The SeaPRISM site on an offshore platform provides spectral *L*_*wn*_ measured just above the ocean surface. The retrieval was accomplished using an optimization-based multi-pixel retrieval algorithm (Dubovik et al., 2011). The forward radiative transfer calculations were performed using a Markov chain approach developed for a coupled atmosphere/surface system (Xu et al., 2011,^2012^). The water reflectance was modeled as a depolarizing Lambertian surface reflection model, plus a polarizing part modeled by the Cox-Munk model (Cox and Munk, 1954; Mishchenko et al., 1997). Atmosphere and surface properties were retrieved simultaneously, and surface reflectance was retrieved independently at each wavelength.

The comparisons between the AirMSPI retrievals with the SeaPRISM ground truth (Figure 31) show that *L*_*wN*_ is retrieved much more accurately with multi-angle and polarization capability than when a more limited retrieval is made with just one angle and no polarization. Current satellite retrievals mitigate random and systematic errors over the open ocean by invoking standardized models for ocean surface spectral reflectance and aerosol scattering. However, in more complex waters, or when aerosol absorption is present at short wavelengths, traditional retrieval assumptions break down. The left-hand figure does not prescribe either an aerosol model or any surface spectral constraints, indicating that multi-angle and polarimetric imagery provides the necessary additional information in the more general scenario. The right hand panel does not invoke any of the enhanced capabilities, including expanded wavelength range, expected of the PACE OCI, and therefore suggests a worse retrieval than could actually be obtained by the radiometer alone.

Demonstrations that show the advantages of a MAP for atmospheric correction as applied to AirMSPI and POLDER are extremely promising, but still do not demonstrate how polarimeter data can be used to assist atmospheric correction of hyper-spectral radiometer measurements in complex coastal environments. These environments are located where traditional atmospheric correction assumptions do not apply, and where inherent optical properties (IOPs) can vary much more significantly and in more complex manners than in open ocean waters. In addition, OCI retrievals at UV and shortwave visible wavelengths present new challenges to atmospheric correction due to sensitivities to aerosol height in an atmospheric signal dominated by Rayleigh scattering and the interplay between scattering and gaseous absorption. For these optically complex regions and at these spectral ranges, adding information from MAP becomes most important for atmospheric correction. Efforts to acquire and make use of the appropriate demonstration data are highly recommended.

These constraints can enter the atmospheric correction procedures at a variety of levels. (1) At the simplest, they can improve our overall understanding of aerosol properties and distribution of aerosol type, which can be incorporated into heritage atmospheric correction lookup table and algorithm updates. (2) MAP based aerosol retrieval could be performed prior to OCI atmospheric correction, and direct that algorithm to a particular aerosol type and/or provide the aerosol optical depth. (3) An atmospheric correction algorithm may be performed directly with MAP observations, and after spectral and geometric interpolation, applied to OCI. (4) Both MAP and OCI observations are combined as inputs to a joint inversion. Implementation of any of these approaches would require that we overcome multi-sensor data fusion issues such as instrumental calibration, differing spatial resolution and varied geometry.

Clouds are ubiquitous in remote sensing of the ocean, so the ability to properly screen and remove “cloud contaminated” pixels is an important component of successful atmospheric correction. Such techniques must be conservative, but a “cloud free” pixel still may contain sub-pixel clouds, or be influenced by cloud adjacency effects such as shadows or other “3D” phenomena. Combined multi-angular and polarimetric observations are capable of identifying such effects, and in some cases, minimize their impact. Breon and Goloub (1998) recognized that certain solar-view geometries observed by POLDER contained distinct cloud-bow features due to single scattering by liquid cloud drops, confirming earlier observations by Goloub et al. (1994) with airborne POLDER prototype observations. This was incorporated into POLDER cloud detection, cloud property retrieval, and cloud phase detection algorithms (Bréon and Colzy, 1999; Parol et al., 1999; Goloub et al., 2000). A later review of these capabilities by Parol et al. (2004) highlighted the utility of multi-angular polarimetric observations, but also found evidence of cloud contamination in an aggregate analysis of POLDER data. For these reasons, subsequent multi-angular polarimetric instruments were designed with the recognition that they would provide valuable data in both cloud and cloud free regions (e.g., Frouin et al., 2006; Mishchenko et al., 2007; Fougnie et al., 2018; Hasekamp et al., 2019). Additionally, polarimetric observations are recognized as being less sensitive to 3D effects (e.g., Davis and Marshak, 2010) but in certain geometries capable of detecting very optically thin cirrus clouds (Sun et al., 2014). The information contained in multi-angular polarimetric observations is highly sensitive to solar and observation geometry; it increases with additional measurements at angles containing the reflected cloud-bow. However, it can be large enough that simultaneous retrieval of cloud and aerosol property algorithms have been proposed for mixed cloud and cloud free pixels (Hasekamp, 2010), indicating the utility of such observations for properly cloud screened atmospheric correction.

## IMPROVEMENTS USING “SUPER-SAMPLING” IN SELECTED SPECTRAL INTERVALS

The PACE OCI will have the capability to measure TOA radiance in spectral bands 5nm wide with sampling at finer spectral steps (2.5,1.25, or 0.625 nm). This fine spectral sampling provides the opportunity to estimate Raman scattering by water bodies and improve retrievals of phytoplankton chlorophyll fluorescence and aerosol vertical distribution. These applications involve the development of original techniques that exploit spectral fluctuations in solar irradiance, the filling by inelastic fluorescence of oxygen absorption lines in the B-band, and the coupling between aerosol scattering and oxygen absorption in the A-band. They require or will benefit from the high spectral sampling.

### Estimation of Raman Scattering

Raman scattering occurs when an incident photon is scattered by water molecules excited into higher vibrational or rotational energy levels. The scattered photon has a frequency different from (usually lower than) that of the incident photon, i.e., the process is inelastic (kinetic energy of the incident photon is not conserved). This scattering by excitation is infrequent (1 in 10 million events), yet its contribution to diffuse water reflectance or “remote sensing” reflectance may reach 20% in the visible in clear oceanic areas (i.e., 40% of the world’s oceans), as shown by Marshall and Smith (1990) and others and illustrated in Figure 32.

The Raman signal, therefore, needs to be known to improve the retrieval of oceanic variables (e.g., chlorophyll concentration) from “remote sensing” reflectance. The fact that Raman scattering at a given wavelength is influenced by the water optical properties at shorter wavelengths provides a second motivation to observe the Raman signal. Are the Raman scattering measurements interpretable in terms of water optical properties, and how can they complement other approaches? Measurements in the near UV, for example, should be strongly affected by CDOM absorption, while in the blue-green by chlorophyll absorption.

Raman scattering at a given wavelength is proportional to some integral of the solar irradiance at shorter wavelengths and, unlike elastic scattering by water and air constituents, its spectrum does not exhibit fluctuations in spectral solar irradiance (Fraunhofer lines and others). The approach to estimate the Raman scattering signal is thus to observe the TOA radiance at sufficiently high spectral resolution in a small wavelength interval, and to separate the part of the radiance that correlates with the solar irradiance spectrum (the elastic scattering) from the part that does not (the Raman scattering).

At the air-water interface, the spectral water-leaving radiance, *L*_*w*_(λ), is the addition of an elastic scattering contribution, *L*_*w_elastic*_ (λ) proportional to the solar spectral irradiance, *E*_*s*_(λ), and a Raman scattering contribution, *L*_*w_raman*_ (λ), i.e., the integral of the excitation by the Sun at shorter wavelengths. At the TOA, the observed spectral radiance, *L*_*TOA*_ (λ), includes the signal due to atmospheric scattering and Fresnel reflection at the interface, *L*_*atm*_(λ), also proportional to *E*_*s*_(λ). Neglecting gaseous absorption and Sun glint effects for simplicity, *L*_*TOA*_ (λ) can be written:

(11) $$L_{TOA}(\lambda )=L_{atm}(\lambda )+L_{w_{-}\text{elastic}}(\lambda )t_{u}\;(\lambda ,\theta )\;\;+L_{w_{-}\text{raman}}(\lambda )t_{u}\;(\lambda ,\theta )$$

The ability to de-correlate the elastic and inelastic scattering contributions does depend on the presence of some absorption bands in the extraterrestrial solar spectrum over the selected spectral interval. Figure 33 shows the spectral solar irradiance, *E*_*s*_(λ), and the Raman “remote sensing” reflectance, *R*_*rs_raman*_(λ) (defined as *L*_*w_raman*_(λ)/*E*_*d*_ (λ) where *E*_*d*_ is the downward spectral solar irradiance at the surface, i.e., $E_{s}(\lambda )cos\left(\theta_{s}\right)t_{d}(\lambda ),$ in the range 350–550 nm at a 5nm resolution every 1.5 nm (approximating the spectral resolution and sampling expected from OCI). The Raman signal decreases with increasing chlorophyll-a concentration and CDOM absorption and exhibits fairly large spectral variations in some regions. For some intervals, namely 398.5–412.5 nm, 436.5–452.5, 473.5–484.5 nm, and 509.5–519.5 nm, however, the Raman signal is fairly constant with wavelength and the *E*_*s*_ variability with wavelength is sufficiently high to attempt a de-correlation of the Raman and elastic contributions to the TOA signal.

De-correlation of the two types of scattering can be accomplished by performing a linear fit of the observed spectral radiance (or equivalently reflectance) vs. the spectral solar irradiance in the intervals identified. In those relatively small spectral interval (10–15 nm wide), the spectral variation of the scattering properties, either elastic or Raman, can be assumed negligible, or, better, derived by a radiation transfer model with inputs of the optical aerosol and ocean properties as determined by the atmospheric correction algorithm. Note that the molecular atmospheric scattering contribution, which has a strong spectral dependence, can be subtracted from the TOA measurements. We have:

(12) $$P(\lambda )=P_{0}(\lambda )+L_{w_{-}elastic}(\lambda )+\langle L_{w_{-}raman}\rangle +F(\lambda )$$

with $P_{0}(\lambda )=\left[L_{atm}(\lambda )-L_{r}(\lambda )\right]/t_{u}\;(\lambda ,\theta )$ where *F*(λ) is a spectral factor that depends on the water optical properties (since again they may not be constant over the spectral interval considered). The ordinate at the origin of the best linear fit *P* vs. *E*_*s*_ or downward irradiance at the surface, *E*_*d*_, gives access to $\langle L_{w_{-}raman}\rangle .$

The approach and methodology are simple in principle, but to achieve useful accuracy a number of technical issues require attention: (1) Radiometric noise of the spectral measurements will result in an error on the water Raman scattering estimate that is amplified by the extrapolation of the linear fit to the ordinate axis; (2) Spectral resolution of the measurement will affect the variability of the spectral measurement as the solar spectrum is smoothed. A reduced variability of the solar spectrum will increase the error due to extrapolation; and (3) The sum of atmospheric and oceanic Raman scattering signals would be actually determined. Although the atmospheric Raman scattering is rather small (e.g., Vountas, 1998), it must be accounted for and subtracted from the water Raman scattering estimate; and (4) The Raman scattering signal can only be determined with good accuracy in some spectral intervals where the solar irradiance variability is large enough, namely around 390, 440, 475, and 515 nm, as indicated above. Interpolation/extrapolation to others wavelengths, definitively feasible, may not be straightforward since the Raman signal is not smooth spectrally.

Figure 34 illustrates the method feasibility. The various atmospheric functions were simulated in 5 nm bands shifted by 1.5 nm resolution for the 4 suitable spectral intervals using a successive-orders-of-scattering code. Aerosols were assumed of maritime type with an optical thickness of 0.2 at 550 nm, and wind speed was 5 m s^−1^. Sun and view zenith angles were 30° and relative azimuth angle was 90°. The water-leaving radiance (elastic and inelastic components) was simulated using HYDROLIGHT for Case 1 waters with a chlorophyll-a concentration, *Chl-a*, of 0.03 mgm^−3^. Typical CDOM absorption corresponding to biogenic particles was used. No noise was introduced on the TOA reflectance and atmospheric Raman scattering was neglected. Retrievals were obtained (1) assuming *F* = 0 (no spectral correction), and (2) assuming *F* was perfectly known.

When *F* = 0, large errors are obtained in the determination of $\langle L_{w_{-}raman}\rangle$, i.e., the methodology is not practically applicable. For example, in the 398.5–412.5 interval, the retrieved $\langle L_{w_{-}raman}\rangle$ value is 0.00028 instead of 0.00079 Wm^−2^nm^−1^sr^−1^ when *Chl-a* is 0.03 mgm^−3^. In the 436.5–452.5nm and 473.5–484.5nm intervals, the retrieval is completely erroneous. When using *F* computed assuming that *Chl-a* is known, the $\langle L_{w_{-}raman}\rangle$ estimates are very close to the prescribed values. This indicates that knowledge of *Chl-a* is essential to retrieve $\langle L_{w_{-}raman}\rangle$ accurately.

Now *Chl-a* can be obtained from standard band-ratio algorithms with a typical uncertainty of ±30%. The impact of such uncertainty on the $\langle L_{w_{-}raman}\rangle$ retrieval is still too large. For the case mentioned above, the $\langle L_{w_{-}raman}\rangle$ estimate would range from 0.00058 to 0.00165 Wm^−2^nm^−1^sr^−1^, which is not satisfactory. This means that *Chl-a* needs to be estimated more accurately for the methodology to work.

To estimate *Chl-a,* therefore *F*, more accurately, one may use the goodness of the linear fit (correlation coefficient). One expects that the linear regression will be more accurate when the spectral factor *F* corresponds to the actual *Chl-a* (see Figure 34). To increase sensitivity to *Chl-a*, especially in the presence of noise, the correlation coefficient may be computed for the 4 spectral intervals combined. This provides a practical way to determine the best *F* that corresponds to the observations. One could use a look-up table of *F(λ, Chl-a)* for each spectral interval to find the best *F* values that yield the best linear fit. As Raman scattering also depends on absorption by other variable constituents, the look-up table should include additional parameters (e.g., CDOM absorption coefficient). Additional theoretical and experimental work is needed, however, to demonstrate quantitatively the method’s applicability to OCI imagery.

### Estimation of Chlorophyll Fluorescence

Knowledge of the solar-induced chlorophyll fluorescence of natural waters is important to understanding the physiology of phytoplankton and investigating environmental influences on primary production and food web structure (e.g., Behrenfeld et al., 2009). Chlorophyll fluorescence can be remotely sensed from space, which has been routinely accomplished by instruments like MODIS and MERIS. The retrieval algorithms (Letelier and Abbott, 1996; Gower et al., 1999; Huot et al., 2005; Behrenfeld et al., 2009) involve the subtraction of a baseline representing the shape of the water reflectance spectrum without fluorescence. In waters containing sediments and yellow substances (Case 2 waters), determination of the baseline may not be accurate, yielding unacceptable uncertainties on the fluorescence height estimates, all the more as the chlorophyll concentration or the quantum yield of fluorescence are low. It is desirable to improve accuracy in these waters, for which fluorescence, unlike blue-to-green reflectance ratios, is a good measure of chlorophyll concentration.

To improve the estimate of chlorophyll fluorescence, one may use detailed spectral measurements in the oxygen B-band centered on 687 nm. The method exploits the fact that emitted fluorescence (observed at sea level) is excited at shorter wavelengths and is not affected by the oxygen absorption, contrary to the elastic water reflectance. As the absorption lines are partially filled due to inelastic fluorescence emission, the spectral change with respect to reflected solar radiance (i.e., to the elastic component) is sensitive to the fluorescence signal. By shifting the center wavelength in a spectral interval of 10–20 nm, one may be able to de-correlate the fluorescence signal from the elastic signal in the TOA measurements, therefore obtain a fluorescence estimate more independent from elastic scattering than the standard baseline technique. In other words, the fluorescence signal can be much more easily differentiated from the elastic signal when spectral measurements in the oxygen band are included.

Neglecting Sun glint, Raman scattering by water molecules, and assuming that in the spectral range of interest, i.e., 670–700 nm, gaseous absorption is only due to oxygen, the radiance *L*′_*TOA*_ measured from space, after correction for molecular scattering, can be expressed as:

(13) $${L\prime }_{TOA}=L_{a}T_{O2}\left(\theta_{s},\theta ,H_{a}\right)+L_{w_{-}elastic}\;t_{u}(\theta )T_{O2}\left(\theta_{s},\theta ,0\right)\;\;+L_{w_{-}fluo}\;t_{u}(\theta )T_{O2}(\theta ,0)$$

where *L*_*w_elastic*_ is the elastic water-leaving radiance, *L*_*w_fluo*_ is the radiance due to chlorophyll fluorescence, *T*_*O*2_ (*θ*_*s*_, *θ*, *H*_*a*_) is the oxygen transmittance associated with the path radiance, *T*_*O*2_ (*θ*_*s*_, *θ*, 0) is the oxygen transmittance associated with the elastic water reflectance (affected by absorption along the Sun-to-surface path), and *T*_*O*2_ (*θ*, 0) is the oxygen transmittance associated with the fluorescence signal (only along the surface-to-satellite path). The transmittances *T*_*O*2_ (*θ*_*s*_, *θ*, *H*_*a*_) and *T*_*O*2_ (*θ*_*s*_, *θ*, 0)are different, the first one in particular depends significantly on the vertical distribution of the aerosols (average altitude or scale height *H*_*a*_). In Equation (13), *L*_*w_fluo*_ can be expressed as the product of the radiance at the peak of fluorescence emission (i.e., 685 nm) and a known spectral function, *h* (see, e.g., Mobley, 1994), i.e., *L*_*w_fluo*_ = *hL*_*w_fluo*_(685).

The variable to retrieve is *L*_*w_fluo*_(685), the fluorescence line height. This can be accomplished using a spectral optimization scheme, in which *L*_*w_fluo*_(685) is varied to obtain the best fit between the modeled and actual (measured) TOA radiance *L*_*TOA*_′. The aerosol radiance *L*_*a*_ and the aerosol optical thickness and model (affect transmittances) can be determined from measurements in the near infrared and/or shortwave infrared using standard algorithms. The oxygen transmittance *TO*_2_ (*θ*_*s*_, *θ*, *H*_*a*_) can be computed from an estimate of *H*_*a*_ (e.g., from measurements in the oxygen absorption A-band centered on 763 nm, see Dubuisson et al., 2009 and section Estimation of Aerosol Vertical Profile) or assuming that the aerosols are located at an average aerosol altitude or at the surface. It is necessary to assume that the spectral shape of the elastic water-leaving radiance, *f* (λ) is rather constant, or known from a model as a function of chlorophyll concentration, *Chl-a*, and particulate backscattering.

To demonstrate the method feasibility, Case 1 and Case 2 waters containing 0.1, 1, 5, and 30 mgm^−3^ of chlorophyll-a were considered. Absorption by yellow substances and sediment concentration were null for the Case 1 waters and fixed at 0.5 m^−1^ (440 nm) and 2 gm^−3^, respectively, for the Case 2 waters. The fluorescence yield, which ranges from less than 0.01 to 0.10, was fixed at 0.05. Aerosols were of maritime type, with optical thickness of 0.2. The vertical profile of aerosol concentration was exponential with a scale height of 1 km. The TOA signal, including the coupling between oxygen absorption and aerosol scattering, was simulated with no noise using the quasi-single scattering approximation (a sufficient treatment to demonstrate feasibility). The HITRAN 2004 database was used to define spectroscopic parameters for oxygen absorption lines and compute oxygen transmittance. Absorption by water vapor was neglected. The simulations were performed for a single angular geometry, i.e., solar and viewing zenith angles of 45°, and a relative azimuth angle of 90°.

The spectral matching was accomplished on TOA reflectance, ${\rho \prime }^{TOA}=\pi^{\prime }L_{OA}/\left[E_{s}\cos\left(\theta_{s}\right)\right]$ rather than radiance, using a function minimization scheme (Nelder and Mead, 1965) over the range 660–720 nm. (Similar results were obtained when restricting the range to 670–700 nm.) The aerosol optical properties (optical thickness and reflectance) were assumed perfectly known. The aerosol scale height *H*_*a*_ was assumed to be 0.5 km. The elastic water reflectance, $\rho_{w_{-}elastic}=\pi L_{w_{-}elastic}/\left[E_{s}\cos\left(\theta_{s}\right)t_{d}\left(\theta_{s}\right)\right],$ was parameterized as $\rho_{w_{-}elastic}(685)\langle f(\lambda )\rangle \;\text{where}\;<f>$ is the average spectral function over all the water situations (*Chl-a* and water type). Thus the parameters used to adjust the modeled TOA reflectance, after correction for molecular scattering, were $\rho_{w_{-}elastic}(685)\;\text{and}\;\rho_{w_{-}fluo}(685)=\pi L_{w_{-}fluo}/\left[E_{s}\cos\left(\theta_{s}\right)t_{d}\left(\theta_{s}\right)\right].$ Figure 35 displays the results obtained for Case 1 and Case 2 waters containing 5 mgm^−3^ of chlorophyll-a. The retrieved and prescribed fluorescence signals agree well irrespective of water type, with differences <5% on *ρ*_*w_fluo*_ (685). The impact of a 0.5 km uncertainty on the 1km aerosol scale height (i.e., 50%) is small, but increased errors may occur when the difference between actual and specified scale height is larger. Accuracy remains similar for all the chlorophyll-a values in Case 1 waters, but decreases substantially with decreasing *Chl-a* in Case 2 waters (Figure 36).

Compared with the standard baseline technique using bands centered on 665, 682, and 705 nm, the spectral optimization in the spectral range of the oxygen B-band provides much better results (Figure 36). For Case 1 waters, the errors on estimated *ρ*_*w_fluo*_ (685) are decreased from 15 to 20% to about 5%. For Case 2 waters, the improvement is even more dramatic: Errors are decreased from about 200% to 22% when *Chl-a* = 0.1 mgm^−3^. The two techniques provide similar performance, however, in the presence of Case 2 when *Chl-a*> 5 mgm^−3^, i.e., within about ±20%. Note that when *Chl-a* is high in Case 1 waters the influence of chlorophyll absorption at 670 nm on the elastic reflectance makes it more difficult to determine the baseline, which degrades the retrieval accuracy of the standard technique (Figure 36, left). These findings, based on simulations, were obtained in controlled conditions, and the documented advantages may not be as important in natural conditions, which needs to be examined in future work.

The above results, even though they demonstrate the potential of using the oxygen B-band to improve chlorophyll fluorescence estimates, were obtained without noise on the TOA signal. The acceptable level of radiometric noise needs to be evaluated to achieve useful accuracy. The wavelength range for spectral matching may be optimized for maximum sensitivity to fluorescence height and minimum sensitivity to elastic water reflectance. In this respect, one may consider differential absorption using narrow and wide spectral bands (e.g., 5 and 30 nm, respectively) centered on 687 nm. Centering the two bands on the same wavelength would reduce the impact of the elastic water signal on the estimates, but sensitivity to fluorescence height may be reduced (5 nm may be too wide for the narrow band). Errors in the retrieval of the aerosol optical thickness, reflectance, and scale height (supposed to be determined separately), need also to be included to determine realistically the expected error budget.

### Estimation of Aerosol Vertical Profile

Information on the vertical aerosol profile is required for accurate atmospheric correction of satellite ocean-color imagery at short wavelengths (ultraviolet and blue) when absorbing aerosols are present (Gordon, 1997; Duforêt et al., 2007). This is due to the coupling between aerosol absorption and molecular scattering, which depends on the location of the aerosols in the vertical. Aerosol absorption reduces sun illumination and backscattering in the lower atmospheric layers, all the more as molecular scattering is large. Even if the standard atmospheric correction algorithm works well for “pure” scattering, an additional correction that depends on the aerosol vertical profile must be done at short wavelengths (see section Multiple Scattering).

The effect of aerosol absorption on the observed TOA reflectance, *ρ*_*TOA*_, can be written as (e.g., Torres et al., 2002):

(14) $$\rho_{abs}\approx -\left(1-\omega_{0a}\right)\tau_{a}m^{*}\left[\rho_{w}t+\rho_{r}\left(P_{s}-P_{a}\right)/P_{s}\right]$$

where *τ*_*a*_ is the aerosol optical thickness, *ω*_0*a*_ is the aerosol single scattering albedo, (1 − *ω*_0*a*_)*τ*_*a*_ is the absorption optical thickness, and *P*_*s*_ and *P*_*a*_ are surface and aerosol layer pressure levels. The observed signal, *ρ*_*TOA*_, is reduced due to aerosol absorption, all the more as aerosols are higher in the atmosphere, absorption optical thickness is larger, and air mass is larger. Note that marine reflectance exerts some influence on *ρ*_*abs*_.

This is illustrated in Figure 37, which displays *ρ*_*abs*_ as a function of wavelength (350 to 1,000 nm) and aerosol pressure level (300 to 1,000 hP_a_) for various aerosol models, i.e., continental (Con), urban (Urb), desert dust (DD), and biomass burning (BB). The calculations were made for a typical geometry, i.e., solar and viewing zenith angles of 30°, a relative azimuth angle of 90°, an aerosol optical thickness of 0.2 at 550 nm, and a null water reflectance. The absorption effect *ρ*_*abs*_ reaches −0.004 at 400 nm when aerosols are strongly absorbing (urban type) and located at 900 hP_a_ (Figure 37, left), but may be as large as 0.02 in magnitude at that wavelength when they are located higher in the vertical, i.e., at *P*_*a*_ < 400 hPa (Figure 37, right). For this relatively small aerosol optical thickness, *ρ*_*abs*_ remains significant in the presence of less absorbing aerosols (e.g., biomass burning and desert dust types), but would increase proportionally to optical thickness (see Equation 14). Neglecting *ρ*_*abs*_ in atmospheric correction algorithms, therefore, would yield unacceptable errors on water reflectance retrievals, well above the ±0.002 accuracy requirements for clear waters. Hence, taking into account aerosol vertical structure, a key variable controlling *ρ*_*abs*_, is needed when dealing with absorbing aerosols.

As indicated above, the effect of aerosol absorption, when aerosols are concentrated in a layer of average pressure *P*_*a*_, is proportional to the absorption optical thickness of the layer and to the molecular thickness below the layer, i.e., *P*_*s*_ − *P*_*a*_. In the more general case of an aerosol profile, *P*_*a*_ is replaced, in first approximation, by an equivalent mean pressure $\langle P_{a}\rangle ,$ defined by the integral over pressure of the aerosol optical thickness (from top of atmosphere) normalized by the total aerosol optical thickness. Thus $\langle P_{a}\rangle$ is the vertical structure parameter to introduce for a more effective atmospheric correction in the ultraviolet and blue (details about the vertical structure do not need to be known). This equivalent pressure can be derived from spectral measurements in the oxygen A-band around 762 nm.

The envisioned methodology to retrieve $\langle P_{a}\rangle$ exploits the coupling between aerosol scattering and oxygen absorption. The TOA radiance measured in the oxygen A-band depends on the altitude of the atmospheric scatterers, especially aerosols. The higher the aerosols, the larger the reflectance, since a number of photons are backscattered to space instead of being absorbed by oxygen in the lower layers. In addition, the coupling between aerosol scattering and oxygen absorption, which enhances absorption, is less effective in this case because there are fewer molecules. Dubuisson et al. (2009) showed that the ratio of measurements in a band strongly attenuated by oxygen absorption (i.e., within the A-band) and in a band minimally attenuated (e.g., outside the A-band) is sensitive to aerosol altitude. Using a relatively large single band in the oxygen A-band (case of POLDER and MERIS), retrieval accuracy was only satisfactory when aerosol optical thickness was > 0.3.

The oxygen absorption band is a feature about 5 nm wide with two absorption maxima separated by about 3 nm. The oxygen transmission averaged at the spectral resolution of 5 nm, exhibits a larger absorption peak that at a larger spectral resolution, and some details of the spectrum are still present with a sampling every 1.25 nm, even 2.5 nm. Such sampling, possible with the PACE OCI, is expected to help retrieve more accurate information about the vertical profile of aerosol scattering. In juxtaposed 5nm bands (or in larger spectral intervals) the spectral details would be missed, and the information about oxygen absorption would be too mixed with the out-of-band signal. An analysis of the effect of spectral resolution on the ability to use oxygen A-bands to retrieve aerosol layer height is given in Remer et al. (2019, this issue).

The inversion scheme is schematically the following. The objective is to retrieve the effective atmospheric pressure of the scattering aerosol profile, $\langle P_{a}\rangle$. Using a single scattering approximation, and neglecting surface reflectance, the TOA reflectance, *ρ*_*TOA*_, can be written as:

(15) $$\rho_{TOA}(\lambda )=\rho_{r,O2}(\lambda )+\rho_{a}(\lambda )T_{O2}\left(\lambda ,\langle P_{a}\rangle \right)$$

Where *ρ*_*r*,*O*_2__ is the molecular scattering computed in the presence of oxygen absorption, *ρ*_*a*_ is the aerosol scattering reflectance interpolated from the close measurements at, for example, 748 and 865 nm, i.e., out of the absorption band, and *T*_*O*2_ is the oxygen transmittance at the equivalent pressure $\langle P_{a}\rangle$. The various terms of Equation 15 depend on angular geometry (not shown for simplification). A spectral fit algorithm is then applied to retrieve $\langle P_{a}\rangle$. The spectral fit, i.e., the retrieval accuracy, will definitively be better with measurements at 5 nm resolution every 1.5 nm in the oxygen A-band than with fewer measurements in consecutive 5 nm bands. The issues to investigate include the impact of spectral resolution and radiometric noise on the retrieval accuracy of $\langle P_{a}\rangle$, and the influence of aerosol type and amount, and surface reflectance, which may not be null in the oxygen A-band. Note, incidentally, that the derived apparent pressure would constitute an excellent test to detect and filter out semi-transparent or small broken clouds in the absence of measurements in the thermal infrared. Effort is already underway and reported in Remer et al. (2019, this issue) to confirm that aerosol layer top pressure can be retrieved using oxygen A-band spectroscopy at the 5nm bands of OCI for aerosol optical thickness *τ*_*a*_ ≥ 0.3 for all surfaces and ≈0.1 for dark surfaces. In addition these studies show that *τ*_*a*_ may also be retrieved when angular information is added with a multi-angle polarimeter.

## SIGNIFICANT ISSUES

In a seminal paper about atmospheric correction in the EOS era, Gordon (1997) discussed approximations and issues that remained to be addressed in developing and operating the 2-step multi-scattering standard algorithm. The issues at the time covered an extensive range of topics; they included whitecaps, aerosol vertical structure, appropriateness of aerosol models, absorbing aerosols, stratospheric aerosols, Earths curvature, instrument polarization, surface roughness, in-water radiance distribution, diffuse transmittance, and radiometric calibration. Solutions (or approaches to solutions) were proposed to treat many of the issues, but some could not be handled properly, in particular those regarding aerosols. Alternative algorithms and the use of bi-directional and polarimetric information, however, have showed promise in difficult aerosol situations, either directly or indirectly (i.e., estimating aerosol optical properties separately), as demonstrated in sections Alternative Algorithms and Enhancements Using Multi-angular and/or Polarimetric Information. Some of the remaining issues are revisited here in view of new knowledge (whitecaps, Earths sphericity), as well as others either not considered in Gordon (1997) (horizontal heterogeneity) or that came to the forefront with the new PACE capabilities (complex and large atmospheric interference in the UV).

### Adjacency Effects

#### Evidence

Imagery of the Earths surface (land and ocean) obtained from space at optical wavelengths is degraded due to the atmosphere (e.g., Tanré et al., 1979). One of these effects is the adjacency effect (also known as the environment or blurring effect), defined as the change in the digital number of a pixel caused by atmospheric scattering of radiance that originates outside of the sensor field-of-view. Tanré et al. (1987), among others, have provided evidence for the adjacency effect in satellite imagery of land and ocean/lakes. They showed that over water the spectral dependence of the aerosol scattering determined in the red and near infrared (where the ocean can be considered black) might differ substantially from the actual one when the environment reflectance is high (case of green vegetation). Building on previous work (Tanré et al., 1981), they proposed a theoretical formalism to describe, therefore correct the adjacency effect. Diner and Martonchik (1985) also investigated theoretically the influence of atmospheric scattering on blurring of surface details in imagery acquired from space. They predicted that blurring of a spatial boundary would be enhanced in the presence of large particles (increased forward scattering).

The adjacency effect is generally ignored in standard atmospheric correction schemes and operational processing of satellite ocean-color imagery. Yet its impact on water reflectance retrieval and derived products (e.g., chlorophyll-a concentration) may be important, especially near land, sea ice, and clouds, i.e., where the environment reflectance is much different from the target reflectance (e.g., Santer and Schmechtig, 2000). These authors reported from theoretical calculations that for a typical contrast between land and ocean reflectance of 0.3 at 865 nm and 0.07 at 670 nm, a chlorophyll-a concentration of 2 mgm^−3^ is underestimated by 25% at a distance of 10 km from a linear coastline when the atmospheric visibility is 23 km and by 50% when the visibility is 8 km. The underestimation becomes larger as chlorophyll-a concentration increases, with retrieved values 5 times smaller than actual values at 10 mgm^−3^ when the visibility is 8 km.

An example of adjacency effect is given in Figure 38, which displays MERIS Level 1 imagery at 865 nm over the Zuydersee in the Netherlands. Aerosol optical thickness is about 0.3. Higher reflectance is generally observed near the coast (Figure 38, left), which is attributed to scattering into the field-of-view of photons reflected by the contiguous vegetated land, highly reflective at 865 nm. The TOA reflectance change along a section across the Zuydersee (indicated by a black line in Figure 38, left) reaches about 0.02 toward the coast over a 5–10 km distance (Figure 38, right).

Figure 39 displays MERIS Level 2 imagery, i.e., water reflectance at 560 nm, after standard atmospheric correction. A band of smaller marine reflectance is observed all around Corsica and Northern Sardinia (Figure 38, left), whereas anomalously high reflectance is observed for the waters surrounded by ice in the Beaufort Sea (Figure 25, right). This is likely the complex result of adjacency effects at 560 nm and at the near-infrared wavelengths used for the atmospheric correction. Depending on the environment (vegetation or ice), the coupling between surface reflection and atmospheric scattering may either decrease (Corsica/Sardinia case) or increase (Beaufort Sea case) the apparent reflectance with increasing wavelength, but this spectral dependence is not captured by determining the atmospheric signal from measurements in the near infrared and extrapolating to shorter wavelengths. Other examples have been observed along he coast of the Baltic Sea, and in Norwegian fjords.

#### Formulation

Over a homogeneous water body of reflectance *ρ*_*w*_, the TOA reflectance $\rho_{TOA}^{\text{hom}}$ can be expressed approximately as [e.g., Tanré et al., 1979; see Equation 3)]:

(16) $$\rho_{TOA}^{\text{hom}}\approx T_{g}\left(\theta_{s},\theta \right)\left[\rho_{atm}+\rho_{w}t_{d}\left(\theta_{s}\right)t_{u}(\theta )/\left(1-\rho_{w}S_{atm}\right)\right]$$

where *S*_*atm*_, is the spherical albedo of the atmosphere and transmittance due to aerosol and molecule scattering and aerosol absorption. The term (1 − *ρ*_*w*_*S*_*atm*_) accounts for multiple interactions between the atmosphere and the water body. Several approximations are used in Equation (16): (1) the surface is Lambertian; (2) Fresnel reflectance is neglected in the multiple interaction term, and (3) molecular absorption is decoupled from atmospheric scattering (i.e., *T*_*g*_ is simply a multiplicative factor).

Over a heterogeneous surface, one has to discriminate between the contribution of the target reflectance *ρ*_*w*_ viewed directly through the atmosphere and its background reflectance, *ρ*_*e*_ (Tanré et al., 1981, 1987; Santer and Schmechtig, 2000):

(17) $$\rho_{TOA}^{het}\approx T_{g}\left(\theta_{s},\theta \right)\{\rho_{atm}+t_{d}\left(\theta_{s}\right)[\rho_{w}\exp\left(-\tau_{atm}/\cos(\theta )\right)\;\;+\rho_{e}t_{u-dif}(\theta )]/\left(1-\rho_{e}S_{atm}\right)\}$$

where *τ*_*a*_ is the atmospheric optical thickness for scattering and $t_{u-dif}(\theta )$ is the diffuse component of total atmospheric transmittance due to atmospheric scattering, i.e., $t_{u}(\theta )=T_{u}(\theta )+t_{u-dif}(\theta ).$ The approximation used in this second formulation is that the target background is homogeneous, which is of course an idealized case. Nevertheless, Equation 17 shows that the retrieval of *ρ*_*w*_, using an atmospheric correction based on Equation 16 assuming a homogeneous scene, is affected by an error Δ*ρ*_*TOA*_ (i.e., the adjacency effect), which expression can be deduced from Equations 16 and 17:

(18) $$\Delta \rho_{TOA}=\rho_{TOA}^{het}-\rho_{TOA}^{\text{hom}}=\left(\rho_{e}-\rho_{w}\right)t_{u-dif}(\theta )T\left(\theta_{s}\right)$$

When the background is not uniform, which is the realistic case, the many individual contributions of every pixel that surrounds the target must be considered, and *ρ*_*e*_ becomes an average reflectance $\langle \rho_{e}\rangle :$

(19) $$\langle \rho_{e}\rangle =\iint f(x,y)\rho_{e}(x,y)\;dxdy$$

where *f* (*x*, *y*) is the atmospheric point spread function (PSF), which expresses the contribution to the measured reflectance of surface-leaving photons at horizontal coordinates (*x*, *y*) scattered in the field of view, and *ρ*_*e*_ (*x*, *y*) is the now spatially dependent environment reflectance. Given the properties of the atmospheric scattering, the PSF can be computed using a Monte Carlo code in backward mode, i.e., photons are injected in the direction of viewing and collected on the matrix of pixels at the surface (Reinersman and Carder, 1995). Eventually, the bi-directional reflectance of the pixelmust be accounted for in Equation 19 (Sun glint, vegetation), which means an integration of the probability of viewing the given pixel from different altitudes.

For observations at nadir, $\langle \rho_{e}\rangle$ becomes (Tanré et al., 1981):

(20) $$\langle \rho_{e}\rangle =\iint rg(r)\rho_{e}(r,\phi )drd\phi =\iint \left(\frac{dF}{dr}\right)\rho_{e}(r,\phi )drd\phi$$

*r* is the distance to the target, *rg*(*r*) is equal to *f* (*r*(*x*, *y*)), and *F* (*r*) is the integral of *rg* (*r*) between 0 and *r*, i.e., $F(r)=\int 2\pi r^{\prime }g\left(r^{\prime }\right)dr^{\prime }.$ The environment function *F* (*r*) gives the relative contribution to $\langle \rho_{e}\rangle$ of surface points within a radius *r* of the target pixel, and normalization is obtained by integrating from *r* = 0 to infinity, i.e., ∫2πF(r)dr=1.

Approximate analytical expressions of *F* (*r*) are given in Tanré et al. (1981) for molecular and aerosol scattering (typical continental aerosol model, scale height of 2 km), i.e.,

(21a) $$F_{m}(r)\approx 1-0.93\exp(-0.08r)-0.07\exp(-1.1r)$$

(21b) $$F_{a}(r)\approx 1-0.037\exp(-0.2r)-0.625\exp(-1.8r)$$

where *r* is expressed in km. In the case of aerosols characterized by a scale height *H*_*a*_ different from 2 km, Equation 21b is simply modified by multiplying *r* in the two exponentials by the weighting factor 2/*H*_*a*_. For a mixture of molecules and aerosols, *F*(*r*) becomes:

(22) $$F(r)\approx \left[t_{ur-dif}F_{m}(r)+t_{ua-dif}F_{a}(r)\right]/\left(t_{ur-dif}+t_{ua-dif}\right)$$

where *t*_*ur*–*dif*_ and *t*_*ua*–*dif*_ are the diffuse transmittances for molecular and aerosol scattering, respectively. Equations (20) to (22) allow the computation of $\langle \rho_{e}\rangle$ and therefore Δ_*ρTOA*_ for any pixel in a scene observed from space at nadir (or for nearly vertical sightings). They are helpful to understand the variability of the adjacency effect and to estimate its impact (i.e., expected errors) on the retrieval of the target reflectance. In the case of observations at slanted angles, the adjacency effect is more complicated to formulate analytically because of the lack of symmetry in azimuth [no simple insightful expression for $\langle \rho_{e}\rangle$].

#### Variability

The adjacency effect produced by molecular scattering and aerosol scattering is different and vary with *F* (*r*) and the reflectance of the background/environment. These processes act over disparate scales, i.e., about 12 km for molecular scattering and less than 1km for aerosol scattering (equations 21a and 21b). Pixels farther away have more influence, i.e., *F* (*r*) has a larger spread, when aerosols are located higher in altitude (1/*H*_*a*_ dependence in the exponentials of Equation 22). This also applies to cloudy situations. Furthermore, molecular scattering varies with wavelength and increases very rapidly in the blue, but *F*_*m*_ remains about the same with a good approximation. Aerosol scattering is less spectrally selective, but *F*_*a*_ given by Equation 21b is only typical. Changes in the aerosol physical properties, e.g., size distribution, would modify its single scattering phase function and the coefficients of the exponential decrease with distance *r*. Note that depending on solar and viewing geometry and Sun/sensor configuration with respect to the target and its environment, Fresnel reflection by the water surface (after scattering by the atmosphere) may contribute differently to the TOA signal (Santer and Schmechtig, 2000).

Figure 40 gives an example of adjacency effect when observing the ocean near the coast. The coastline is linear, the target is 5 km offshore, and the sensor is located above water or above land, viewing perpendicularly to the coastline at 30° zenith angle. The reflectance of land is 0.8 (typical value for snow) and isotropic, chlorophyll-a concentration is 1 mgm^−3^, aerosols are of maritime type with scale height of 2 km and optical thickness of 0.3 at 550 nm, and wind speed is 5 m s^−1^. The simulations are performed with a Monte Carlo Code operated in backward mode (Ramon et al., 2019). No assumptions are made regarding interactions between the surface and the atmosphere. TOA reflectance is higher due to the snow environment, especially at 380 nm, where atmospheric scattering is more effective (in first approximation the adjacency effect varies like $\tau_{a}\langle \rho_{e}\rangle .$ For a Sun at zenith, the reflectance increase is 0.05 at 380 nm, 0.03 at 500 nm, and 0.01 at 800 nm. If not taken into account, these values, by acting directly and indirectly (via atmospheric correction), would yield unacceptable errors on water reflectance estimates. The effect is larger when the sensor is over land due to reduced Fresnel reflection. The degree of polarization, on the other hand, is smaller, especially when the sensor is above land.

#### Correction

The TOA imagery can be corrected systematically for the adjacency effect at the Level 1b and produce a Level 1c, so that the processing of Level 2 products can be done by assuming that the surface is homogeneous (i.e., using the “large target” formalism of standard atmospheric correction schemes). This can be accomplished using a classic de-convolution algorithm, in which the de-convolution matrix is determined iteratively, since the reflectance of neighboring Level 1b pixels is affected by the adjacency effect (Reinersman and Carder, 1995; Vermote et al., 1997; Santer and Zagolski, 2008). A single iteration should normally be sufficient. In Vermote et al. (1997), for example, $\langle \rho_{e}\rangle$ is obtained using the surface reflectance and aerosol properties determined assuming no adjacency effects. This allows one to estimate the adjacency effect, correct the TOA signal accordingly, and then iterate. One issue for operational application, however, is processing time, i.e., de-convolution schemes need to be optimized. The simplest option, easy to implement, is to correct only the adjacency effects associated with molecular scattering. A second option, more accurate, is to correct also the effects associated with aerosol scattering. This can be done by (1) assuming background aerosols or using aerosols with average properties (eventually seasonally and regionally dependent), or (2) estimating the aerosol properties (optical thickness, type, and altitude). In the case of clouds, one can assume that they are located at the surface, or one can estimate their altitude.

Another way to deal with the adjacency effect is to develop atmospheric correction algorithms that minimize its influence. In the scheme proposed by Gross-Colzy et al. (2007a), only the principal components of the TOA signal most sensitive to the water signal are used to estimate the water reflectance, which reduces the impact of ρ_*TOA*_ in many situations. In the POLYMER algorithm (Steinmetz et al., 2011) the atmospheric signal is modeled as a polynomial function of wavelength with three terms, i.e., $C_{o}+C_{1}\lambda^{-1}+C_{2}\lambda^{-4}.$ The last term of the polynomial takes into account the coupling between molecular scattering and reflection by a gray surface (e.g., sea ice and clouds), which varies in λ^−4^, but the polynomial may also account for other types of surface/atmosphere coupling.

Correcting systematically Level 1b imagery for the adjacency effect, either explicitly or implicitly via proper atmospheric correction schemes, is definitely recommended. For coarse resolution sensors, it may be sufficient to account for the effect due to molecular scattering (the spread function associated with aerosols is effective over a relatively small distance, typically 1–2 km). As a result, the accuracy, quality, and daily coverage of ocean-color products should be improved substantially over water surfaces contiguous to land surfaces, sea-ice, and clouds, and in the open ocean where spatial variability may be large (e.g., upwelling regions). Otherwise, non-negligible errors would affect the retrieval of water reflectance, with a significant change in spatial structure and correlation scales (may alter the interpretation of mesoscale variability patterns, see Doney et al., 2003).

### Whitecaps

#### Importance

Whitecaps generated by breaking waves, generally composed of surface foam (large bubbles separated by a thin layer of water) and underwater bubbles, have a pronounced effect on the intensity and shape of visible light diffusively reflected by the ocean surface (e.g., Frouin et al., 1996; Moore et al., 2000; Stramski and Tegowski, 2001; Terrill et al., 2001; Zhang et al., 2002; Randolph et al., 2014). They may enhance dramatically water-leaving radiance on temporal scales of seconds to minutes (Frouin et al., 1996; Randolph et al., 2017) and affect large areas such as the windy Southern oceans. This is illustrated in Figures 41, 42, which display, respectively, pictures of seas with whitecaps and a time series of surface reflectance affected by whitecaps. In the presence of whitecaps, the dark ocean appears much brighter (Figure 41). At a local scale, the water reflectance, about 0.08 in the blue in the absence of whitecaps, is increased to 0.4–0.7 (i.e., by a factor of 5–11) depending on the breaking event, and variability is large over time scales of 5 to 10 s (Figure 42). The frequency and intensity of such breaking wave features over a 1-km pixel would determine the enhanced reflectance observed by the PACE sensor.

When present, the enhanced reflectance from whitecaps needs to be removed in order to produce estimates of water reflectance suitable for standard ocean-optics applications. From space, since whitecap reflectivity is high, even a small fraction of whitecaps within the instrument’s elementary field of view (i.e., within a pixel) may be problematic. The pixel reflectance is affected directly, but more importantly the spectral dependence of the whitecap reflectance skews the extrapolation to the visible of the aerosol signal determined in the near infrared, which may lead to water reflectance errors much larger (i.e., by an order of magnitude) than the requirements (Frouin et al., 1996).

Whitecap corrections are conducted as part of the overall “atmospheric correction” process, even though the signal does not originate in the atmosphere and can be considered a component of the water signal. The aim is to remove the signal due to actively breaking waves (Stage A), which includes surface foam (i.e., large bubbles separated by a thin layer of water) and underwater bubbles injected in the water column. It is not designed to remove enhancements due to the quiescent or mature phase of the whitecap (Stage B), when surface foam has dissipated, but bubbles are still present in the upper layer. In practice, much of the whitecap and the bubble plume signal is removed as part of the aerosol correction, since both foam and bubbles act to enhance near infrared reflectance and the current aerosol routines (heritage atmospheric correction) cannot distinguish between different sources of near infrared reflectance. However, some problems may arise due to the spectral dependence of whitecaps (mentioned above) and the coupling between molecular and aerosol scattering processes, which may differ significantly (since the amount of aerosols is artificially increased).

#### Current Approach to Whitecap Correction

The current approach is designed to remove the reflectance due to the actively breaking wave or bright white portion of the wave (Monahan, 1993) within each pixel. As part of that effort, an estimate of the fraction of the sea surface covered by whitecaps is generated for each pixel based on ancillary wind speed data. Whitecap fractions are commonly estimated using automated processing of digital photography of the sea surface (Brumer et al., 2017). However, comparisons of digital photography with radiometric approaches have shown that photographs capture primarily Stage A whitecaps and miss much of the Stage B bubble plumes that also enhance the surface reflectance. Many relations between whitecap fraction and wind speed have been developed over the last three decades for different ranges of wind speeds (Anguelova and Webster, 2006; Brumer et al., 2017). However, whitecap coverage can vary by several orders of magnitude at the same wind speed. At different locations in the world ocean, various environmental and meteorological factors act in concert but with different strengths and form a composite effect that either enhances or suppresses the effect of wind alone. These other factors include fetch and duration and the wind, water temperature, air temperature and stability of the lower atmosphere defined by the air/water temperature differential, salinity, current shear and long wave interaction, wave age, and the presence of surfactants such as organic films (reviewed in Scanlon and Ward, 2016). Hence, wind speed parameterizations should be viewed as “climatological” and cannot represent the instantaneous whitecap field required by remote sensing applications. In addition, other factors not related to wind speed could cause foam on water surfaces. High concentrations of the phytoplankton *Phaeocystis globosa,* for example, are associated with foam formation in coastal waters and on beaches (Armonies, 1989).

Satellite ocean color algorithms currently employ an expression for undeveloped seas based on wind speed at 10 m (*U*_10_) where *f*_*wc*_ = 8.75 × 10^−5^(*U*_10_ – 6.33)^−3^ (Stramska and Petelski, 2003), see Figure 43. This correction produces negative values below 6.33 m s^−1^ and is much higher than with other parameterizations at high wind speeds. To avoid overestimation, NASA implements a lower threshold of 6.33 m s^−1^ and an upper threshold equivalent to the whitecap fraction at 12 m s^−1^ in the operational code (Figure 43). Hence the highest fraction of the sea surface covered by whitecaps that is currently used is 1.6%. Although this range may cover the average conditions, whitecaps can occur at wind speeds as low as 3–4 m s^−1^ and the fractional whitecap coverage can approach 10% at wind speeds greater than 12 m s^−1^ (Brumer et al., 2017).

Measurement of the spectral reflectance of whitecaps is challenging due to the many types and stages of whitecaps, the rapid time scale on the order of seconds, determining the contribution of foam and background water, and potential contamination from reflectance of sun and skylight. Reflectance of whitecaps varies with the type of breaking wave (e.g., rolling breakers and plunging breakers) and the layers of foam produced. From historic data (Whitlock et al., 1982; Koepke, 1984), an age-averaged effective reflectance, *ρ*_*wc*_, of 22% is used in the NASA standard whitecap algorithm for visible wavelengths up to 550 nm. Frouin et al. (1996) studied the visible and near infrared reflectance of sea foam found in the turbulent surf zone. Sea foam reflectance was found to monotonically decrease into the near infrared wavelengths due to enhanced water absorption in these wavelengths. The spectral dependence was confirmed from aircraft measurements of whitecaps in the open ocean (Nicolas et al., 2001). From this work, a reduction factor is applied for red and near infrared wavelengths such that whitecap reflectance is 0.889, 0.760, and 0.645 at 670, 765, and 865 nm respectively and interpolated between these values. Whitecap reflectance can be approximated for wavelengths between 555 and 865 nm as: *ρ*_*wc*_ (λ > 555) = 0.22(−1.162 × 10^−3^λ + 1.653). For MODIS bands, this translates to whitecap reflectance of 22% from 412 to 555 nm and 19.3, 19.0, 17.2, and 14.1% at 667, 678, 748, and 869.5 nm, respectively.

Following the discussions above, the standard reflectance for actively breaking waves, *ρ*_*wc*_, is multiplied by the estimated fraction of the sea surface covered by whitecaps, *f*_*wc*_, to generate the within-pixel reflectance attributable to whitecaps. An important distinction must be made at this point. Whitecap reflectance is not treated as an “augmented” reflectance above the background reflectance, as is commonly interpreted. Because whitecaps are so bright, the signal is believed to dwarf the contributions from water reflectance and surface reflectance (skylight and glint). The estimate *ρ*_*wc*_ represents the total upwelling signal above the sea surface of a breaking wave, *E*_*u*_(0^+^), normalized to the downwelling irradiance incident on the sea surface, *E*_*d*_ (0^+^). Hence, the *ρ*_*wc*_ used in this context is considered invariant of the water reflectance and sky conditions. However, the actual enhancements due to whitecap reflectance can be influenced by many other factors including the intensity of the wave breaking and the concentrations of optical constituents in the water.

This whitecap contribution to reflectance is propagated through the atmosphere to estimate the enhancement $\rho_{wc}^{TOA}$ at the satellite. Assuming that whitecap reflectance is isotropic, we have:

(23) $$\rho_{wc}^{TOA}=f_{wc}\rho_{wc}t_{d}\left(\theta_{s}\right)t_{u}(\theta )$$

The at-sensor signal due to whitecaps $\left(\rho_{wc}^{TOA}\right)$ is then subtracted from the TOA reflectance measured at the satellite. This effectively accounts for the reflectance from the area of the sea surface that is covered by whitecaps. The whitecap-free area should also be weighted by (1-*f*_*wc*_) following Gordon (1997). Ignoring this weighting term is generally not important in the currently implementation of the whitecap correction where whitecap fractions are <2%, but area-weighting the whitecap-free sea surface would be important if higher whitecap fractions were modeled like in the Southern Ocean or for applications to high spatial resolution satellites.

#### Future Possibilities

Several issues must be considered going forward into the PACE era. The algorithm discussed above is a very crude and inaccurate representation of the impact of foam and bubbles on water-leaving radiance. Data collected to date show that wind speed can only roughly approximate the whitecap coverage in a climatological sense and cannot provide accurate real-time estimates of fractional whitecap coverage. The current wind speed thresholds used in the whitecap approach provide a minimal and largely inaccurate estimate of whitecap fraction that can be an order of magnitude lower or higher in the environment. Furthermore, use of a single reflectance value for all whitecaps does not represent the large amount of variability in the types of breaking waves and foam that can occur on the sea surface. Reflectance of whitecaps is variable based on the strength of the wave breaking and is not necessarily invariant of water reflectance signal. The bubbles plume or Stage B also serves to enhance reflectance substantially above background reflectance and this component is not explicitly treated in the model. Finally, the PACE mission is proposed to be hyper-spectral from the ultraviolet (350 nm) to near-infrared (885 nm) and have shortwave infrared bands at 940, 1,038, 1,250, 1,378, 1,615, 2,130, 2,260 nm. The current simplified spectral model of whitecaps will need modification to account for the spectral variability of whitecap reflectance in the near and shortwave infrared (see below).

The PACE mission aims to provide a better separation of atmosphere and ocean processes. Currently, much of the whitecap and bubble correction, particularly during high wind conditions, is inaccurately modeled as an aerosol. Whitecaps and bubble serve to enhance reflectance across the near and short wave infrared and cannot be easily separated using the current aerosol routines. Aerosol modeling assumes that water-leaving radiance at two near infrared wavebands are zero or can be estimated with sufficient accuracy using a bio-optical model. The presence of foam and bubbles is not explicit to this formulation and can lead to errors in retrievals when incorrectly treated as spectrally dependent backscattering from waterborne hydrosols or from airborne aerosols.

With more spectral information, the contribution of whitecaps may be measurable in the hyper-spectral signal and removed as part of the data processing scheme without relying on wind speed measurements and single reflectance values (see Dierssen, 2019, this issue). Measured reflectances of whitecaps and generated foam reveal identifiable spectral features from visible to short wave infrared (Figure 44). These new values are largely consistent with the diminishing reflectance in NIR wavebands measured by Frouin et al. (1996), but also reveal many features in the signal that cannot be dealt with as a simple linear decrease into the near infrared. Reflectance dips occur particularly at 750, 980, and 1,150 nm that have enhanced liquid water absorption, a result of multiple scattering in and around the bubbles and foam.

A potential approach for PACE and other missions is to identify spectral features associated with whitecaps that are unique from atmospheric and oceanic spectral properties. Water absorbs differently based on whether it is in vapor, liquid or solid form. As discussed above, unique peaks and dips occur in the near infrared portion of the reflectance spectrum that are associated with weak absorbing features of liquid water. The measured spectrum of intense foam and bubbles at the sea surface associated with the wake of a ship with a peak reflectance of 40% in visible wavelengths, As derived in Dierssen (2019, this issue), the general shape of this reflectance can be modeled from the logarithm of water absorption.

The overall shape of whitecap reflectance is directly related to liquid water absorption from visible through shortwave infrared. Some divergence at ultraviolet and violet wavelengths is apparent and likely due to colored substances such as absorption due to colored dissolved organic matter in these coastal waters. However, the overall shape can be used to assess the contribution of whitecaps into the near infrared and shortwave infrared. Parameterizations based on other physical considerations (e.g., vertical structure) should be contemplated to allow a direct estimation of the whitecap effect on water reflectance.

One approach would be to measure the depth of a liquid water absorption feature (e.g., 980 nm) to estimate the “effective” contribution of whitecaps to the total pixel reflectance. If we presume that the water reflectance does not contain this liquid water feature, then the line-depth of the water-absorption feature at 980 nm, for example, can be related to a linear mixing of unaffected sea surface with different intensities and areas with foam and bubble plumes. If the background water is highly scattering due to sediments or floating vegetation, for example, then this water absorption feature may also be present in the background signal and such water types would need to be identified prior to assessing the whitecap contribution (Dierssen, 2019, this issue). Candidate algorithms to model the whitecap factor, *A*_*wc*_, from the hyper-spectral radiance rather than the area-weighted whitecap fraction derived from wind speed are provided in Dierssen (2019, this issue). The whitecap factor is the fraction of a standard whitecap reflectance that accounts for enhancements in spectral reflectance of the sea surface above the background reflectance. Since *A*_*wc*_ is optically derived, it is better suited for atmospheric correction techniques because it specifically incorporates different levels of foam and bubbles associated with breaking waves and requires no implicit spatial scale.

In summary, decades of data have shown that wind speed models will not be able to predict the whitecap fraction with sufficient accuracy to use in daily remote sensing imagery. Use of other ancillary parameters such as fetch, air, and water temperature, and currents may have utility in further refining estimates of whitecap fraction. In the least, the Stramska and Petelski (2003) model should be replaced with a more recent parameterization that better fits both high and low wind speed conditions (e.g., Brumer et al., 2017). However, it is unlikely that the whitecap fraction and particularly the associated bubble plume will be predictable from wind speed data for any given image to within an order of magnitude (see scatter of data points in Figure 43 above).

We should continue to refine spectral models of foam and bubbles to understand their impact on upwelling irradiance at the pixel level. As part of that effort, more hyper-spectral datasets are critical that provide a means to estimate reflectance of whitecaps over time and space under a wide variety of water types and whitecap conditions. New methods that seek to use the measured spectral information in near infrared wavelengths to estimate the contribution of whitecaps directly will provide a means to separate whitecaps from aerosols and other conditions where near infrared reflectance is non-negligible. Preliminary results have been conducted at the sea surface and more research is warranted to model the transmission of hyper-spectral reflectance from whitecaps through the atmosphere. Moreover, uncertainties need to be assessed when aerosol products are derived from at-sensor radiance that is “contaminated” by whitecaps and bubble plumes. Continuing to propagate such errors impacts the selection of spectral aerosol model and magnitude of aerosols, as well as impacts the spectral shape and magnitude of retrieved water-leaving radiance and associated ocean color products. Until correction methods with sufficient accuracy are found and tested widely across ocean provinces, we recommend that PACE images collected under high wind conditions, which may contain significant whitecap coverage, be flagged such that users will recognize higher uncertainty in the water reflectance and aerosol retrievals.

Finally, breaking waves on the ocean surface are areas of significant importance to air-sea interaction. Whitecaps foster climate-relevant physical and chemical processes in the ocean, including the production of sea salt aerosols, mixing processes, and the exchange of gas (e.g., CO_2_, CH_4_, DMS, water vapor) and heat with the atmosphere (Monahan and Spillane, 1984; Woolf, 1997; Asher and Wanninkhof, 1998; Woolf et al., 2007). Wave breaking generates turbulent kinetic energy in the surface ocean, driving upper ocean mixing, and transferring energy to currents and longer waves (Cavaleri et al., 2007). Visible manifestations of breaking waves (i.e., foam and bubbles) are related to the energy injected into the surface ocean. Using imagery to assess whitecaps directly will open up new science directions.

### Earth’s Curvature

It was mentioned in section From Multi-Spectral to Hyper-Spectral Remote Sensing that assuming a plane-parallel atmosphere might introduce significant atmospheric correction errors for Sun zenith angles > 70°. This was also the conclusion of Ding and Gordon (1995), who suggested that for those angles a sufficient treatment would be to compute the molecular reflectance with a spherical-shell atmosphere RT code. It is important to emphasize, however, that the effect of Earth’s curvature is not only to increase intensity at grazing Sun zenith angles due to the smaller attenuation of the direct solar beam, but also to lower intensity at low Sun zenith angles due to a smaller illumination volume (e.g., Chowdhary et al., 2019, this issue; Ramon et al., 2019). This second effect is generally understated, but critical for accurate atmospheric correction. Figure 45 displays, as an example, Monte Carlo simulations of molecular reflectance, *ρ*_*r*_, and aerosol reflectance, ρ_*a*_, at 446, 558, 672, and 867 nm as a function of Sun zenith angle for a view zenith angle of 15° and a relative azimuth angle of 90°. Aerosols are of maritime type with optical thickness 0.1 at 550 nm. In the 0–75° Sun zenith angle range, differences between plane-parallel and spherical-shell calculations are relatively small (0.3–0.5%) for *ρ*_*r*_, and reach 1.5% for ρ_*a*_. However, since molecular scattering may be large and dominate the TOA signal, especially in the blue and UV, even a small fraction of 1% error in *ρ*_*r*_ would affect the water reflectance retrieval significantly (i.e., with resulting uncertainty above requirements). On the other hand, the impact of 1.5% differences in ρ_*a*_ is comparatively inconsequential for the aerosol loading considered (relatively small aerosol signal). But one expects non-negligible effects for higher view zenith angle and aerosol optical thickness. Thus Earth’s curvature should be taken into account in generating at least Rayleigh LUTs (it is also recommended to use spherical-shell RT code for aerosol LUTs), and this for all solar and viewing geometries, not only large Sun zenith angles.

### Multiple Scattering

Although the atmosphere is relatively thin optically compared with the water body, multiple scattering effects are not negligible in the atmosphere, especially at wavelengths for which scattering is effective (blue and UV). These effects are taken into account in atmospheric correction schemes, but their dependence on aerosol altitude is often neglected (case of the heritage algorithm in particular). It is generally assumed that aerosol altitude has a significant effect on the TOA signal only when aerosols are absorbing, which was discussed in section Estimation of Aerosol Vertical Profile. The coupling between scattering by molecules and aerosols, however, depends on their relative vertical distribution.

This is illustrated in Figure 46, which displays the coupling term of the aerosol reflectance at 443 nm for two very weakly absorbing models, i.e., Maritime with 98% humidity (M98) and Tropospheric with 70% humidity (T70). These models are part of the Shettle and Fenn (1979) suite used in early two-step atmospheric correction algorithms (e.g., Gordon and Wang, 1994; Gordon, 1997). Aerosol optical thickness is 0.1 at 865 nm, and aerosol concentration decreases exponentially with increasing altitude with scale height from 2 to 8 km. Sun zenith angle is 36.2°, and the results are presented as a function of viewing zenith angle (0 to 75°) in the principal plane of the Sun. Note that the coupling term can be negative since computed as the difference between the total atmospheric reflectance and the molecular component (i.e., with no aerosols) and the aerosol component (i.e., with no molecules) (Deschamps et al., 1983). It would always be positive if the molecular and aerosol components were computed for molecules only but in the presence of aerosols and for aerosols only but in the presence of molecules (Antoine and Morel, 1999). For some remote sensing geometries the effect of aerosol scale height reaches 0.002 (M98) and over 0.01 (T70) in amplitude, which in the case of T70 is one order of magnitude larger than the required accuracy on water reflectance. Thus it is important to include the effect of aerosol altitude when correcting the TOA signal for atmospheric effects, even when aerosols are little or not absorbing. This can be done using PACE observations at 5 nm resolution in the oxygen A-band, especially using the sub-5nm spectral sampling capabilities of the OCI, which allow an estimate of aerosol altitude or scale height (see section Estimation of Aerosol Vertical Profile).

### UV Observations

Observing in the UV is important to separate absorption by CDOM and phytoplankton pigments, to distinguish hydrosol types (e.g., sediments from organic particles) in optically complex waters, to allow the discrimination of functional, taxonomic, and harmful algal groups, and to improve atmospheric correction in the presence of absorbing aerosols. Hence measurements in the UV (from 350 nm) constitute a key PACE mission requirement. Retrieving accurately the water signal in the UV, however, presents a number of specific challenges that originate chiefly from the large optical thickness of the atmosphere. These challenges are emphasized in the following; see also Chowdhary et al. (2019, this issue) for a discussion of the UV regime in the context of RT modeling for atmospheric correction.

(1) The water reflectance contribution to the TOA signal is small in the UV, often much smaller than in the visible, especially when CDOM absorption is strong. This can be exploited in some situations (i.e., when the water signal is negligible) to constrain the aerosol signal estimated from NIR to SWIR wavelengths in a classic atmospheric correction scheme, but in general the water reflectance retrieval is more difficult. In addition, errors in estimating the spectral shape and magnitude of reflected sky radiance off of wind-roughened seas are enhanced in the UV. Currently this component is modeled as part of the Rayleigh LUTs for wind-roughened surfaces and is not validated with field data. A further complication is that aerosol and CDOM absorption may act similarly, i.e., their effects on the TOA signal may not be easy to separate. Also the steep decrease of solar irradiance due to absorption bands in this spectral range makes radiometric calibration difficult.

(2) Multiple scattering and coupling processes between molecular scattering and aerosol absorption are effective, and the resulting effect strongly depends on the relative vertical distribution of molecules and aerosols. This significantly complicates the atmospheric correction problem, for which knowledge of aerosol altitude becomes necessary. This will be possible with OCI (see section Estimation of Aerosol Vertical Profile and Remer et al., 2019, this issue). In one-step deterministic and statistical schemes, however, aerosol altitude can be variable, i.e., taken into account in determining the best solution.

(3) The influence of the environment, and in general spatial heterogeneities in the atmosphere, surface, and water body properties, is substantial (since depending on atmospheric transmittance, which itself depends exponentially on optical thickness see section Adjacency Effects and Figure 40, left), and the “large target” formalism may not yield acceptable water reflectance retrievals in coastal oceanic regions and water bodies surrounded by land (lakes, ponds, rivers).

(4) The spherical albedo of the atmosphere becomes large (e.g., 0.37 at 350 nm for a clear atmosphere) and neglecting the multiple interactions between the water body and the atmosphere may introduce errors of up to 2% (case of oligotrophic waters) on water reflectance estimates.

(5) Errors in the spectral dependence of aerosol scattering in the NIR and SWIR, by propagating through the standard algorithm, may translate into large errors in the UV (since further away than the visible). This and the complex RT processes in the UV make it difficult to achieve via “system” vicarious calibration (Franz et al., 2007) radiometric accuracy within a small fraction of 1%.

Figure 47 displays various components of the TOA signal and their relative importance for typical conditions, i.e., a WMO maritime atmosphere (aerosol optical thickness of 0.2 at 550 nm) over waters with chlorophyll-a concentration of 0.1 mgm^−3^ and CDOM absorption of 0.02 m^−1^ at 440 nm. Spectral range is 350–400 nm, Sun and view zenith angles are 30 and 15°, respectively, relative azimuth angle is 90°, aerosol scale height is 2km, and wind speed is 7m s^−1^. The water signal represents only 3 to 5% of the TOA signal and 34 to 42% of the TOA signal corrected for molecular scattering effects, with the lower values at 350 nm and the higher values at 400 nm. For waters with the same chlorophyll concentration but zero CDOM absorption, however, the contribution of the water signal would be larger, i.e., 8 to 12% and about 62%, respectively. For smaller aerosols, e.g., tropospheric type, the relative importance of the water signal would be reduced at shorter wavelengths. The small contribution of the water signal to the TOA signal stresses the importance of accurate radiometric calibration and modeling of molecular effects. In the case of Figure 47, an error as small as 0.1% on the TOA reflectance would already translate into a 3.3% error on the water reflectance at 350 nm.

Figure 48 displays the absorption effect of a continental aerosol with scale height of 2, 5, and 8 km on the TOA signal and the effect of CDOM absorption (0.01, 0.02, and 0.1 m^−1^ at 440 nm) on the water signal observed at TOA. The absorption effect is the difference between the atmospheric reflectance at TOA in the presence of aerosol absorption and without absorption (see section Estimation of Aerosol Vertical Profile and Equation 14). Chlorophyll-a concentration is 1 mgm^-3^. Spectral range, angular geometry, wind speed, and aerosol optical thickness are the same as in Figure 47. For the CDOM absorption effect, i.e., the difference between the water signal with and without CDOM absorption, aerosol scale height (slightly affects the atmospheric transmittance) is 2 km. The spectral dependence of the aerosol and CDOM absorption effects is comparable, making distinction of the two signals difficult in some one-step atmospheric correction schemes (e.g., spectral-matching). For example, 0.01 m^−1^ of CDOM absorption has nearly the same effect as aerosol absorption when scale height is 2km, i.e., about −0.002 over 50 nm.

## STRATEGY FOR ATMOSPHERIC CORRECTION

The standard, 2-step approach to atmospheric correction of satellite ocean-color imagery has been used operationally by space agencies since the CZCS was launched in 1978 to process data from major large-scale ocean color missions into water reflectance. The approach has proved to be robust, sufficiently accurate, and useful for many applications, scientific and societal (IOCCG, 2008). Its chief advantage over a variety of alternatives, as pointed out in sections Heritage Atmospheric Correction Algorithm and Alternative Algorithms, is that essentially minimal assumptions about water reflectance, the signal to be retrieved, are made. Algorithms have evolved and improved to account for new capabilities (e.g., observations in the shortwave infrared to deal with turbid waters) and take advantage of new knowledge (e.g., aerosol optical properties, whitecap reflectance), but have followed the same principle of using a spectral region where the water body is not or little reflecting to isolate the perturbing influence of the atmosphere and surface. In view of the success of this heritage approach, and the fact that it has been selected and applied systematically to correct imagery from most ocean color sensors, it is highly desirable to continue its usage in current and future processing lines, in particular for the PACE OCI. The adaptation to hyper-spectral OCI would address the challenges associated with observing in the ultraviolet and dealing with strong gaseous absorption bands; see section From Multi-Spectral to Hyper-Spectral Remote Sensing. Observing in the UV, even with hyper-spectral capability, continues to be an open question and area of active research, but does not negate the overall benefit of proceeding with a heritage 2-step retrieval for OCI. This continuity would maintain a certain level of consistency across sensors from different missions, allowing for a long-term record of water reflectance relatively free of algorithm-related discrepancies and biases. Furthermore, new methodologies may not be applicable to some sensors, complicating the generation of accurate long-term time series.

The heritage algorithm, however, has important limitations, as discussed in section Procedures. In particular it cannot handle properly situations of absorbing aerosols (i.e., dust, biomass burning and pollution particles), which are encountered over large oceanic regions and the coastal zone, reducing the spatiotemporal coverage of ocean color products, therefore their utility for scientific investigations and operational oceanography. It also does not work in Sun glint regions and in the presence of clouds, resulting in a typical 10–15% daily spatial coverage. To resolve these issues, one needs to move to further explore and implement alternative algorithms, deterministic or statistical, such as those described in section Alternative Algorithms. These algorithms exploit observations in the entire set of available spectral bands and eventually include, depending on the sensor, bidirectional and polarization information, either directly or indirectly (e.g., to constrain the set of possible aerosol models). So far only a few new algorithms have been checked thoroughly and applied systematically (i.e., at the scale of a satellite mission), notably the Steinmetz et al. (2011) spectral matching POLYMER algorithm and the Schroeder et al. (2007) neural network algorithm. Neural network atmospheric correction including sun glint correction was part of MERIS standard processing for Case 2 waters during the whole lifetime of the mission. Most of the advanced algorithms have been only tested on a few images, and they need to be further evaluated theoretically and experimentally and examined for robustness, reliability, and general applicability in an operational context. Their potential, however, as demonstrated in the examples presented in section Alternative Algorithms, is undoubtedly great to improve atmospheric correction in difficult situations (i.e., absorbing aerosols, turbid waters, Sun glint, whitecaps, land proximity, sea ice, thin clouds), offering the opportunity to generate more accurate ocean color products with wider coverage (i.e., less gaps) that will allow a better description of ocean properties (IOPs, concentrations) and biogeochemical phenomena.

In view of the above, a suitable strategy for atmospheric correction during the PACE era would be to continue performing atmospheric correction with the standard, 2-step heritage algorithm, adapted/adjusted as necessary, but at the same time applying the best advanced algorithm that fits the characteristics and capabilities of a given sensor, or a suitable combination of sensors. In the case of PACE, it is expected (by mission design) that OCI and polarimeters will be used synergistically. The selected methodology may be different, indeed, for a sensor that only measures spectrally total TOA radiance and a sensor that also measures directionally polarized TOA radiance. Whenever possible satellite data from previous missions would be reprocessed with the new algorithm. An elaborate flagging system, although not easy to define and validate, is necessary to warn the user about doubtful algorithm performance. The quality of the atmospheric correction processors, therefore selection of the best one, could be assessed following procedures developed by Müller et al. (2015a,b) for the ESA Ocean Color-Climate change Initiative (OC-CCI), which include in a straightforward fashion not only comparisons with *in situ* measurements, but also analysis of image quality and processor behavior along scan line (e.g., spatial and temporal homogeneity and consistency in water reflectance retrieval). Since individual advanced algorithms may lead to improved products (or may perform similarly) in some situations, or have advantageous features, their implementation in data analyzing systems such as SeaDAS would provide the user community with best options depending on the study or application. This strategy would generate two separate water reflectance products, which is not a problem as long as respective advantages and limitations are understood and uncertainties specified, preferentially on a pixel-by-pixel basis. Thus we would end up with a continuity product and an advanced product that fully exploits the mission capabilities, which for PACE will combine hyper-spectral radiometry and multi-angle polarimetry. The two products could be merged optimally, at the Level 3 highest resolution possible, i.e., native resolution, taking into account their uncertainties (bias and standard deviation). Various merging procedures exist (see IOCCG, 2007 for a review), and choosing the best procedure will depend critically on the representation of algorithm performance and uncertainties (expected to be better and smaller, respectively, for the advanced algorithm).

## CONCLUSIONS

Substantial progress in atmospheric correction of ocean-color imagery has been made since the proof-of-concept CZCS mission that demonstrated the feasibility of retrieving water reflectance from space and led to the first generation of operational ocean-color sensors (SeaWiFS, MODIS, MERIS, VIIRS, etc). Compared with CZCS, the new sensors have higher radiometric sensitivity and they measure in more and better-defined spectral bands in the visible. They also have spectral bands in the near infrared and (for some sensors) shortwave infrared, facilitating the removal of atmosphere and surface effects. This has generated a flurry of activities aimed at developing efficient atmospheric correction algorithms that exploit the new capabilities, which has contributed to a better understanding of the problem and resulted in significant improvements and new avenues.

Since the first attempts, the approach to atmospheric correction has essentially remained a 2-step process, in which the perturbing effects of the atmosphere and surface are determined in spectral bands where the signal from the water body can be considered negligible (i.e., in the near infrared and/or shortwave infrared) and propagated or extrapolated to the shorter spectral bands. Definite improvements to algorithms based on this approach have been made, notably in the specification of the aerosol models (based on AERONET measurements), the correction of gaseous absorption (e.g., nitrous oxide influence in the blue), and the processing of imagery over optically complex waters, where the water-leaving signal in the near infrared (or even SWIR) is not negligible and must be estimated through iterative bio-optical modeling. A number of approximations and issues have been identified and studied, such as whitecaps, Sun glint, aerosol vertical structure, polarization, sea surface roughness, Earth curvature, in-water radiance angular distribution, diffuse transmittance, absorbing atmospheric gases, absorbing aerosols, stratospheric aerosols, optically thin clouds, highly turbid waters, and adjacency effects. For some of these issues, satisfactory solutions have been proposed and implemented operationally, but not for others (e.g., absorbing aerosols, whitecaps, adjacency effects). In the absence of satisfactory solutions for a given observing condition, the operational approach has been to allow the atmospheric correction to fail, or to flag the water reflectance retrievals as suspect quality. This has resulted in large data gaps in some regions, preventing the efficient utilization of ocean color products.

Alternative approaches, deterministic and statistical, which aim at inverting the top-of-atmosphere signal (i.e., retrieving the water reflectance) in a single step, have been developed with varied degrees of success. The information in all available spectral bands is generally used, which is advantageous in some situations (e.g., absorbing aerosols), but the main drawback is that water reflectance, i.e., the signal to retrieve, is often constrained by a model (which may not represent well the environmental conditions of a given observation). Some algorithms have been able to handle situations of Sun glint and thin clouds, increasing significantly the daily coverage of ocean color products. All the proposed methods, however, have been based on multi-spectral observations, the information generally available; few attempts have been made to exploit the polarized and/or directional properties of reflected sunlight (available for some sensors, e.g., POLDER and MISR), but the potential of these properties to improve atmospheric correction (e.g., to enhance the contribution of water reflectance, to deal effectively with Sun glint, and to determine absorption effects) has been recognized and, to some extent, demonstrated. The improvements revealed in the selected examples, however, need to be confirmed (e.g., by analyzing other situations) before drawing final conclusions.

The PACE mission, which will carry into space a spectrometer measuring at 5 nm resolution in the UV to NIR with additional spectral bands in the SWIR and two multi-angle polarimeters, has great potential for improving estimates of water reflectance in the post-EOS era. The improved instrument capabilities offer opportunities, but retrieving a continuous water reflectance spectrum at 5 nm resolution in the UV to NIR, a chief mission objective, poses new challenges. First, measurements in the UV, where molecular scattering is effective (and may dominate the signal), are strongly affected by the vertical structure of atmospheric scatterers, and adjacency effects are large. Second, correcting measurements in spectral regions where gaseous absorption is strong may be difficult when absorbers are located in lower atmospheric levels (e.g., water vapor) due to coupling between scattering and absorption. Observing in the UV to SWIR, however, is adapted to the problem since observations in the UV are sensitive to absorbing aerosols, those in the SWIR to coarse aerosols (and they allow a better separation of the atmospheric/surface signal), and those in the oxygen-A band (763 nm) to aerosol altitude, a parameter controlling aerosol absorption effects. This is especially useful for inversion schemes that incorporate all the available information to retrieve water reflectance. Polarized and multi-angular measurements, sensitive to aerosol type, increase the information content in the inversion process and, therefore, are expected to yield more accurate retrievals, but the possibilities and improvements have yet to be fully investigated.

During the PACE era one may envision an approach to atmospheric correction that is based primarily on the heritage 2-step algorithm, taking into account and extending past accomplishments, yet exploiting as much as possible new capabilities (observations in the UV and in oxygen-A band) and multi-angular polarimetry (e.g., to constrain the aerosol model ensemble or estimate directly aerosol absorption effects). It should be clear that polarized and directional measurements are essential here, due to the inherent limitations of the heritage algorithm, in particular its inability to handle absorbing aerosols, which may affect large oceanic areas. This strategy, aimed at ensuring continuity with previous ocean-color missions, should be complemented by the development and implementation of more sophisticated inversion schemes (deterministic, Bayesian) that, by the nature of their construction, their robustness, and their generalization capabilities, have the undeniable potential to become the new standard in the near future.

Despite the progress made and the major improvements in atmospheric correction expected during PACE, important gaps/issues still remain to be filled/tackled. They include (1) improving the accuracy of whitecap corrections, for which coverage and optical properties are highly variable and difficult to model, (2) accounting for Earth curvature effects in atmospheric radiative transfer, which may be significant even at small zenith angles, (3) correcting for adjacency effects (influential near land, sea ice, and clouds) and cloud shadows, (4) accounting for the coupling between scattering and absorption (especially important in the UV where the molecular scattering signal is large), (5) modeling accurately water reflectance, including polarization (too little is known about the polarization properties of hydrosols), and (6) acquiring a sufficiently representative data set of water reflectance in the UV to SWIR to describe the variety of water bodies (such prior information is needed in Bayesian approaches). Dedicated efforts, experimental as well as theoretical, are in order to gather the necessary information and resolve inadequacies. Ideas and solutions exist and have been put forward to address the unsolved issues, thanks especially to the new capabilities provided by PACE, which will mark the beginning of a new era of accurate ocean-color radiometry from space.

## Supplementary Material

1

## Figures and Tables

**TABLE 1 |:** HICO sensor and data characteristics.

Platform	International Space Station (ISS)
Operation lifetime	2009–2014
Orbit repeat time/period	3 days/90 min
Scene size (km)	50 × 200
Pixel size (m)	~100
Wavelength (nm)	353–1080 (128 bands)
Spectral resolution (nm)	5.7
Spectral FWHM (nm)	10 (≤745 nm), 20 (>745 nm)
Sensor type	Offner Spectrometer
Signal-to-noise ratio (SNR)	>200:1 assuming 5% surface albedo
Polarization sensitivity	<5%

**TABLE 2 |:** Prototype MAP characteristics.

	# view angles	Radiometer	Polarimeter	Channels (nm)	Radiometric uncertainty	Polarimetric uncertainty
a	5: −50°, −25°,0°,25°,50°	Yes	No	Visible: 443, 555, 670, 865	3%	-
b	5: −50°, −25°, 0°, 25°, 50°	Yes	No	Visible + NIR: 443, 555, 670, 865, 1640, 2250	3%	-
c	5: −50°, —25°,0°,25°,50°	Yes	Yes	Visible: 443, 555, 670, 865	3%	0.5%
d	5: −50°, —25°,0°,25°,50°	Yes	Yes	Visible + NIR: 443, 555, 670, 865, 1640, 2250	3%	0.5%
e	9: −70.5°, −60°, −45.6°, −26.1°, 0°, 26.1° 45.6°, 60°, 70.5°	Yes	No	Visible: 443, 555, 670, 865	3%	-
f	9: −70.5°, −60°, −45.6°, −26.1°, 0°, 26.1° 45.6°, 60°, 70.5°	Yes	No	Visible + NIR: 443, 555, 670, 865, 1640, 2250	3%	-
g	9: −70.5°, −60°, −45.6°, −26.1°, 0°, 26.1° 45.6°, 60°, 70.5°	Yes	Yes	Visible: 443, 555, 670, 865	3%	0.5%
h	9: −70.5°, −60°, −45.6°, −26.1°, 0°, 26.1° 45.6°, 60°, 70.5°	Yes	Yes	Visible + NIR: 443, 555, 670, 865, 1640, 2250	3%	0.5%

## APPENDIX A: METHODOLOGY FOR INFORMATION CONTENT ASSESSMENT

The information content assessment described in section Information Content Assessment uses a Bayesian approach with Gaussian distributions as described in Rodgers (2000), and implemented for aerosol remote sensing by Knobelspiesse et al. (2012) and references therein. This method estimates retrieval uncertainty given an observational configuration and uncertainty with the Equation:

$${\hat{S}}^{-1}=K^{T}S_{\epsilon }K+S_{a}^{-1}$$

where $\hat{S}$ is the retrieval error covariance matrix, ***S***_***ϵ***_ is the observation error covariance matrix, ***S***_***a***_ is the *a priori* error covariance matrix, ***K*** is the Jacobian (forward model sensitivity) matrix, ^**T**^ denotes the transpose, and ^**−1**^ denotes the inverse. The observation error covariance matrix represents measurement uncertainty and is square, with the dimension of the number of measurements [m x m], made with each observation. The retrieval error covariance matrix, $\hat{S}$, has a similar structure, but represents the uncertainty in parameters retrieved from the data and has the dimension of the number of retrieved parameters [n × n]. Essentially, it is the projection of observational uncertainties into state (parameter) space. The Jacobian matrix, ***K***, expresses the sensitivity of the atmosphere/ocean radiative transfer model to changes in the parameters to be retrieved, and has the dimension [m x n]. Radiative transfer simulations, indicated by the function ***F*** (***x***) = ***y*** (where ***x*** is a vector atmospheric and surface optical parameters, often called the state space, and ***y*** is the measurement vector), are used to estimate the Jacobian matrix

$$K_{i,j}(x)=\frac{\partial F_{i}(x)}{\partial x_{j}}\approx \frac{F_{i}\left(x^{\prime }\right)-F_{i}(x)}{x_{j}^{\prime }-x_{j}}$$

where the partial derivative of the radiative transfer model for the simulated set of parameters, ***x***, is computed for each observation, ***i***, and each parameter, ***j***. We approximate the Jacobian using the forward difference technique, where the radiative transfer model is rerun with a perturbed parameter **x′**_***j***_, and the forward model is assumed linear over that perturbation.

This method provides the means to relate measurement characteristics to expected retrieval success. Instrument capability is defined by the contents of the measurement vector, ***y***, and the measurement uncertainty defined in **S**_***ϵ***_. The *a priori* error covariance matrix, ***S***_***a***_, describes what we know about state parameters ***x*** prior to a measurement. The Jacobian, ***K***, is the product of a radiative transfer model and thus computationally expensive to generate. It is, however, crucial since it links parameter to measurement space. In practice, we generate a Jacobian matrix for a very large measurement vector, encompassing all possible measurement spectral sensitivities, geometries, and polarimetric states, and then make a subset corresponding to the instrument system in question. Since the atmosphere/ocean system is highly nonlinear, we also need to repeat this analysis for a variety of states, and express the results as an aggregate of many states. For example, we might expect the ability to determine ocean properties decreases as the amount of aerosol loading increases, so we must test with a variety of aerosol amounts.

The averaging kernel matrix, ***A*** (also known as the model resolution matrix), is a useful reformulation that is an identity matrix for perfect retrieval ability. It is calculated as:

$$A={\left[K^{T}S_{\epsilon }K+S_{a}^{-1}\right]}^{-1}K^{T}S_{\epsilon }K$$

where a null matrix indicates no ability to determine the state parameters beyond what is already known from the *a priori* matrix. The Degrees of Freedom for Signal is a scalar, representation of measurement system capability, calculated as *DFS=trace(**A**)*, and has the property *0* ≤ *DFS* ≤ *n*. We use *DFS* to compactly show system capability for many simulated states.

We used a forward model that defines the measurement state with ocean and atmospheric physical and optical properties. However, PACE SDT requirements were expressed as the water reflectance [***ρ***_***w***_], which is instead a byproduct of the model. We must therefore project the results $\hat{S}$ into this space. To do so, we first determine the Jacobian matrix, ***J***, for water reflectance in the same manner as ***K***:

$$J_{l,j}(x)=\frac{\partial M_{l}(x)}{\partial x_{j}}\approx \frac{M_{l}\left(x^{\prime }\right)-M_{l}(x)}{x_{j}^{\prime }-x_{j}}$$

where λ corresponds to each wavelength in [***ρ***_***w***_], and ***M*** is the forward model component that produces the water reflectance. To project the retrieval error covariance matrix we therefore use

$${\hat{S}}_{b}^{-1}=J^{T}K^{T}S_{\epsilon }KJ+J^{T}S_{a}^{-1}J$$

Where ${\hat{S}}_{b}$ is the byproduct error covariance matrix. The indirect nature of this matrix may mean that it is has an implicit regularization. Comparison of ${\hat{S}}_{b}$ and the projection of the *a priori* error covariance matrix $J^{T}S_{a}^{-1}J$ can provide clues to the intermediate step constraints.

section Information Content Assessment contains an implementation of these techniques for several prototypical MAP designs for a variety of geophysical conditions. In practice, this means we construct Jacobian matrices to include all possible measurement spectral channels and geometries, and subset the Jacobian to represent the measurement system of interest. This is repeated for a variety of geophysical cases, and results presented in aggregate. In section Information Content Assessment we show the DFS as an overall metric of measurement capability, and ${\hat{S}}_{b}$ for water reflectance to show atmospheric correction capability.
