# Supplementary material for: Atmospheric Correction of Satellite Ocean-Color Imagery During the PACE Era
Source: Front Earth Sci (Lausanne). Author manuscript; Available in PMC 2020 May 21. (PMC7241613; doi:10.3389/feart.2019.00145)
Supplement: 1 [file NIHMS1540501-supplement-1.pdf]

frontiers in Earth Science

SUPPLEMENTARY MATERIAL
published: xx July 2019
doi: 10.3389/feart.2019.00145

OPEN ACCESS

Specialty section:
This article was submitted to Atmospheric Science, a section of the journal Frontiers in Earth Science

Received: *19 December 2018*
Accepted: *21 May 2019*
Published: *xx July 2019*

Citation:
Frouin RJ, Franz BA, Ibrahim A, Knobelspiesse K, Ahmad Z, Cairns B, Chowdhary J, Dierssen HM, Tan J, Dubovik O, Huang X, Davis AB, Kalashnikova O, Thompson DR, Remer LA, Boss E, Coddington O, Deschamps P-Y, Gao B-C, Gross L, Hasekamp O, Omar A, Pelletier B, Ramon D, Steinmetz F and Zhai P-W (2019) Atmospheric Correction of Satellite Ocean-Color Imagery During the PACE Era. Front. Earth Sci. 7:145. doi: 10.3389/feart.2019.00145

Atmospheric Correction of Satellite Ocean-Color Imagery During the PACE Era

Robert J. Frouin[1]*, Bryan A. Franz[2], Amir Ibrahim[2,3], Kirk Knobelspiesse[2], Ziauddin Ahmad[2,4], Brian Cairns[5], Jacek Chowdhary[5,6], Heidi M. Dierssen[7], Jing Tan[1], Oleg Dubovik[8], Xin Huang[8], Anthony B. Davis[9], Olga Kalashnikova[9], David R. Thompson[9], Lorraine A. Remer[10], Emmanuel Boss[11], Odele Coddington[12], Pierre-Yves Deschamps[8], Bo-Cai Gao[13], Lydwine Gross[14], Otto Hasekamp[15], Ali Omar[16], Bruno Pelletier[17], Didier Ramon[18], François Steinmetz[18] and Peng-Wang Zhai[19]

[1] Scripps Institution of Oceanography, University of California, San Diego, La Jolla, CA, United States, [2] Ocean Ecology Laboratory, NASA Goddard Space Flight Center, Greenbelt, MD, United States, [3] Science Systems and Applications Inc., Lanham, MD, United States, [4] Science Application International Corporation, McLean, VA, United States, [5] NASA Goddard Institute for Space Studies, New York, NY, United States, [6] Department of Applied Physics and Applied Mathematics, Columbia University, New York, NY, United States, [7] Department of Marine Science, University of Connecticut, Groton, CT, United States, [8] Laboratoire d'Optique Atmosphérique, Université de Lille, Villeneuve d'Ascq, France, [9] Jet Propulsion Laboratory, California Institute of Technology, Pasadena, CA, United States, [10] Joint Center for Earth System Technology, University of Maryland Baltimore County, Baltimore, MD, United States, [11] School of Marine Sciences, University of Maine, Orono, ME, United States, [12] Laboratory for Atmospheric and Space Physics, University of Colorado, Boulder, CO, United States, [13] Naval Research Laboratory, Washington, DC, United States, [14] Pixstart, Toulouse, France, [15] Earth Science Group, Netherlands Institute for Space Research, Utrecht, Netherlands, [16] Atmospheric Composition Branch, NASA Langley Research Center, Hampton, VA, United States, [17] Institut de Recherche Mathématique, Université de Rennes, Rennes, France, [18] HYGEOS, Euratechnologies, Lille, France, [19] Department of Physics, University of Maryland Baltimore County, Baltimore, MD, United States

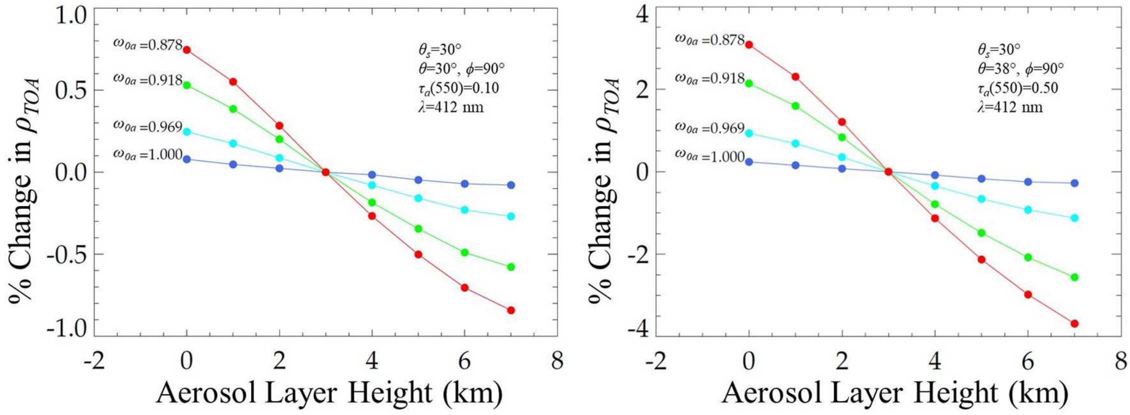

FIGURE 1 | Sensitivity of TOA reflectance to changes in aerosol layer height and aerosol single scattering albedo, ω_{0a}, at 412 nm for a specific solar and viewing geometry, i.e., $\theta_S = 30°$, $\theta = 38°$, and $\phi = 90°$. Chlorophyll-a concentration is 0.3 mgm^{-3}. The left-hand figure is for aerosol optical thickness, τ_a, of 0.1 and the right-hand side is for $\tau_a = 0.5$.

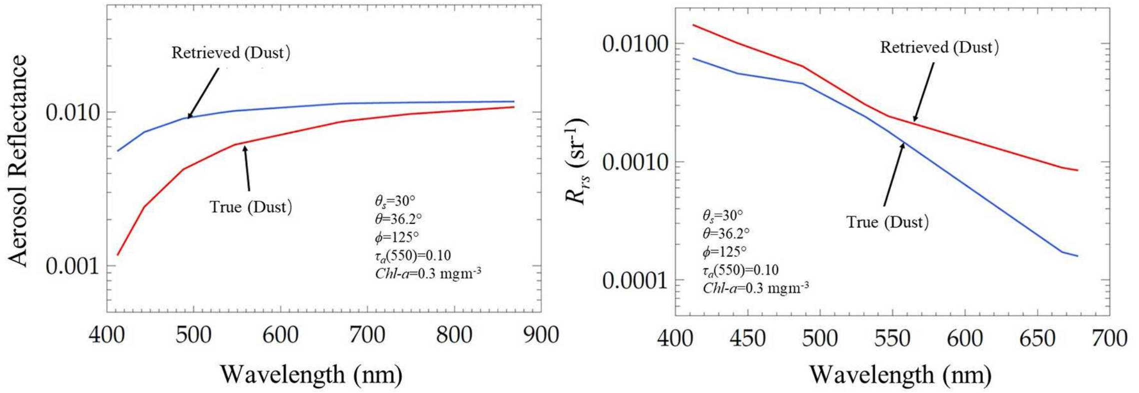

FIGURE 2 | The left-hand side figure is, for a specific solar and viewing geometry ($\theta_S = 30°$, $\theta = 36.2°$, and $\phi = 125°$), the aerosol reflectance at the TOA of the true (red) and retrieved (blue) dust model based on the heritage AC. The right-hand side figure shows the remote sensing reflectance after AC based on the true and retrieved aerosol model.

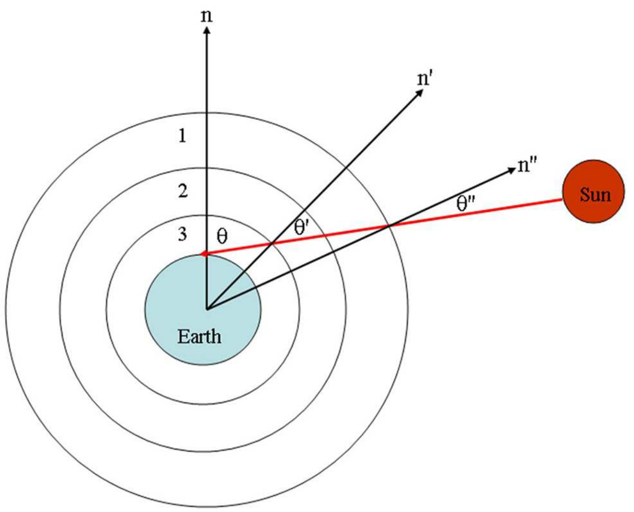

FIGURE 3 | Change in Sun zenith angle as the solar beam traverses through a spherical model atmosphere. θ, θ', and θ'' are the Sun zenith angles at the Earth's surface, the top of layer 3, and the top of the atmosphere, respectively. They should all be equal to θ.

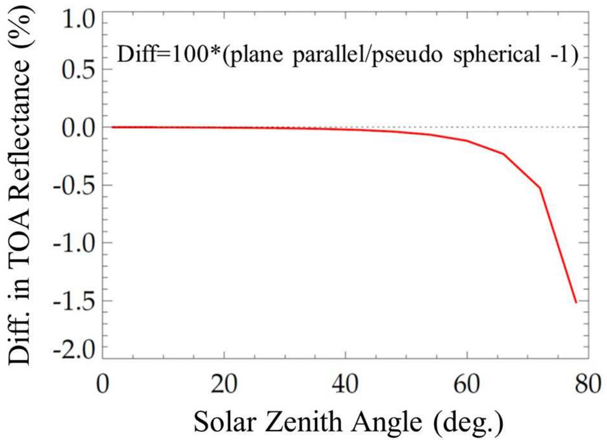

FIGURE 4 | The percent difference in TOA reflectance between plane-parallel and pseudo-spherical radiative transfer simulations for different Sun zenith angles. View zenith angle θ is 30° and relative azimuth angle ϕ is 144°.

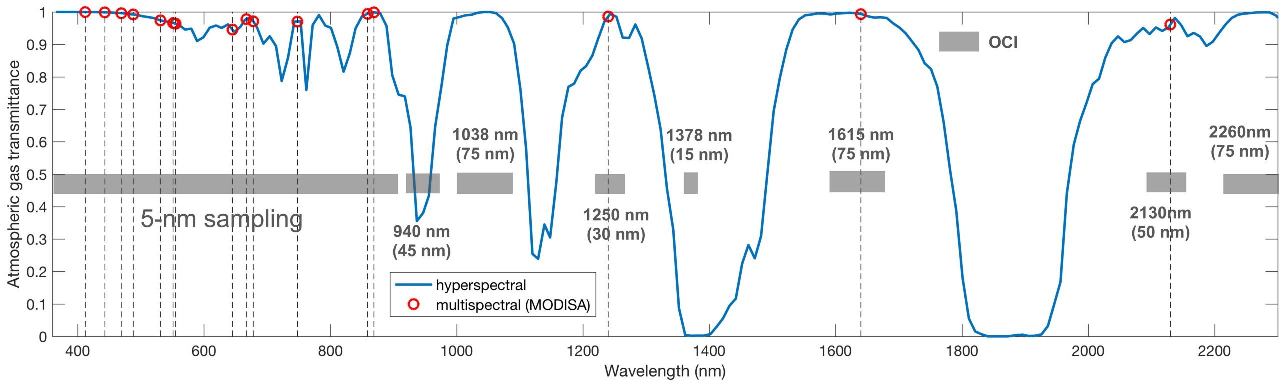

FIGURE 5 | The atmospheric gas transmittance including ozone, oxygen and water vapor (blue solid line). The red open circles show MODIS multispectral bands, while the gray shaded bar is the expected OCI bands from the UV to SWIR.

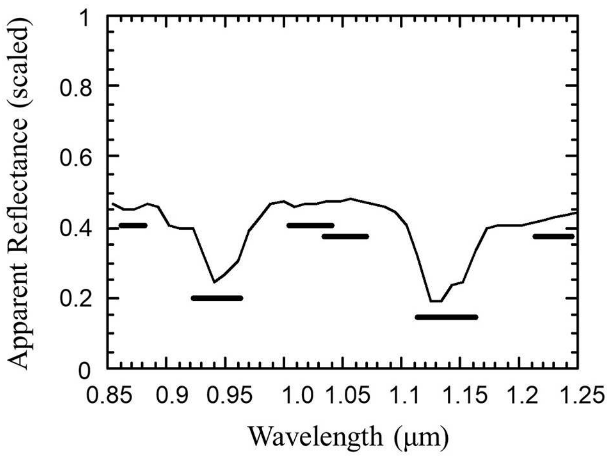

FIGURE 6 | TOA apparent reflectance spectrum with relevant positions and widths of spectral regions used in the three-channel ratio technique for 940 and 1,130 nm water vapor bands.

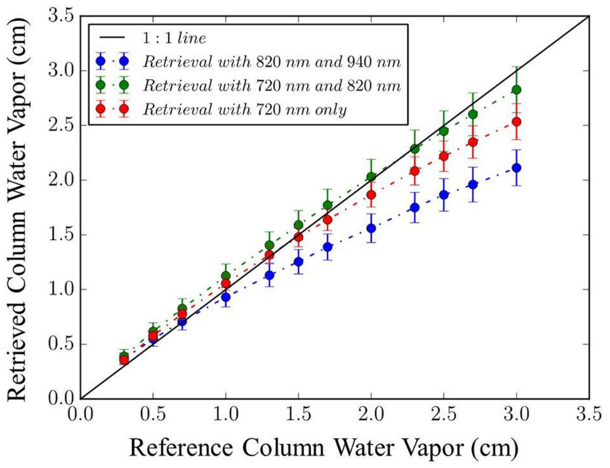

FIGURE 7 | Scatter plot between the assumed CWV in the VRT simulations and the retrieved CWV using a pair of water vapor channels at 820 and 940 nm (blue circles) and 720 and 820 nm (green circles), and 720 nm only (red circles). The error bar is the standard deviation due to changes in solar and viewing geometries (See text for simulation details). Reproduced with permission from Elsevier (After Ibrahim et al. 2018).

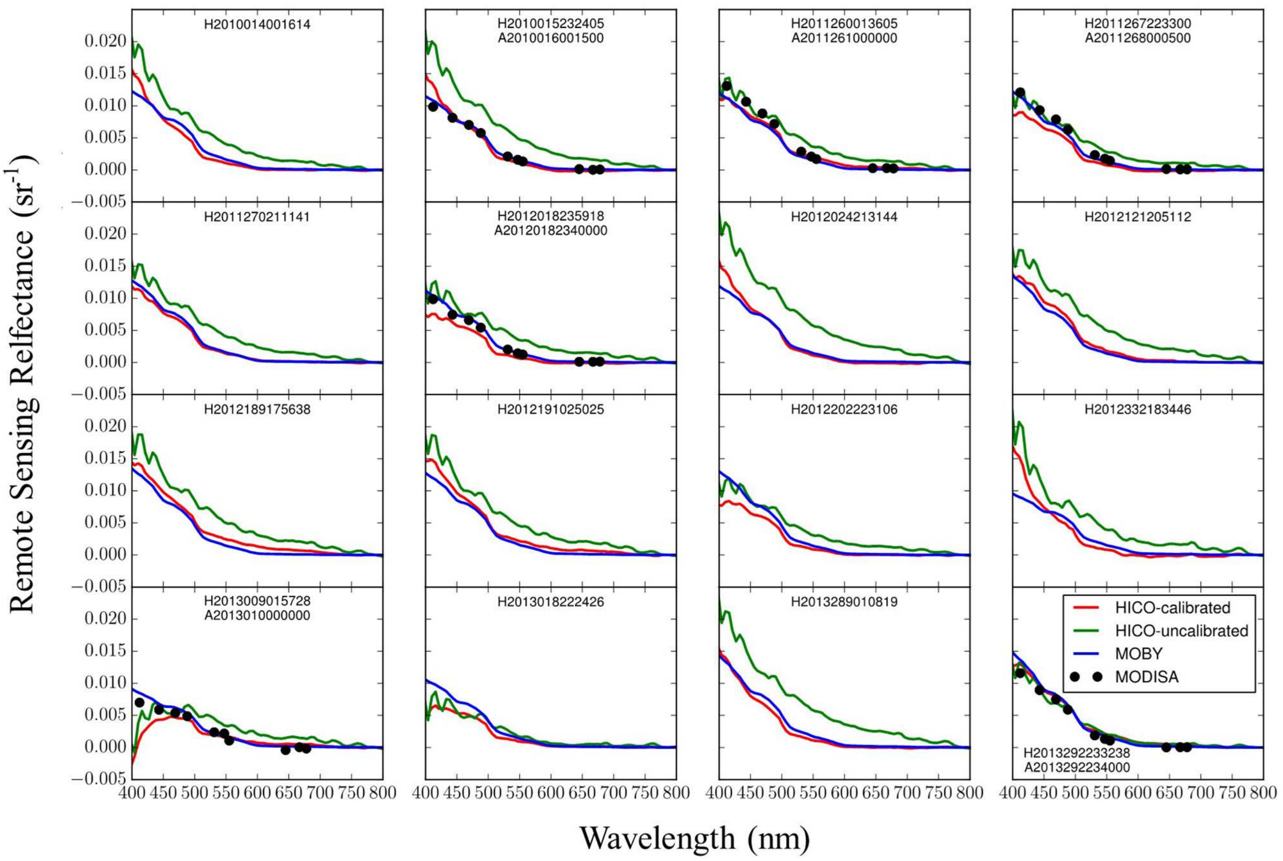

FIGURE 8 | The hyper-spectral R_{rs} matchups between *in-situ* MOBY data and HICO retrieval with and without applying the vicarious gain factors. The Figures title shows the scene ID from the NASA's ocean color webpage. Reproduced with permission from Elsevier (After Ibrahim et al. 2018).

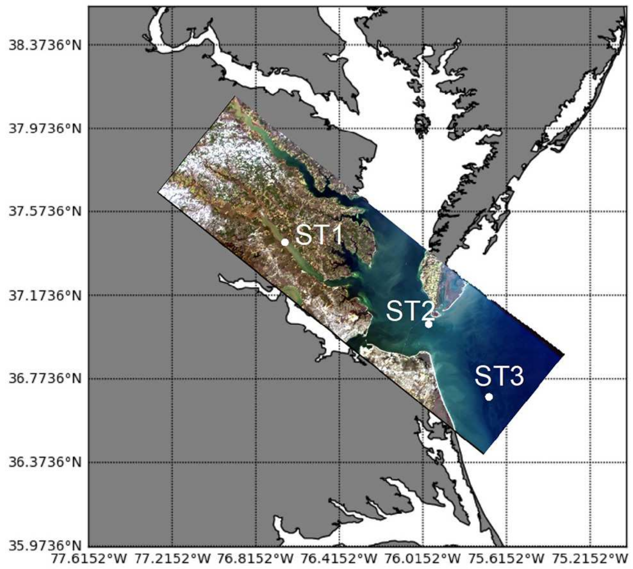

FIGURE 9 | True color image composite of HICO TOA radiance at 461, 553, and 639 nm of the Chesapeake Bay. Reproduced with permission from Elsevier (After Ibrahim et al. 2018).

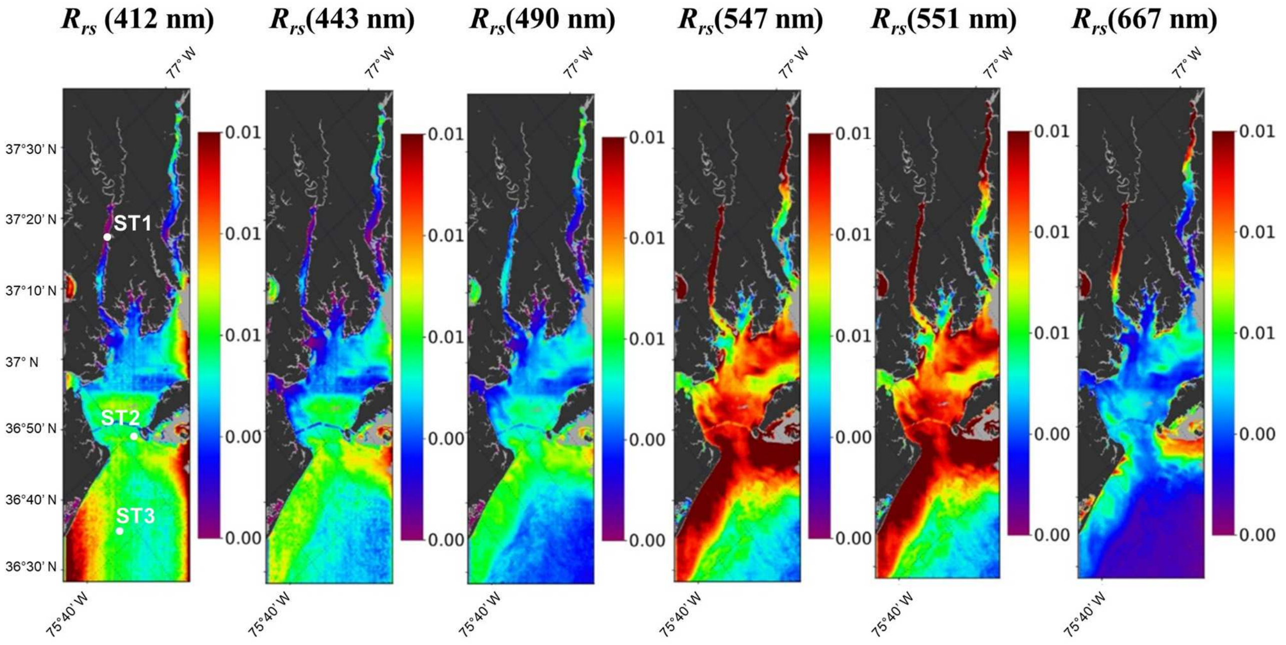

FIGURE 10 | The remote sensing reflectance (R_{rs}) from HICO imagery at the Chesapeake Bay on the east coast of the US at selected wavelengths within the visible spectrum. The R_{rs} retrieval is based on the heritage AC algorithm.

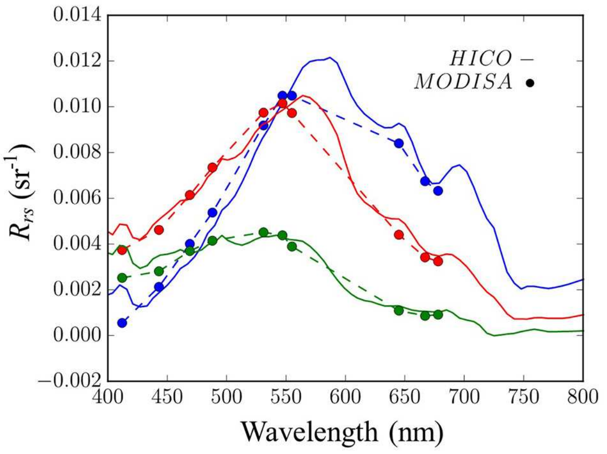

FIGURE 11 | A comparison of the spectral R_{rs} between MODIS-A (dashed lines) and HICO (solid lines) retrievals at three locations in the Chesapeake Bay. Blue, red, and green curves denote Stations 1, 2, and 3, respectively. Reproduced with permission from Elsevier (After Ibrahim et al. 2018).

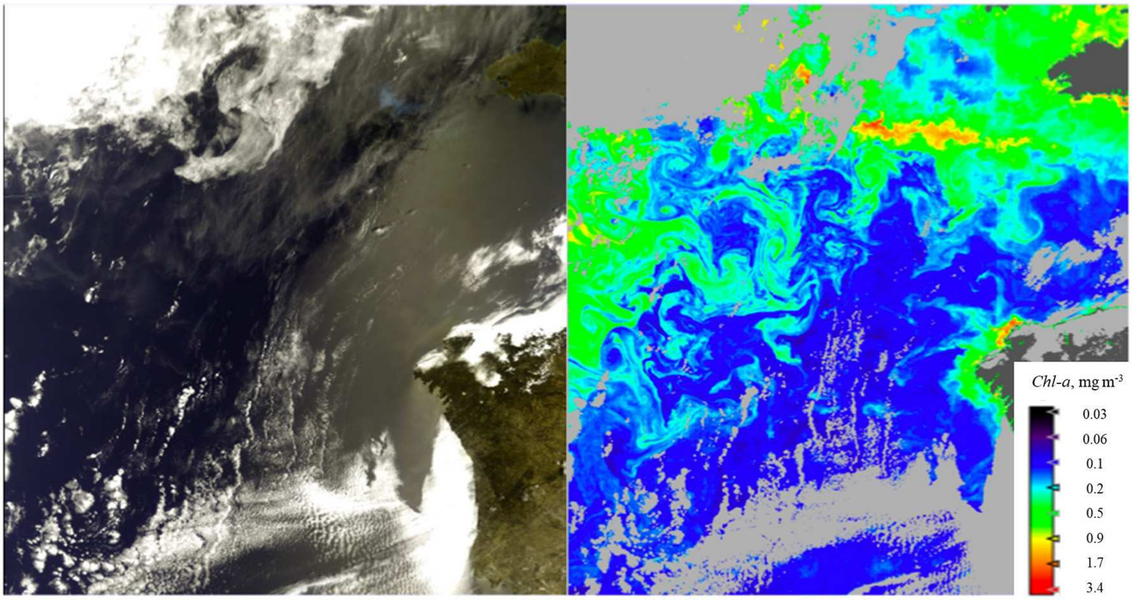

FIGURE 12 | RGB composite of a MERIS scene off Portugal, 21 June 2005 **(left)** and chlorophyll-a concentration derived by the POLYMER algorithm **(right)**. Chlorophyll-a concentration is retrieved in the presence of thin clouds and Sun glint, and the chlorophyll-a patterns exhibit spatial continuity from cloud- and glint-free areas to adjacent cloud- and/or glint-contaminated areas.

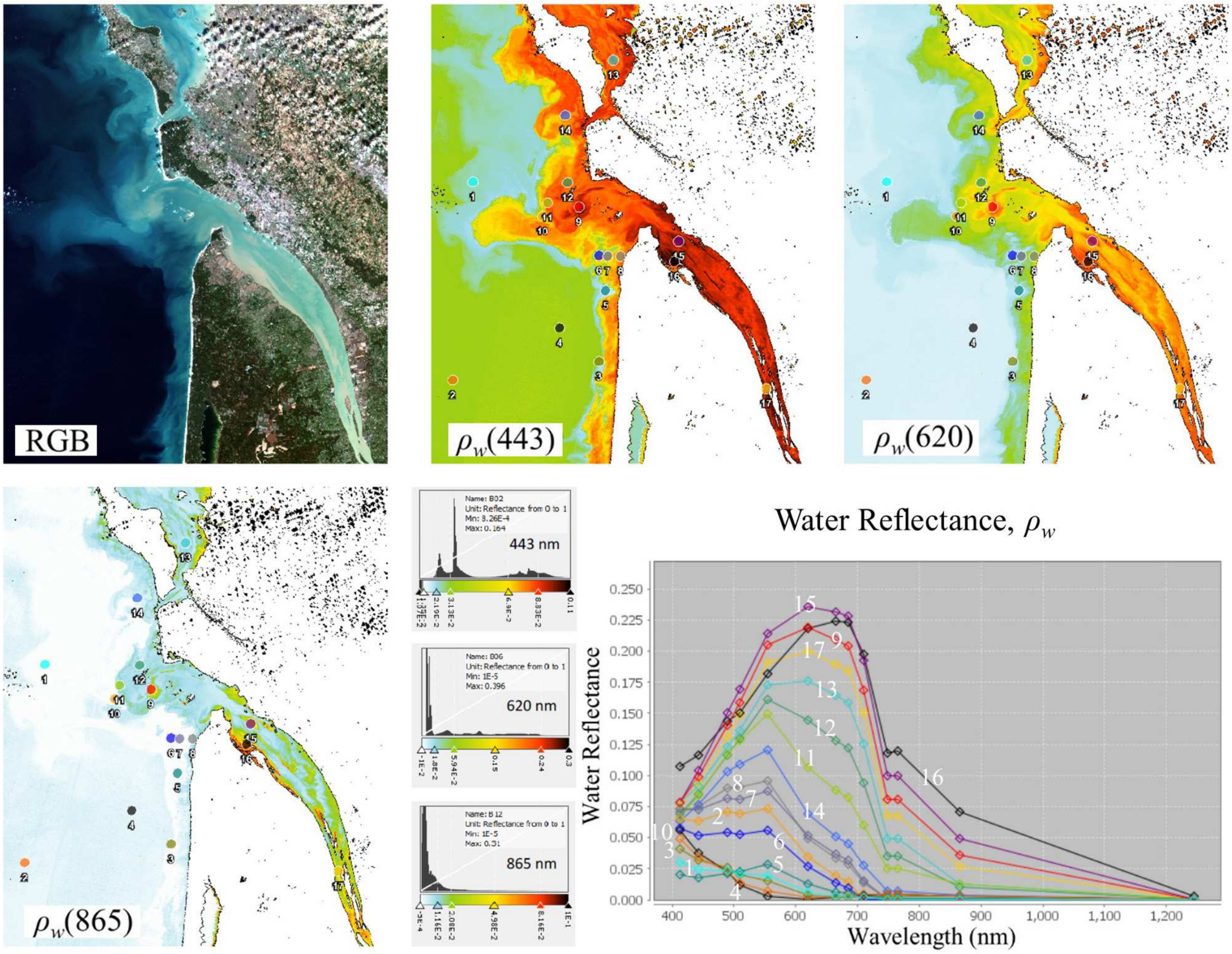

FIGURE 13 | Water reflectance estimated from Sentinel-2 MSI data at 30 m resolution over the Gironde river estuary, France, on 21 October 2016. **Top left**: RGB composite; **Top center**: water reflectance at 443; **Top right**, water reflectance at 620 nm; **Bottom left**: water reflectance at 865 nm; **Bottom center**: Histogram of retrieved water reflectance values and color scale for the three images; and **Bottom right**: selected water reflectance spectra in various parts of the images (depicted by colored circles in the images).

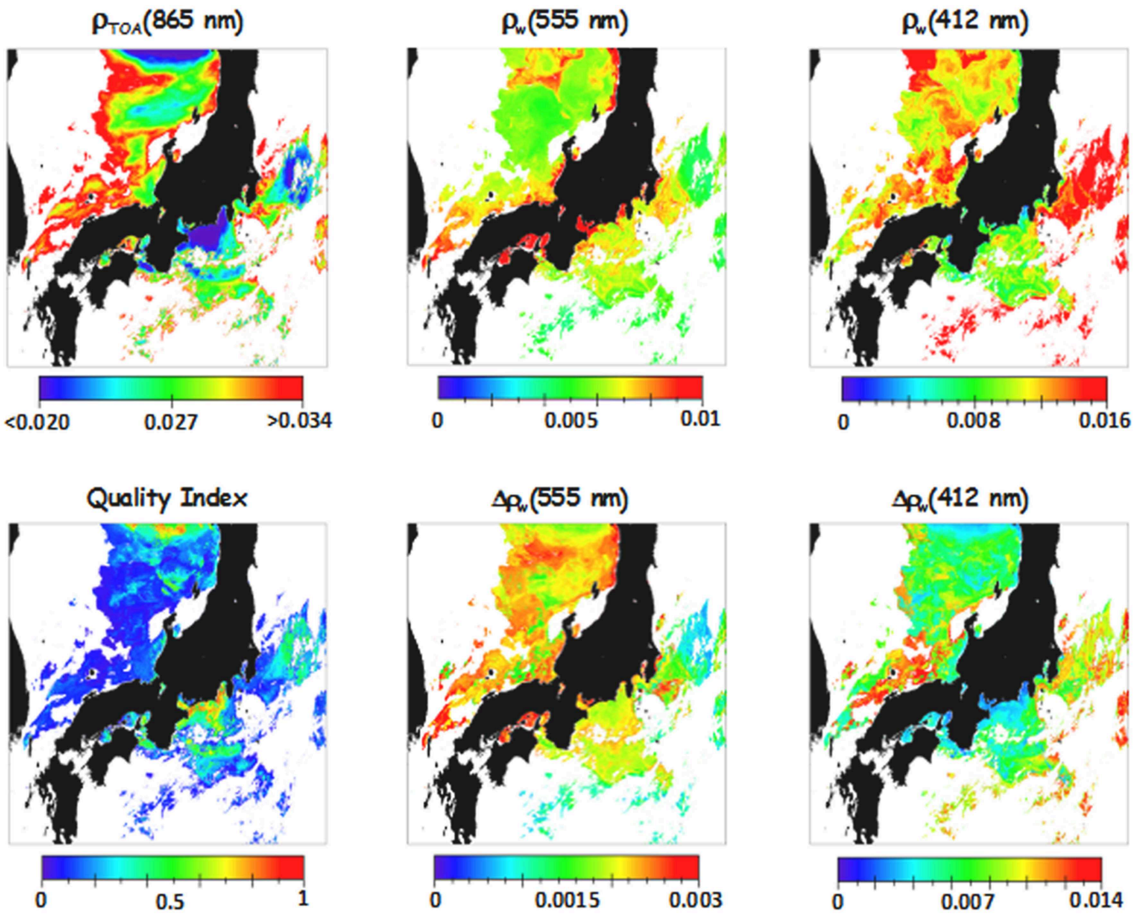

FIGURE 14 | Application of Bayesian inverse methodology (Frouin and Pelletier, 2015) to SeaWiFS imagery of the Sea of Japan and northwest Pacific on April 7, 2001 during the ACE-Asia experiment. Clockwise: TOA reflectance at 685 nm, retrieved water reflectance at 555 and 412 nm, uncertainties in estimates at 555 and 412 nm, and quality index (p-value).

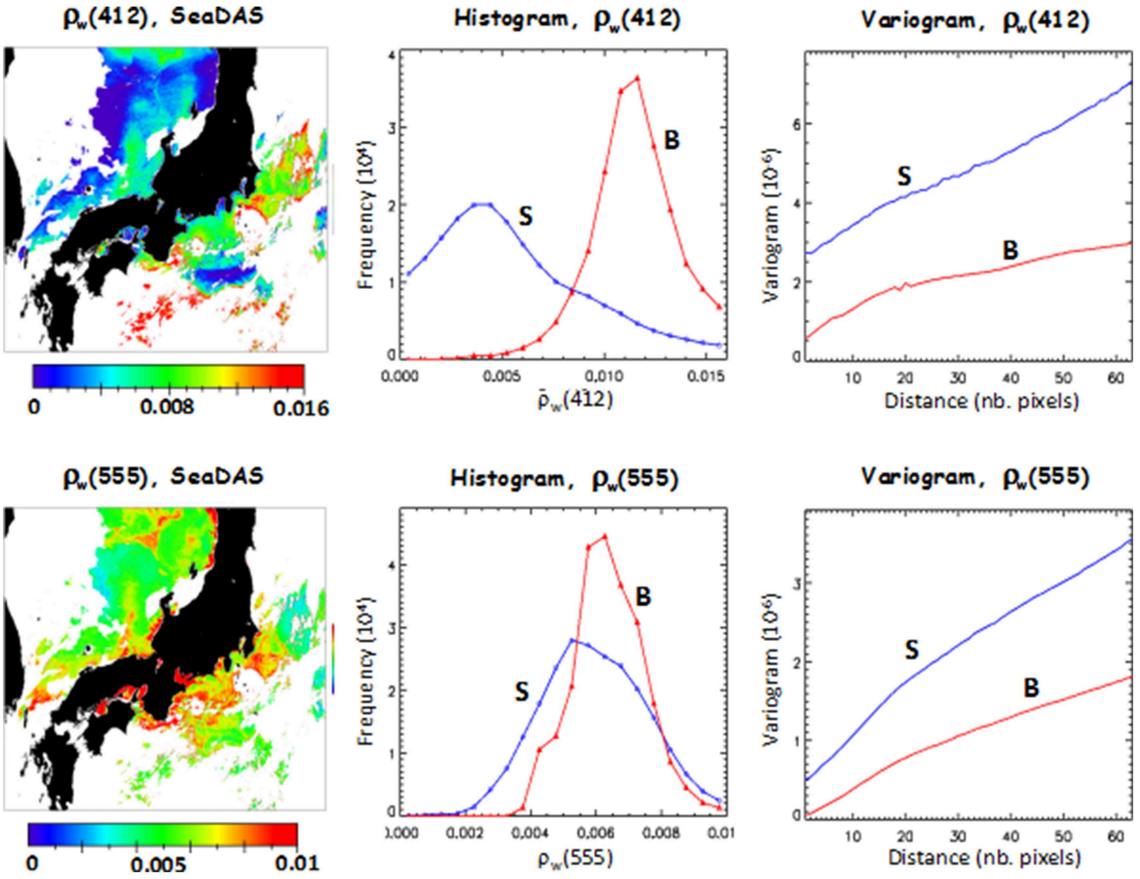

FIGURE 15 | Left: Marine reflectance at 412 and 555 nm retrieved by the standard SeaDAS algorithm in the Sea of Japan and northwest Pacific (SeaWIFS imagery of April 7, 2001); Center: Histograms of water reflectance at 412 and 555 nm obtained from valid data, SeaDAS (S) and Bayesian (B) algorithms; Right: Variograms of water reflectance at 412 and 555 nm obtained for a 150×150 pixel area in the Sea of Japan, SeaDAS (S) and Bayesian (B) algorithms.

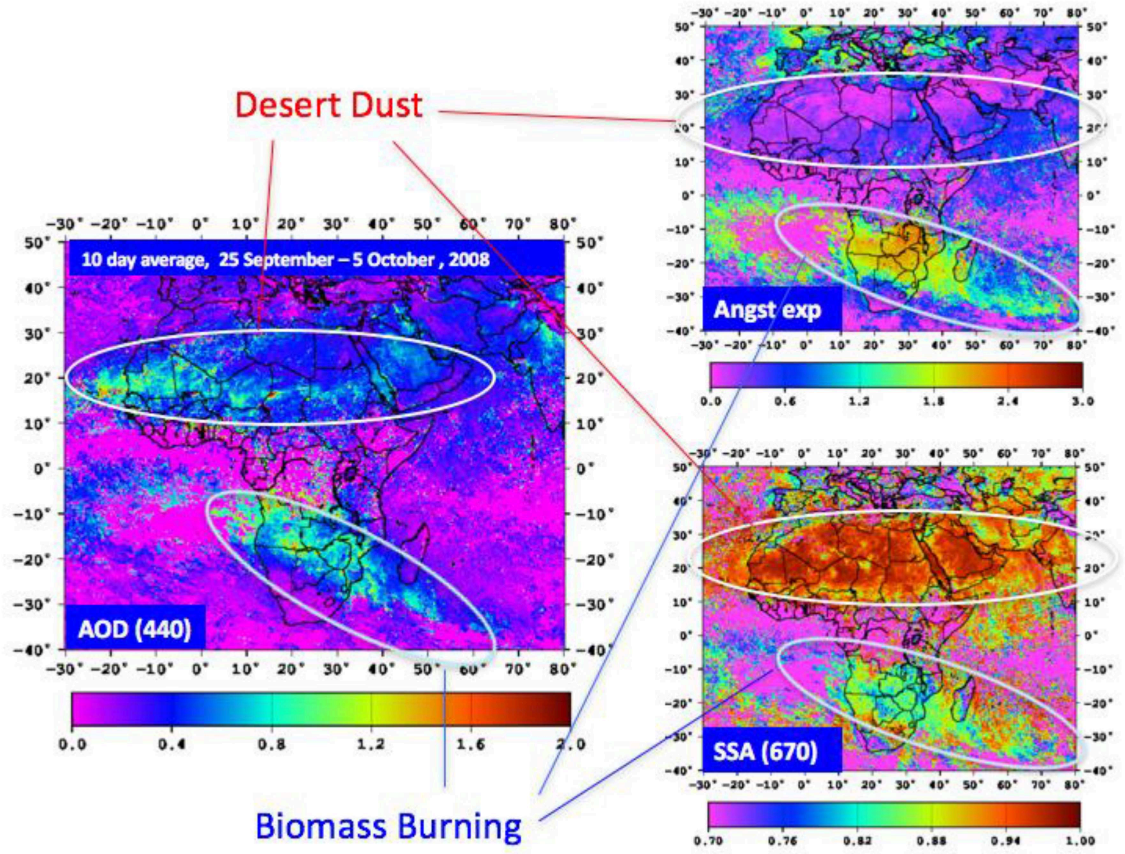

FIGURE 16 | Illustration of PARASOL/GRASP retrieval, 10-day average for the period from 25 September to 5 October 2008. Left panel shows aerosol optical thickness at 440 nm, right panel shows Angstrom exponent (upper part) and aerosol single scattering albedo at 670 nm (lower part).

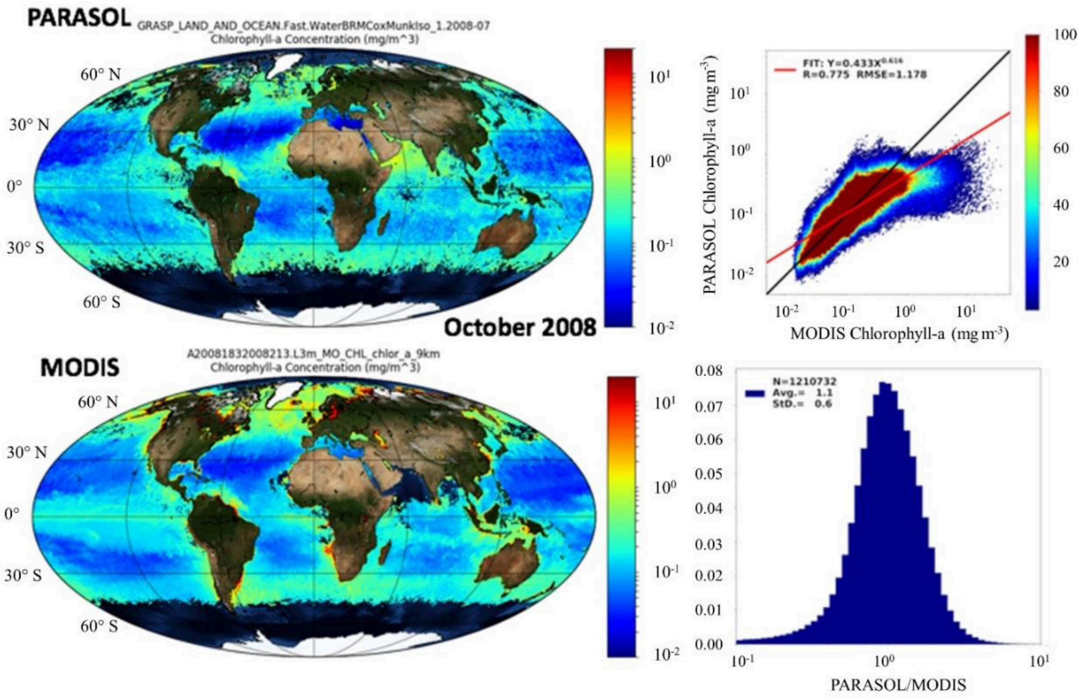

FIGURE 17 | Left: PARASOL/GRASP retrieval of chlorophyll-a concentration, monthly average for October 2008, 0.1° resolution (upper part), MODIS retrieval (NASA standard algorithm) of chlorophyll-a concentration, monthly average for October 2008 9 km resolution (lower part). Right: Correlation of PARASOL/GRASP retrieved chlorophyll-a concentration with MODIS values for October 2008.

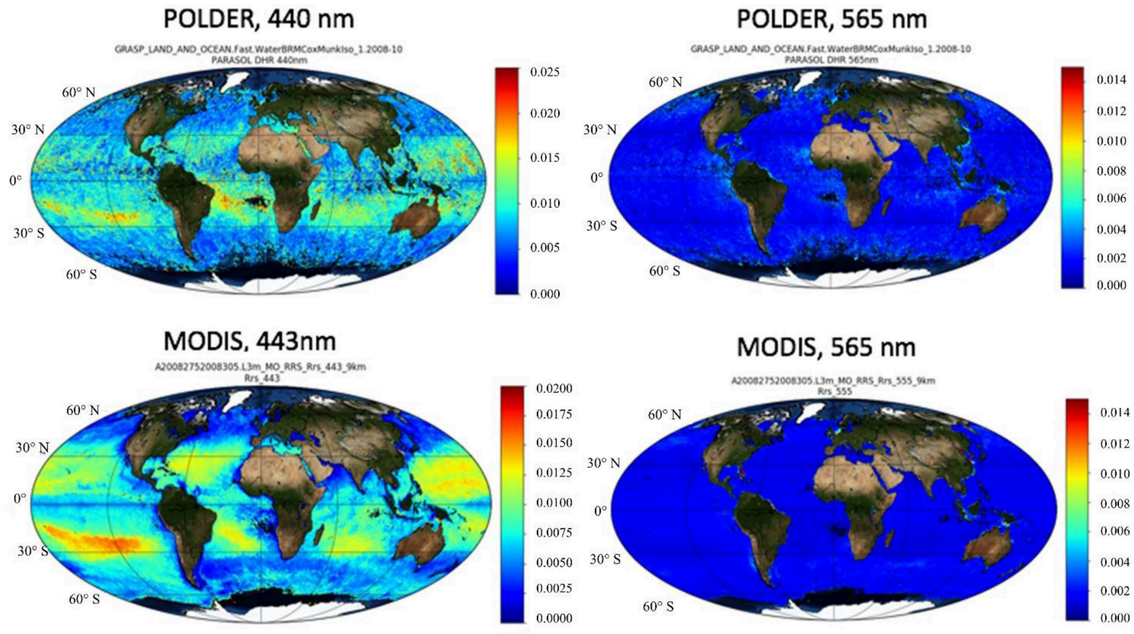

FIGURE 18 | Comparison between POLDER/PARASOL and MODIS-A imagery of remote sensing water reflectance (October 2008) obtained from NASA standard algorithm (MODIS) and GRASP algorithm (POLDER).

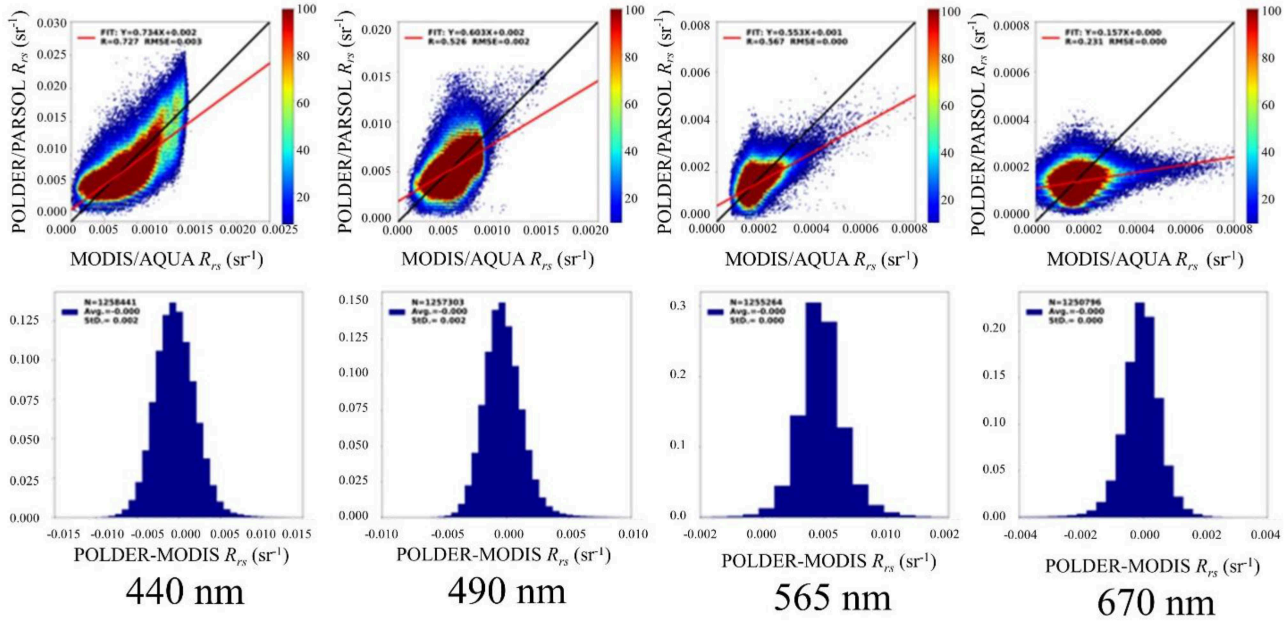

FIGURE 19 | Comparison between POLDER/PARASOL and MODIS-A retrievals of remote sensing reflectance (entire 2008 year) obtained from GRASP algorithm (POLDER) and NASA standard algorithm (MODIS).

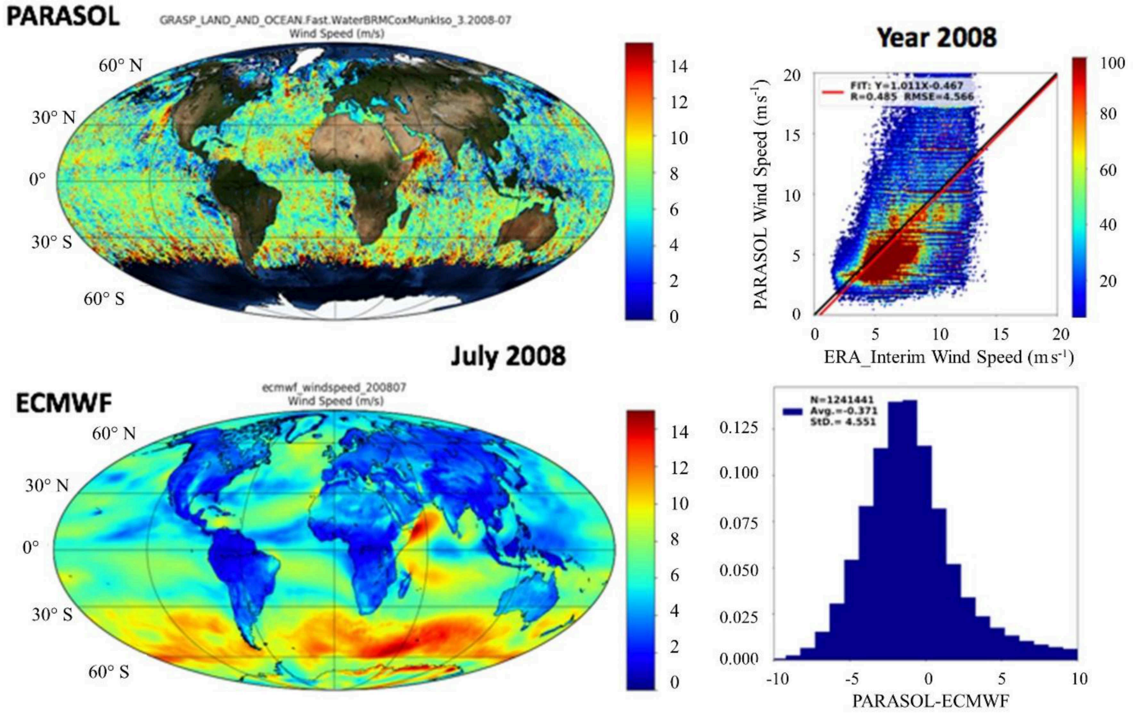

FIGURE 20 | Left: PARASOL/GRASP retrieval of wind speed, monthly average for July 2008, 10 km resolution (upper part), ECMWF wind speed, monthly average for July 2008, 10 km resolution (lower part). Right: Correlation of PARASOL/GRASP retrieved wind speed with ECMWF values for entire 2008.

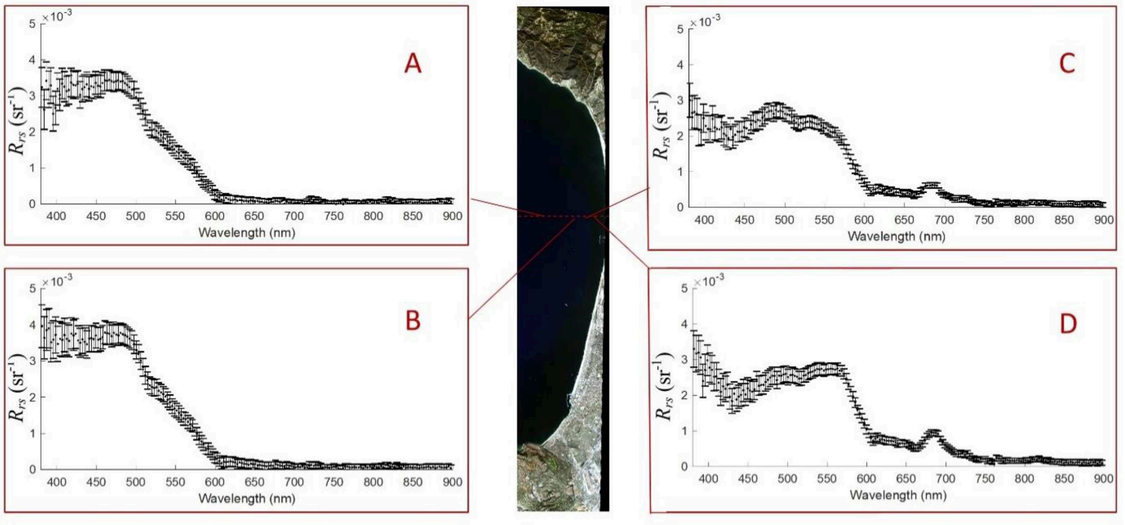

FIGURE 21 | PRISM transect of Santa Monica Bay acquired on 26 Oct. 2015, with panels showing representative Case 1 and Case 2 R_{rs} spectra. Panels A and B show two examples of dark water spectra, with error bars indicating 95% posterior predictive uncertainties. Panels C and D show two examples of more productive and turbid water. R_{rs} was retrieved using the optimal estimation approach of Thompson et al. (2018). The spectra show phytoplankton fluorescence at 685 nm.

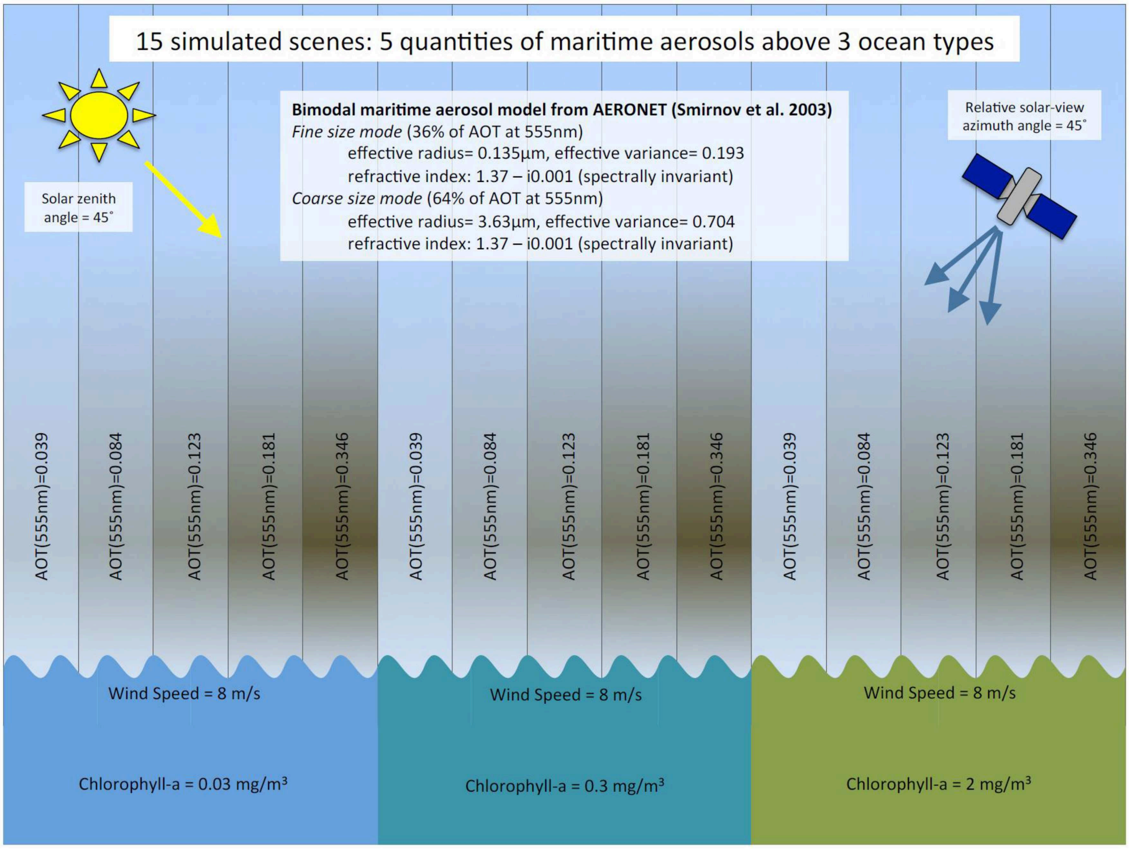

FIGURE 22 | Simulation characteristics for information content study.

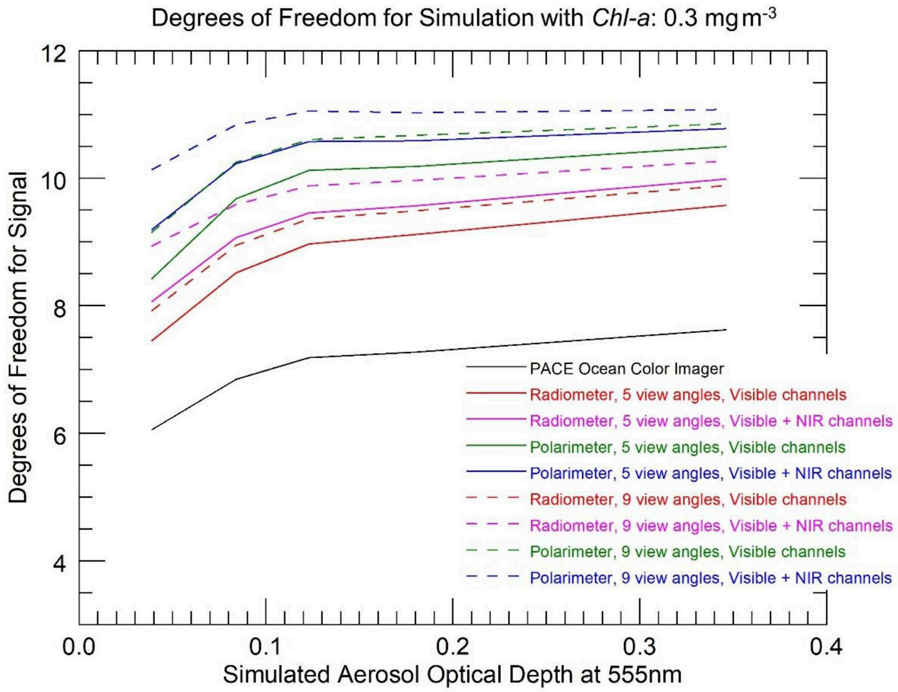

FIGURE 23 | Degrees of Freedom for Signal (DFS) for various MAP designs. Details about the various cases are provided in Table 2, corresponding colors.

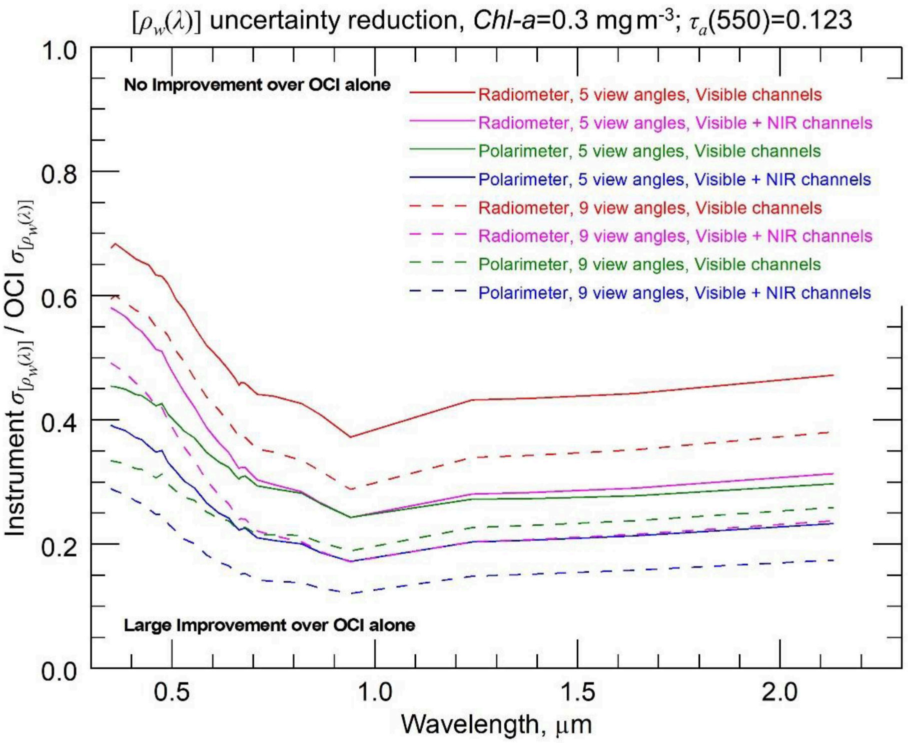

FIGURE 24 | Ratio of MAP water reflectance to that of OCI. Cases are the same as those of **Figure 23** (see Table 2 for details).

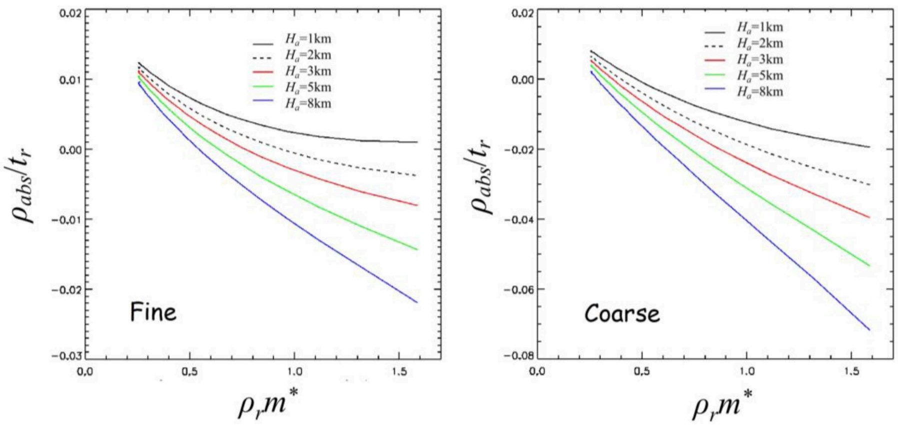

FIGURE 25 | Simulated ρ_{abs}/t_r, versus $\rho_r m^*$ for fine aerosols and coarse aerosols, left and right, respectively. Wavelength is 412 nm and aerosol optical thickness is 0.3. Wind speed is 5 m s^{-1} and marine reflectance is 0.02. Solar zenith angle is 30°, viewing zenith angle varies between 0° and 80°, and relative azimuth angle is 90°. Aerosol scale height, H_a, varies from 1 to 8 km (8 km correspond to mixed aerosols and molecules). The fine aerosols are defined by radius $r_f = 0.1$ µm, dispersion $\sigma_f = 0.20$, and index of refraction $m_f = 1.40 – 0.010i$ (ω_{0a} of 0.94), and the coarse aerosols by $r_c = 2.0$ µm, $\sigma_c = 0.30$, m_c and $= 1.55 – 0.002i$ (ω_{0a} of 0.88).

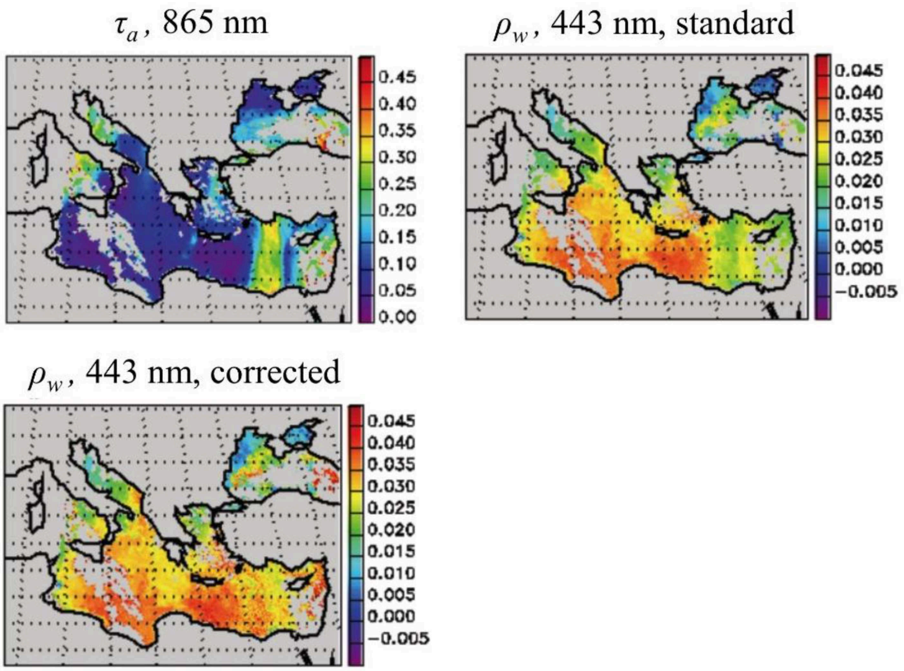

FIGURE 26 | Application of the multi-angle algorithm to POLDER imagery over the Mediterranean Sea during a dust outbreak from Africa. In the Eastern part of the basin, contaminated by dust, the multi-angle algorithm (lower left) gives higher values than the standard algorithm, consistent with values in adjacent regions not affected by dust.

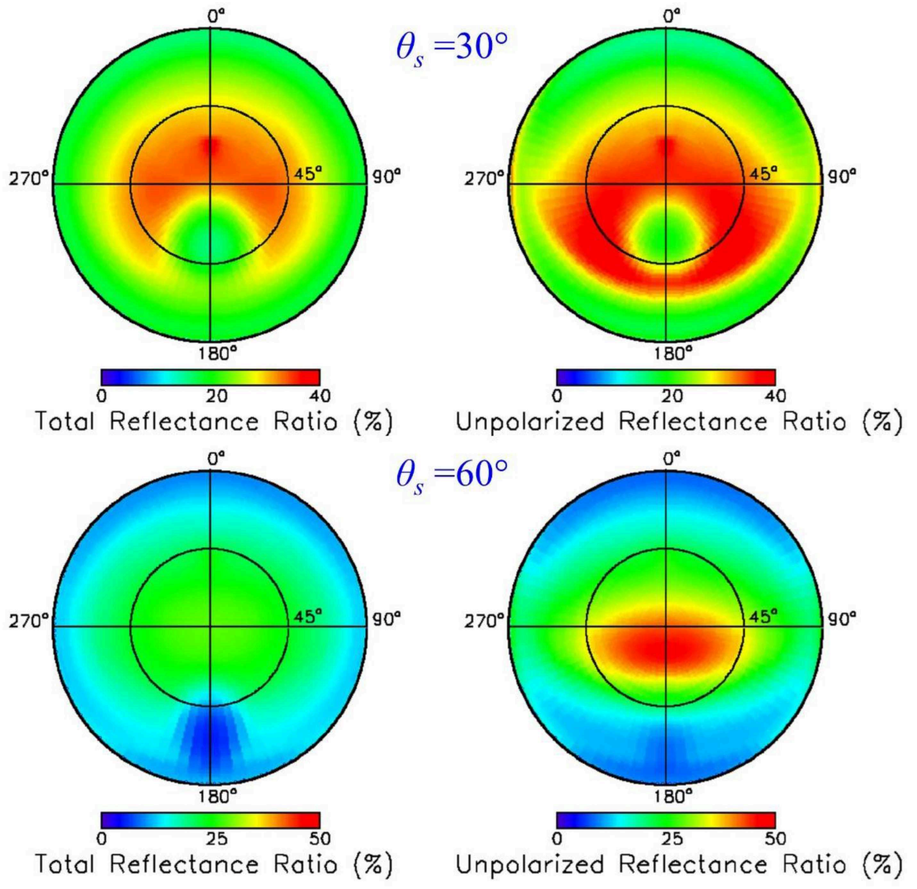

FIGURE 27 | Ratios of below-surface (0-) water reflectance to TOA reflectance, total (left) and unpolarized (right) for ocean-atmosphere system with WMO maritime aerosols, τ_a of 0.1 at 550 nm, *Chl-a* of 0.1 mgm^{-3}, and wind speed of 5 m s^{-1}. Wavelength is 443 nm, solar zenith angle θ_S is 30° (top) and 60° (bottom). Ratio is generally higher for unpolarized reflectance, especially when θ_S = 60°.

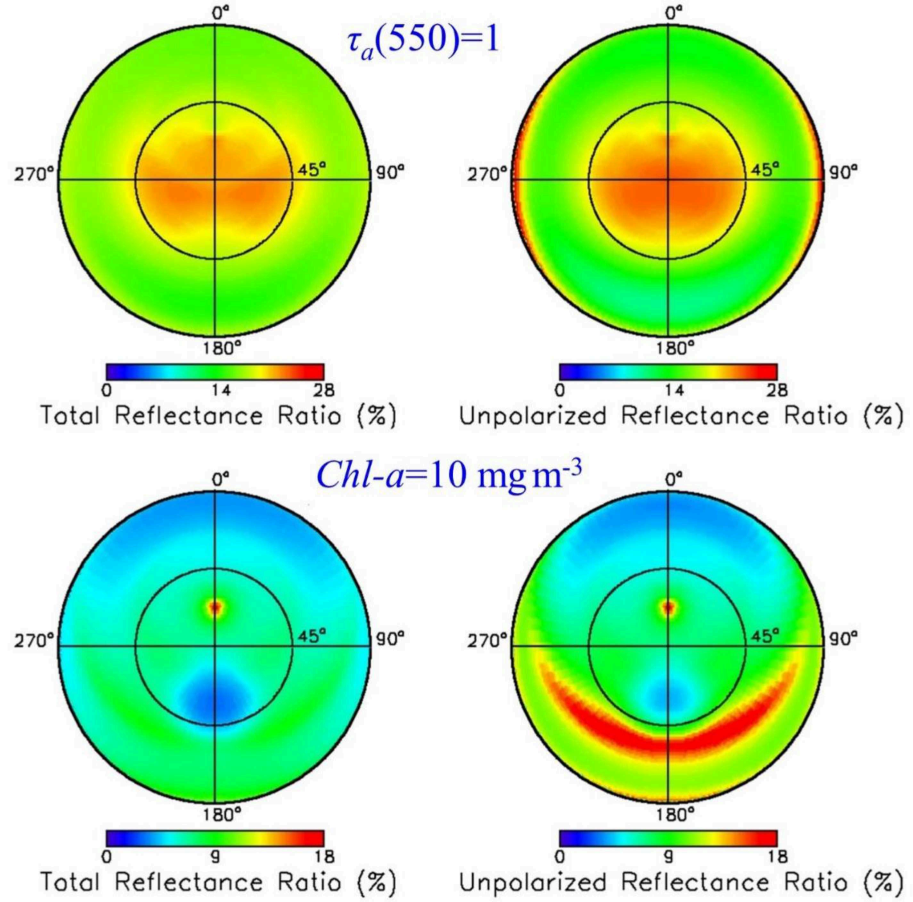

FIGURE 28 | Same as **Figure 27**, but τ_a = 1 at 550 nm (top) and *Chl-a* = 10 mgm^{-3} (bottom). No or small enhancement of the surface signal when τ_a is increased to 1, due to multiple scattering, but large enhancement when *Chl-a* is 10 mgm^{-3} at scattering angles of about 90° in the forward direction.

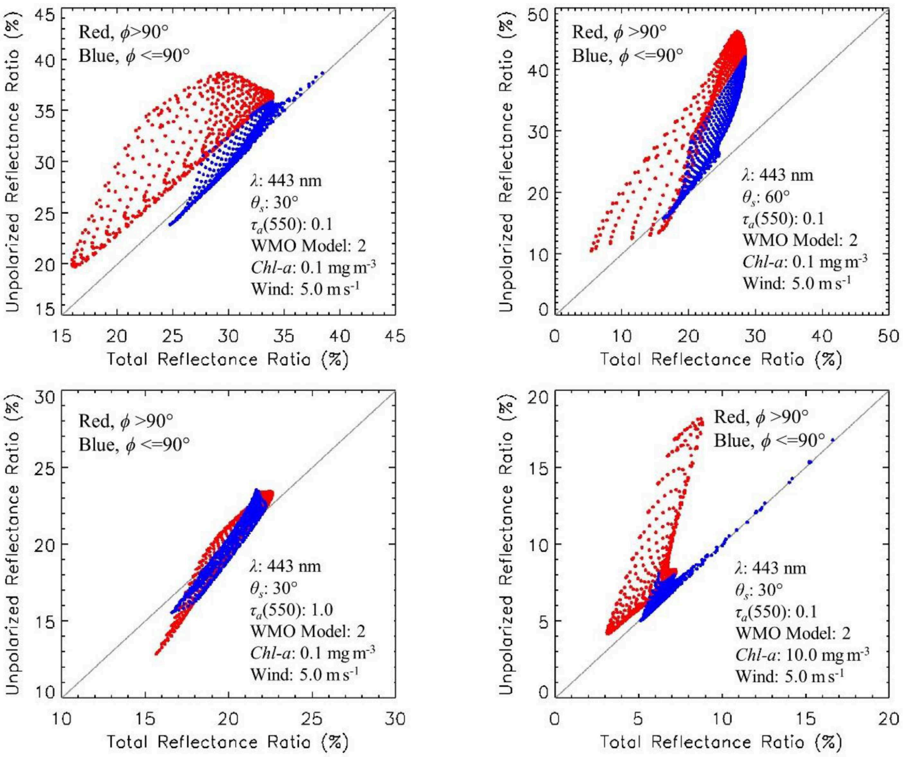

FIGURE 29 | Unpolarized versus total reflectance ratio (0-/TOA) at 443 nm for the situations of **Figures S27**, **S28** and view zenith angles less than 60°. Top left: θ_S = 30°, τ_a = 0.1 at 550 nm, *Chl-a* = 0.1 mgm^{-3}. Top right: θ_S = 60°, τ_a = 0.1 at 550 nm, *Chl-a* = 0.1 mgm^{-3}. Bottom left: θ_S = 30°, τ_a = 1 at 550 nm, *Chl-a* = 0.1 mgm^{-3}. Bottom right: θ_S = 30°, τ_a = 0.1 at 550 nm, *Chl-a* = 10 mgm^{-3}. Red points: ϕ >90° (forward scattering); Blue points: ϕ <90° (backward scattering). Unpolarized ratio is generally higher than total ratio, except when τ_a is large.

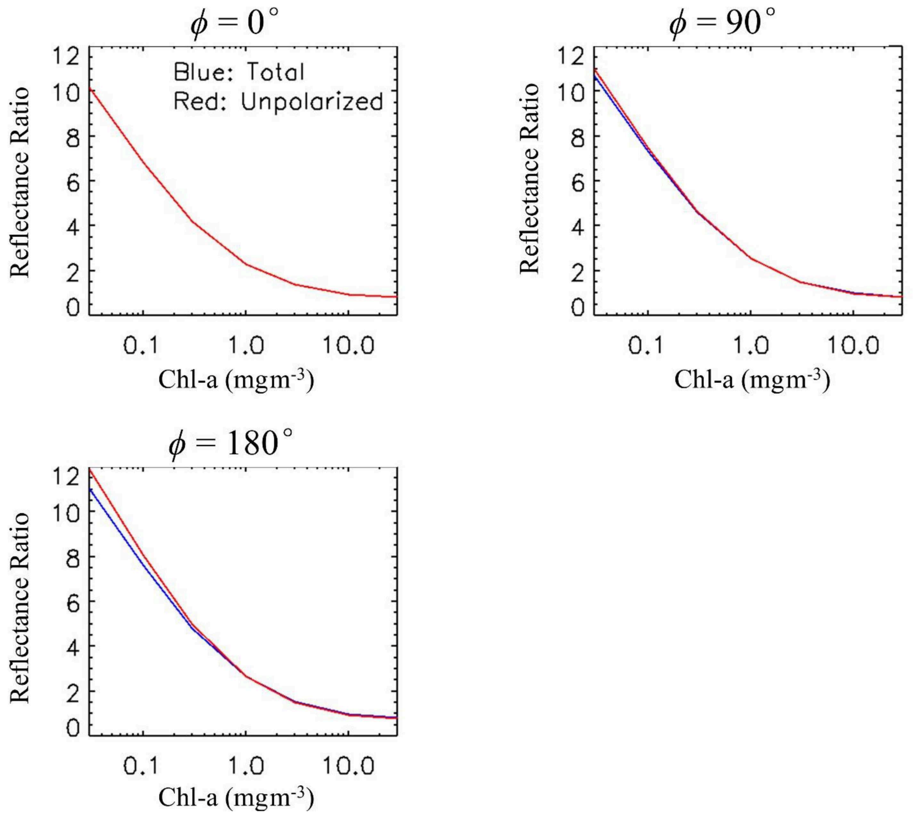

FIGURE 30 | Sensitivity of ratio of water reflectance just below the surface at 443 nm and 550 nm to chlorophyll-a concentration *Chl-a* (mgm^{-3}). Total and unpolarized signals are used (blue and red curves). Atmospheric and surface conditions are those of **Figure 27**. Solar and viewing zenith angles are 30°. Variation with *Chl-a* is similar using total or unpolarized reflectance.

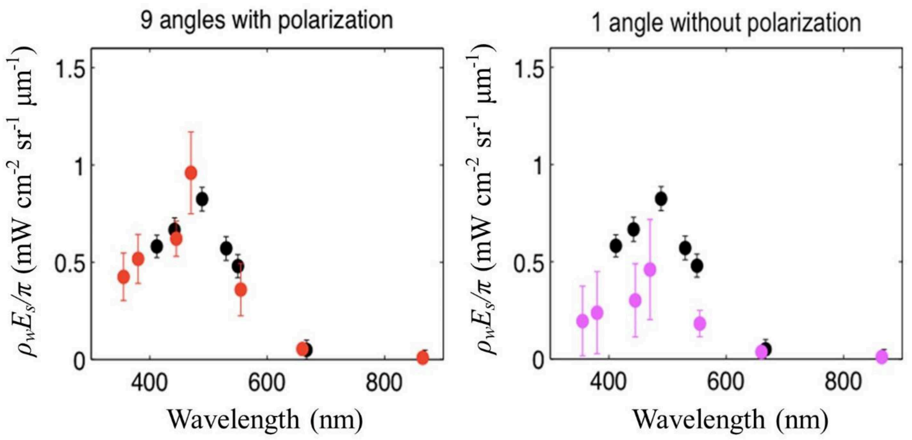

FIGURE 31 | Mean and spread of AirMSPI $\rho_S E_S/\pi$ (also known as normalized water-leaving radiance, L_{wn}) retrieval results based on 8 initial guesses. Black symbols: SeaPRISM observations with error bars denoting the PACE SDT uncertainty target. The left-hand panel contains results derived from observations at 9 angles; radiances at 355, 385, 445, 470, 555, 660, and 865 nm; and polarization in the 470, 660, and 865 nm bands. Multi-angle radiometry and polarimetry appear capable of retrieving accurate L_{wn} without the need for prescribed aerosol or surface reflectance constraints, even at a mid-visible aerosol optical thickness of 0.25. The right-hand panel contains results derived from multispectral observations at a single angle without polarization, and shows that without additional information there is an increased bias and modeling uncertainty in the retrieved L_{wn}.

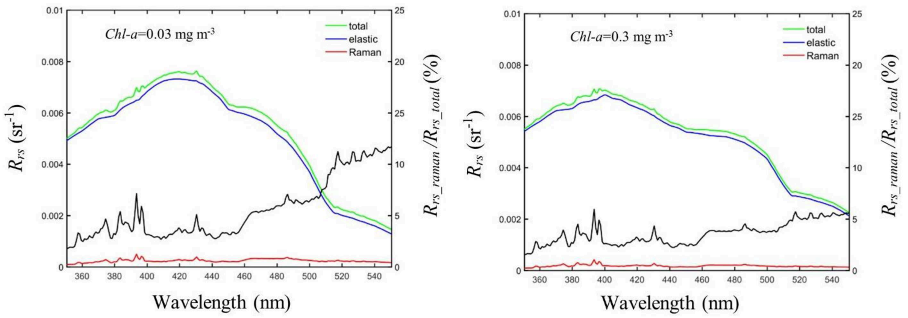

FIGURE 32 | Results of numerical simulations performed with HYDROLIGHT for chlorophyll-a concentrations of 0.03 and 0.3 mgm^{-3} (left and right, respectively). CDOM absorption coefficient is 0.01 m^{-1} at 443 nm The total, Raman, and elastic above surface "remote sensing" reflectance, as well as the proportion (in %) of Raman scattered light in the total "remote sensing" reflectance are given, showing the importance of Raman scattering in clear waters.

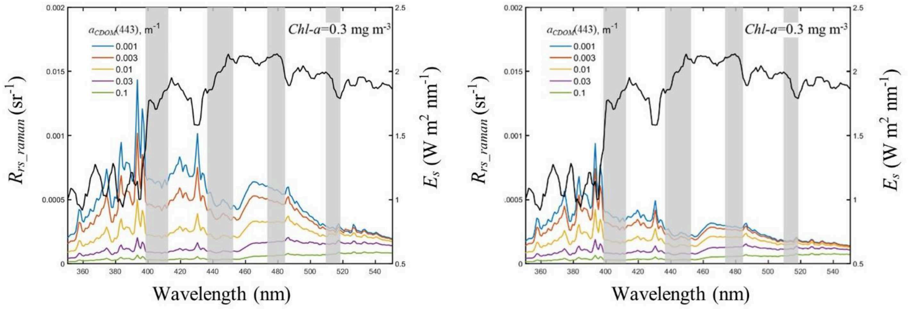

FIGURE 33 | Solar irradiance, E_S, and Raman "remote sensing" reflectance, R_{rs_raman}, in the range 350–550 nm at a 5 nm resolution every 1.5 nm, black and colored curves, respectively. Several chlorophyll-a concentrations, namely 0.03 and 0.3 mgm^{-3} (left and right, respectively), and CDOM absorption coefficients are used. Some intervals, namely 398.5–412.5 nm, 436.5–452.5, 473.5–484.5 nm, and 509.5–519.5 nm (depicted in grey), in which the Raman signal is fairly constant and E_S sufficiently variable, may be suitable to separate the Raman and elastic contributions to the TOA signal (see text for details).

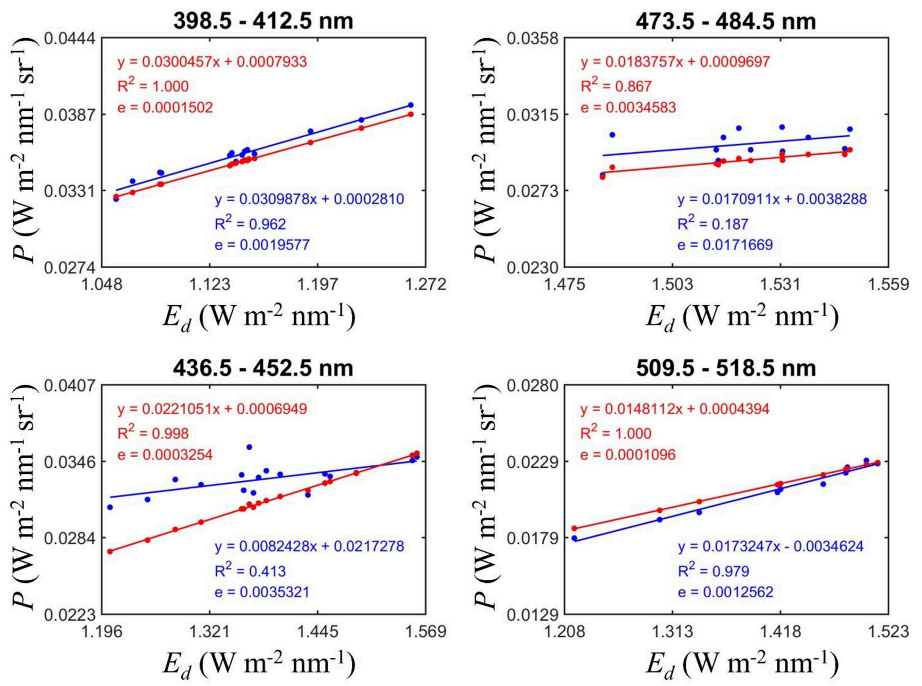

FIGURE 34 | Parameter P versus E_d for various spectral intervals. The ordinate of the best linear fit at the origin gives the average Raman water-leaving radiance $< L_{w\_raman} >$ in the spectral interval considered. Chlorophyll-a concentration is 0.03 mgm^{-3}. The blue curves correspond to $F = 0$ and the red curves includes the spectral correction F for the prescribed Chlorophyll-a concentration.

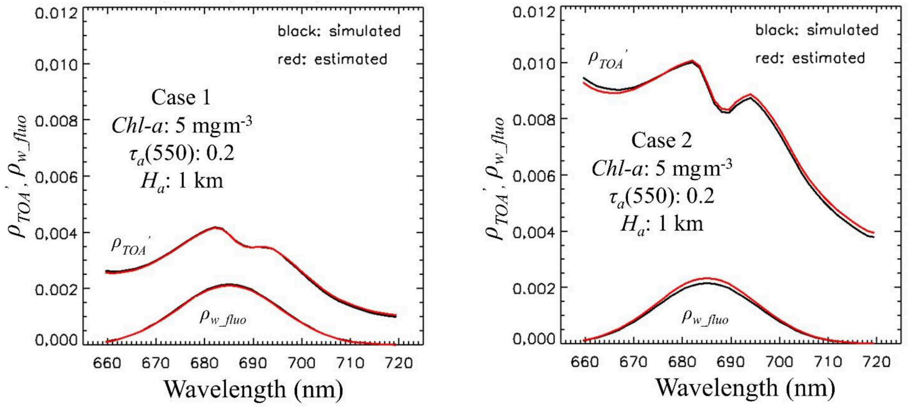

FIGURE 35 | Estimation of fluorescence signal (ρ_{w_fluo}) by spectral optimization using hyper-spectral measurements in the O2 B-band for typical Case 1 and Case 2 waters with chlorophyll-a concentration of 5 mgm^{-3} (left and right, respectively). Black and red curves correspond to actual and estimated values. Aerosol optical thickness is 0.2 and aerosol scale height is 1 km (unknown, fixed at 0.5 km in the optimization scheme). Retrieval accuracy is <5% for both water types.

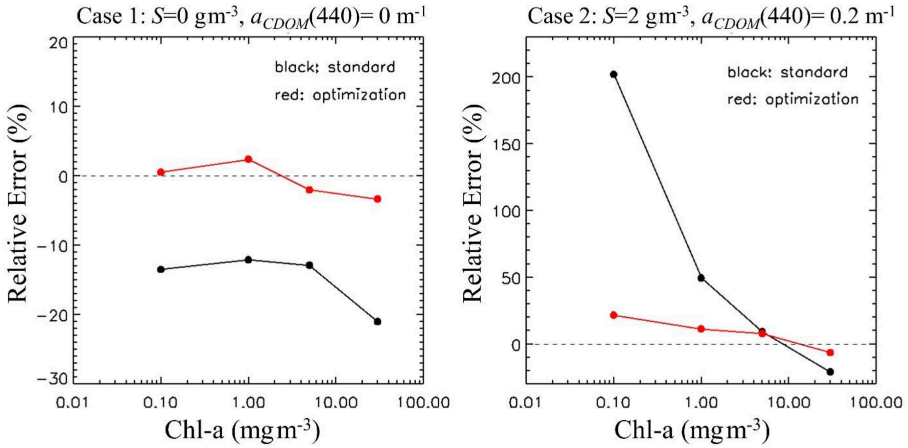

FIGURE 36 | Relative error on fluorescence line height estimated, ρ_{w_fluo} (685), estimated using the oxygen B-band spectral optimization method (red curves) and the standard baseline method (black curves). (Left) Case 1 waters; (Right) Case 2 waters with sediment concentration *S* of 2gm^{-3} and CDOM absorption coefficient a_{CDOM} of 0.2 m^{-1}. Aerosol optical thickness is 0.2 and aerosol scale height is 1 km (unknown, fixed at 0.5 km). The relative errors are much reduced for all situations when using the optimization scheme. The standard baseline scheme yields comparable results in Case 2 waters only when chlorophyll concentration is >1 mgm^{-3}.

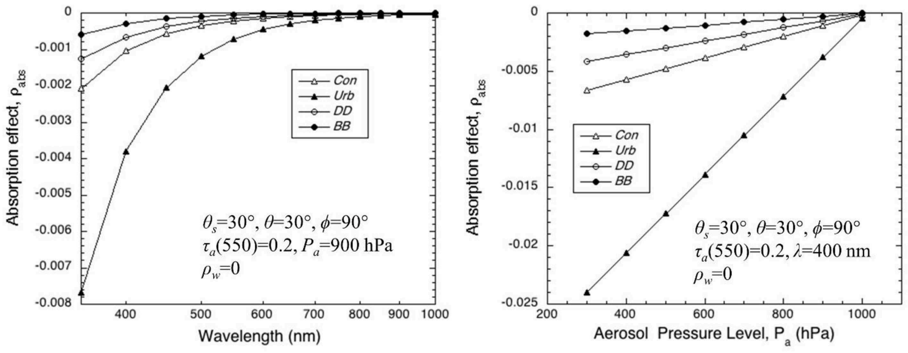

FIGURE 37 | Aerosol absorption effect as a function of wavelength (left) and aerosol pressure level (right) for continental (Con), urban (Urb), desert dust (DD), and biomass burning (BB) aerosol models. Solar and viewing zenith angles are 30°, and relative azimuth angle is 90°. Aerosol optical thickness is 0.2 at 550 nm, and surface reflectance is null. In the left panel, aerosols are located at 900 hP$_a$, and in the right panel wavelength is 400 nm. The absorption effect increases in magnitude with decreasing wavelength and aerosol pressure level (i.e., aerosols higher in the atmosphere).

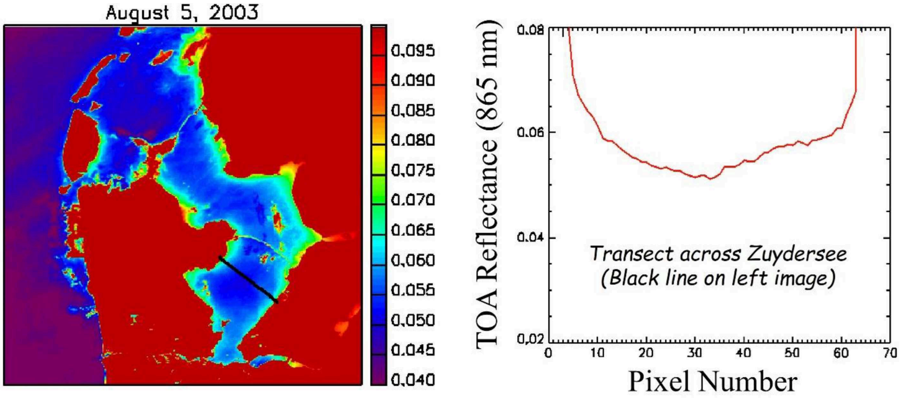

FIGURE 38 | Left: Image of TOA reflectance at 865 nm acquired by MERIS (1 km resolution) over the Netherlands on 5 August 2003. Right: Reflectance along the black line in the image. The measured signal over the Zuydersee is enhanced by reflected light from vegetated areas surrounding the target pixel (band of higher TOA reflectance values within 5–10 km of the coast).

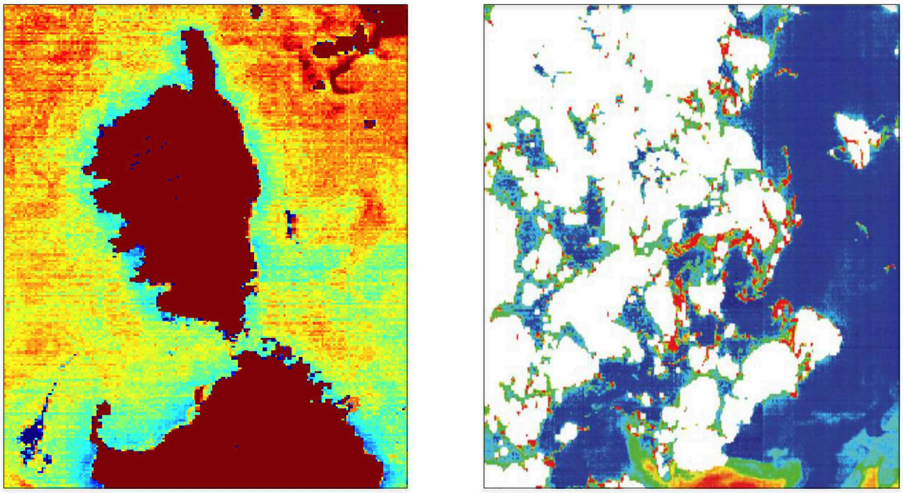

FIGURE 39 | Marine reflectance at 560 nm retrieved from MERIS imagery acquired at 1 km resolution on 4 March 2003 over the Mediterranean Sea (left) and on 4 July 2008 over the Beaufort Sea (right). Standard MEGS processing was used. Values are anomalously low (blue/green pixels) over a distance of more than 10 km along the coast of Corsica and Northern Sardinia (left), and anomalously high (green/red pixels) near sea ice (right). This is attributed to the adjacency effect in the near infrared bands used for the correction of aerosol scattering (see text for details).

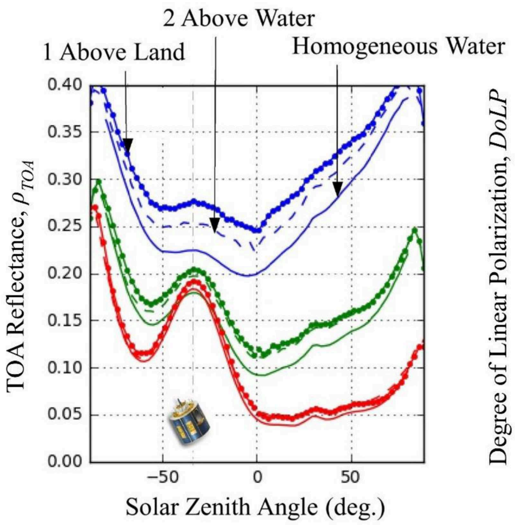

FIGURE 40 | TOA reflectance and degree of polarization of a water target located 5 km from a linear coastline as a function of solar zenith angle. Viewing zenith angle is 30°. The land reflectance is 0.8 (snow). Results are displayed at 380, 500, and 800 nm in the principal plane of the Sun for the sensor above water and above land, and assuming no adjacency effect (homogeneous water). Details about simulations are given in the text. Due to adjacency effects, the TOA reflectance is increased and the degree of polarization decreased, especially at 380 nm where atmospheric scattering is effective.

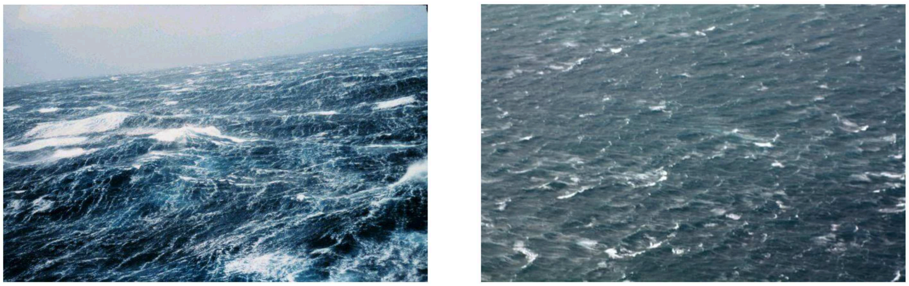

FIGURE 41 | Examples of seas with whitecaps, showing various stages of wave breaking with surface foam, streaks, and underwater bubble plumes. The presence of whitecaps changes dramatically the aspect of the surface and its brightness.

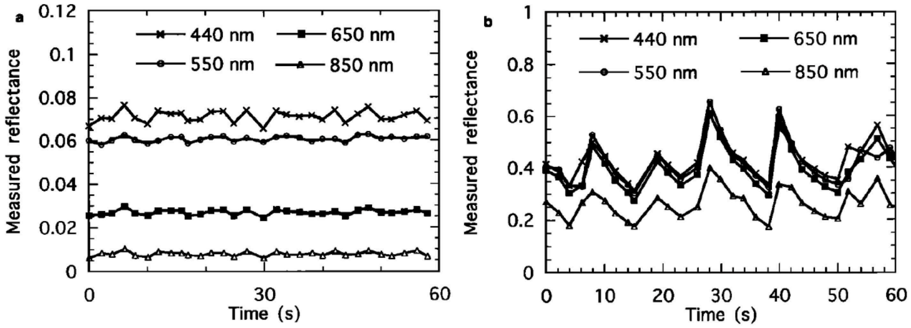

FIGURE 42 | Time series of measured surface reflectance at 440, 550, 650, and 850 nm in the surf zone, in the absence and presence of breaking waves, left and right, respectively. When waves break, air is trapped and injected below the surface, creating surface foam and underwater bubble plumes, increasing substantially surface reflectance. Reproduced with permission from Wiley (After Frouin et al., 1996).

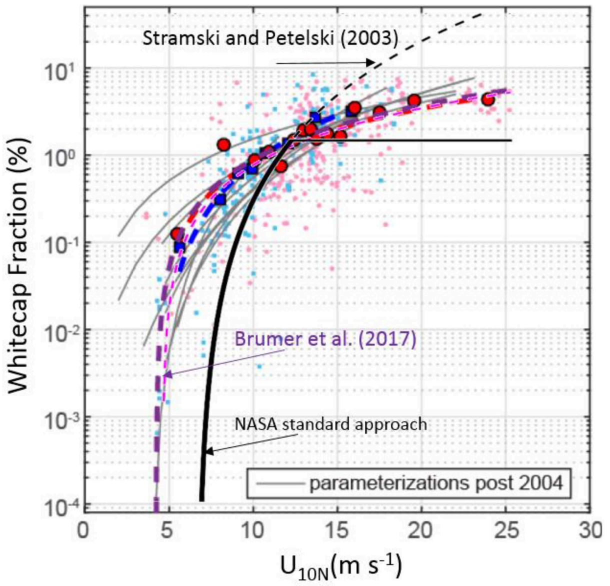

FIGURE 43 | Measurements of "equivalent neutral" wind speed at 10 m (U10N) and whitecap fraction (W) from the Southern Ocean Gas Exchange (blue) and HiWings (red) campaigns. Larger dots represent data binned over wind speed ranges and the dotted red line represents the mean from both campaigns. Overlaid on this plot (thick black curve) is the relation based on Stramska and Petelski (2003) that is used by NASA in the standard atmospheric correction algorithm. (Adapted from Brumer et al., 2017).

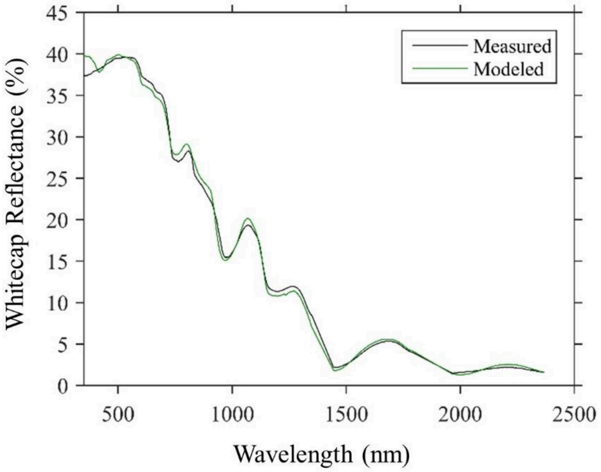

FIGURE 44 | Measured reflectance spectrum of intense breaking waves from a ship bow-wake showing the non-linearity of reflectance in near short-wave infrared wavelengths. The overall shape can be modeled using the absorption spectrum of liquid water. (Modified from Dierssen et al., 2019, this issue.)

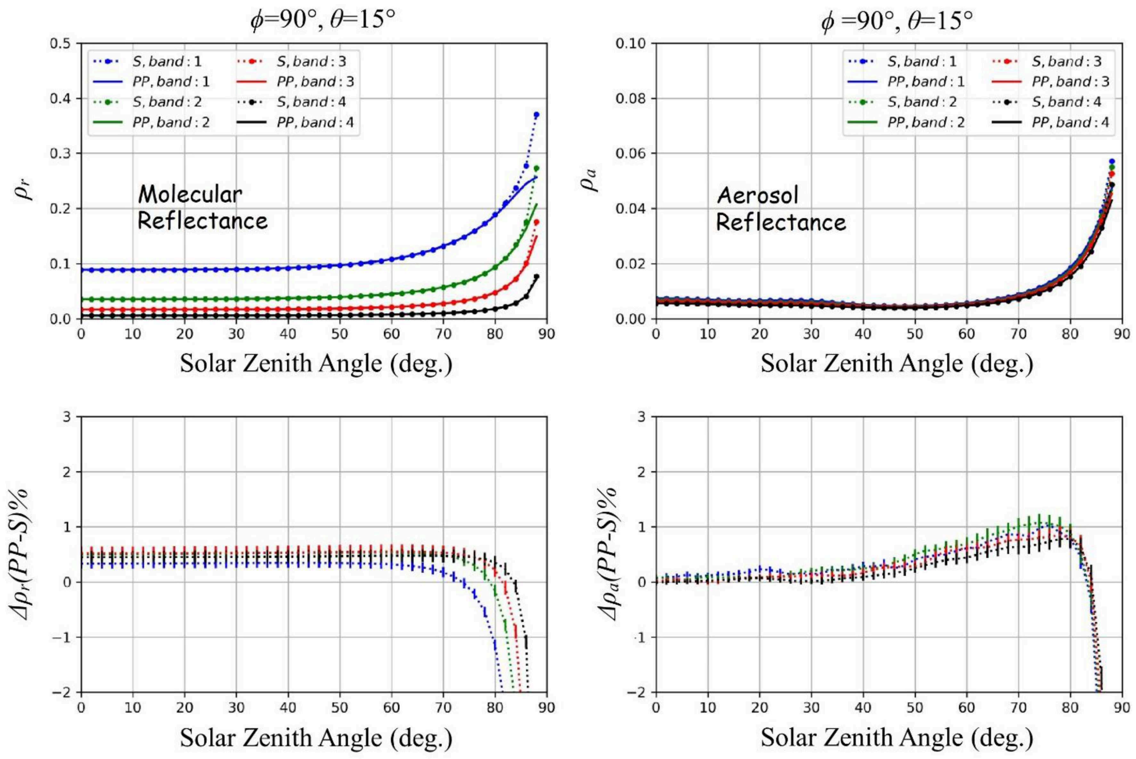

FIGURE 45 | SMART-G simulations of ρ_r (Left) and ρ_r (Right) at 446, 558, 672, and 867 nm as a function of Solar zenith angle for a viewing zenith angle of 15°. Relative azimuth angle is 90°. Aerosols are of maritime type with optical thickness of 0.1 at 550 nm. Calculations are made using plane-parallel (PP) and spherical-shell (S) geometry (solid lines and dots, respectively). Top: Absolute values. Bottom: Relative difference between PP and S results.

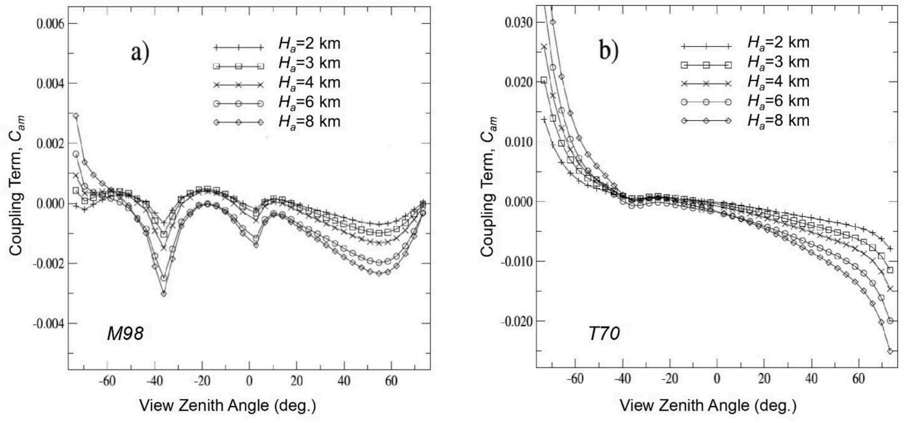

FIGURE 46 | Effect of aerosol altitude, H_a, on the coupling term, C_{am}, of the atmospheric reflectance due to interactions between aerosol and molecular scattering. Wavelength is 443 nm and aerosol models are M98 (a) and T70 (b). Aerosol optical thickness is 0.1 at 865 nm. Solar zenith angle is 36.2°. Results are for the principal plane (negative zenith angles correspond to backscattering). The case of $H_a = 8$ km corresponds to homogeneously mixed aerosols and molecules. Reproduced with permission from the University of Lille, France (After Tieuleux, 2002).

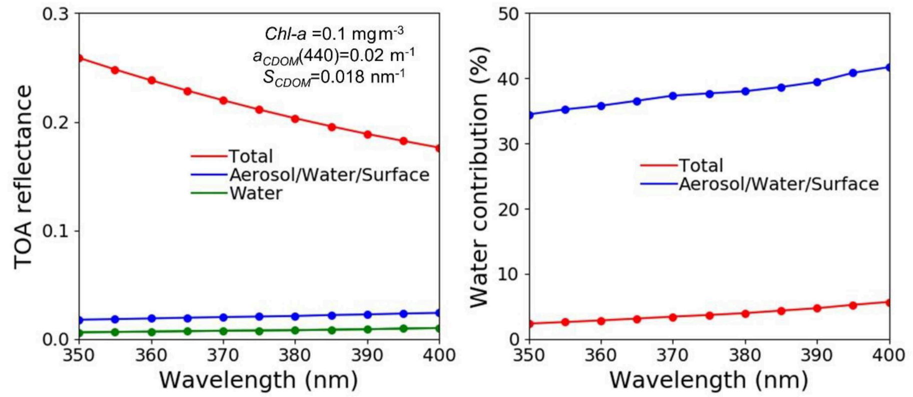

FIGURE 47 | (Left) Simulated TOA reflectance (total, corrected for molecular effects, and water body) for waters with chlorophyll concentration of 0.1 mgm^{-3} and CDOM absorption of 0.02 m^{-1} at 440 nm. Spectral slope of CDOM absorption is 0.018 nm^{-1}. Aerosols are of maritime type with optical thickness of 0.2 at 550 nm and scale height of 2 km, and wind speed is 7 m s^{-1}. Solar zenith angle is 30°, viewing zenith angle is 15°, and relative azimuth angle 90°. (Right) Contribution (in %) of the water signal to the total TOA signal (red curve) and to the corrected TOA signal (blue curve).

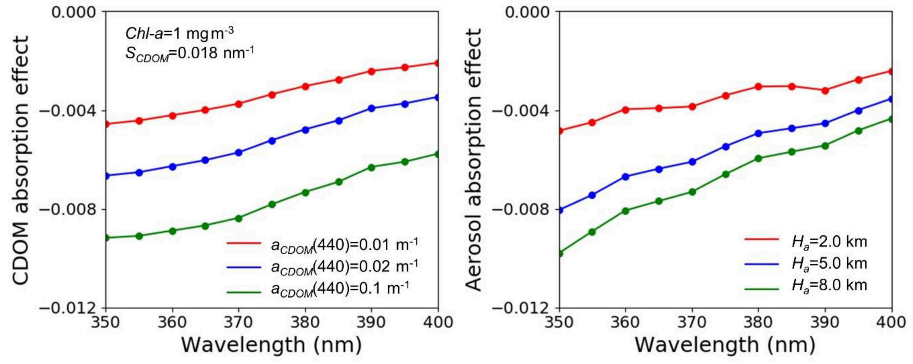

FIGURE 48 | (Left) Simulated CDOM absorption effect on water signal at top of atmosphere, for CDOM absorption coefficients of 0.01, 0.02, and 0.1 m^{-1}. Chlorophyll concentration is 1 mgm^{-3}. (Right) Simulated aerosol absorption effect for scale heights of 2, 5, and 8 km. Spectral slope of CDOM absorption, aerosol type and optical thickness, Sun/view geometry, and wind speed are the same as in **Figure 47**.
